# Emissions from Pre-Hispanic Metallurgy in the South American Atmosphere

**DOI:** 10.1371/journal.pone.0111315

**Published:** 2014-10-29

**Authors:** François De Vleeschouwer, Heleen Vanneste, Dmitri Mauquoy, Natalia Piotrowska, Fernando Torrejón, Thomas Roland, Ariel Stein, Gaël Le Roux

**Affiliations:** 1 Université de Toulouse, INP, UPS, EcoLab (Laboratoire Ecologie Fonctionnelle et Environnement), ENSAT, Castanet Tolosan, France; 2 CNRS, EcoLab, Castanet Tolosan, France; 3 School of Geosciences, University of Aberdeen, Aberdeen, United Kingdom; 4 Department of Radioisotopes, Institute of Physics, Silesian University of Technology, Gliwice, Poland; 5 Environmental Sciences Center EULA-Chile, University of Concepción, Concepción, Chile; 6 Geography, College of Life and Environmental Sciences, University of Exeter, Exeter, United Kingdom; 7 Palaeoenvironmental Laboratory (PLUS), Geography and Environment, University of Southampton, Southampton, United Kingdom; 8 NOAA/Air Resources Laboratory, R/ARL - NCWCP, College Park, Maryland, United States of America; New York State Museum, United States of America

## Abstract

Metallurgical activities have been undertaken in northern South America (NSA) for millennia. However, it is still unknown how far atmospheric emissions from these activities have been transported. Since the timing of metallurgical activities is currently estimated from scarce archaeological discoveries, the availability of reliable and continuous records to refine the timing of past metal deposition in South America is essential, as it provides an alternative to discontinuous archives, as well as evidence for global trace metal transport. We show in a peat record from Tierra del Fuego that anthropogenic metals likely have been emitted into the atmosphere and transported from NSA to southern South America (SSA) over the last 4200 yrs. These findings are supported by modern time back-trajectories from NSA to SSA. We further show that apparent anthropogenic Cu and Sb emissions predate any archaeological evidence for metallurgical activities. Lead and Sn were also emitted into the atmosphere as by-products of Inca and Spanish metallurgy, whereas local coal-gold rushes and the industrial revolution contributed to local contamination. We suggest that the onset of pre-Hispanic metallurgical activities is earlier than previously reported from archaeological records and that atmospheric emissions of metals were transported from NSA to SSA.

## Introduction

### 1. Background

Population expansion and territorial colonisation increased over the course of the last 5000 years in South America. Pre-Colombian civilizations flourished first in the Northern Andes and populations progressively migrated to the South. South American animal domestication and agriculture was followed by several periods of population expansion and metallurgical activities. In particular, copper extraction and smelting started in northwestern South America as well as in Argentina around 1400 BC and spread with the various South American civilizations (Chavin, Nasca, Tiwanaku, Inca). Incas also increasingly used silver, mainly around Peru, the Titicaca basin and Potosi (Bolivia). These changes in land use as well as the extraction and processing of metals up to the present day have released detectable amounts of trace elements into the atmosphere during the second half of the Holocene (*ca.* 4 kyrs cal BP to present).

Whereas South American metallurgy has been documented by archaeological finds, there is no record of how the resulting trace metals dispersed into the South American atmosphere through time and how far they have been transported away from production sites. To estimate the extent of anthropogenic trace metal emissions in South America, continuous and well-dated records of long-term metal deposition away from production centres are needed to highlight ancient metallurgical activities, given the absence/lack of archaeological evidence.

Our study of an ombrotrophic peat profile from Tierra del Fuego provides a continuous history of metallurgical activities in South America, and is therefore a valuable alternative to discontinuous archaeological findings. Andean trace metals of anthropogenic origin are found in our peat record from Tierra del Fuego, which suggests that they have been dispersed widely from their source areas. The data suggest for the first time that trace metals from pre-Hispanic and Hispanic Andean metallurgical activities were transported from North to South America, due to occasional North to South wind trajectories.

### 2. Scope

**Figure 1:**
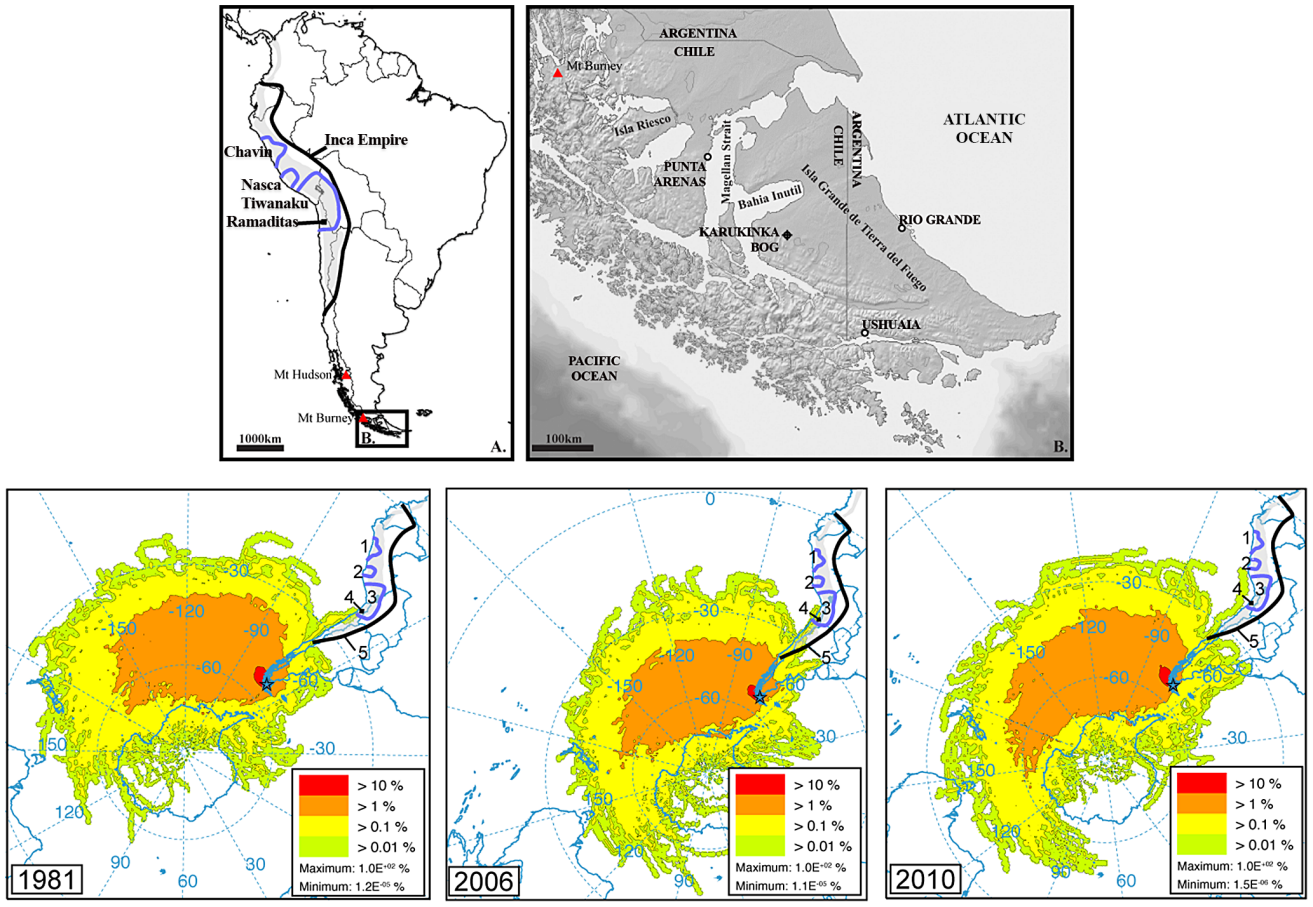
Upper Panel: A. Main polymetallic ores in South America (grey shaded area) and the extent of pre-Colombian civilisations and cultures discussed in the text (dark blue lines: 1-Chavin, 2-Nazca, 3-Tiwanaku; black square: 4-Ramaditas site black line: 5-Incas). B. Location of Karukinka bog in Tierra del Fuego. Lower Panel: back-trajectories for years when significant parcels of air masses travelled from NSA, specifically from the Inca, Tiwanaku and Nazca territories as well as from the Ramaditas site. Only the three most representative years are shown (see figure S1 for all years from 1948 to 2012). Other years (1997, 1999, 2000, 2008) are displayed in Fig. 3.

Continental archives, in particular raised/ombrotrophic peatlands or bogs have proven to be very useful in reconstructing past environmental changes and human activities [1]–[3]. Peat bogs are exclusively fed by atmospheric inputs and can therefore provide key archives of atmospheric metal deposition through time [3]. Long distance transport of trace metals is possible and hence suitable peat archive records can be used to identify the history of metallurgy even in remote areas [4]–[6]. In this study, we reconstruct the timing of metal deposition in SSA during the last 4200 years using a high-resolution record of metal/La ratios as well as Pb isotopes in a radiocarbon-dated peat core from Karukinka bog, located in the central western part of Isla Grande de Tierra del Fuego (Fig. 1). The ombrotrophic section of the core provides a reliable record of trace metallurgical activities throughout the Andes because 1/peat is mostly (>95%) composed of organic matter, hence trace to ultra-trace amounts of metals are detectable, 2/Modern-time particle back-trajectories supported by studies showing analogies between past and present climate [7]–[10] prove the feasibility of atmospheric transport from NSA to SSA and 3/the low level of local environmental and atmospheric contamination (no metallurgical activities before the 20^th^ century) provides ideal background conditions to record long-range trace metal deposition.

## Materials and Methods

### 1. Location

Karukinka Park is a protected area managed by the Chilean branch of the Wildlife Conservation Society (WCS) and located at the southwestern edge of Isla Grande de Tierra del Fuego. (Fig. 1). Access and coring permits were obtained by contacting Ricardo Muza at the WCS office in Punta Arenas (http://www.karukinkanatural.cl/). The mean annual rainfall is 400 mm.yr^−1^ and the mean annual temperature is 5°C. The reserve contains numerous peat bogs dominated by *Sphagnum magellanicum* with a sporadic cover of *Marsippospermum grandiflorum* and *Empetrum rubrum.* Occasional pools on the peatlands contain *Sphagnum falcatulum* and are fringed by *Tetroncium magellanicum*. Dense stands of deciduous forests surround the peat bog ecosystems and are dominated by *Nothofagus pumilio*. Peat deposits have accumulated continuously in this zone since the early Holocene (*ca.* 10kyrs cal BP) [11], [12] Several bogs were identified as ideal dust traps because they are located on high altitude points in open areas solely dominated by *Sphagnum magellanicum* mosses. The site we sampled is a small 1-km diameter peat bog (S 53.86002°, N 69.57639°, 245 m a.s.l.) located at a relatively high altitude, 200 m aside from the main valley and 20 m above the main river. Given this, significant minerogenic input of river sediments is highly unlikely.

### 2. Coring

Peat profiles were recovered from Karukinka bog in February 2012 using a stainless steel Russian corer of 10 cm internal diameter and 50 cm length [13]. The barrel of the corer was cleaned between each section using deionized water. The cores were photographed, described, wrapped in plastic film and packed into PVC tubes, which were subsequently stored in wooden boxes and shipped to France. An overlapping core were collected in order to avoid any possible disturbance at the top and bottom of each master core section, which can occur because the tip of the corer can slightly disturb the directly underlying peat layer. However, this core was not analyzed because we did not detect any contamination in the master core. The maximum depth (bottom of the peat bog) was reached at 4.5 m. In this study, we have only analysed the ombrotrophic part of the section (0–344 cm, see section 4.1).

### 3. Subsampling, depth correction and density calculation

A clean slicing and sub-sampling procedure was applied following published guidelines [14], [15] to ensure minimal contamination and disturbance, whilst maintaining the highest sampling resolution. Samples were handled with powder free latex gloves. The cores were frozen at −20°C, unpacked and sliced contiguously at 1 cm resolution using a stainless steel band saw in a room specifically designed to process pristine samples. Each slice was cleaned with mQ (18 mega Ohm clean) water. The edges of each semi-cylindrical slice were then removed and the slices were sub-sampled using ceramic knives and plastic cutting boards. The dimensions of the still-frozen samples dedicated to geochemistry were measured using a vernier caliper in order to know the exact thickness of the sample and the loss of material due to each cut. The thickness of each sample ranges from 0.8 to 1.3 cm with 72% of the samples being 1.0±0.1 cm thick. The average loss on each cut (i.e. [total core length - cumulative sample thickness]/Nr of samples) is 0.18 cm. We have taken this loss due to cutting into account and reassessed the exact mid-point depth of each sample. All the subsamples were finally stored in a freezer at −20°C.

According to literature recommendations [15], the frozen samples dedicated for geochemistry were also measured for their lateral dimensions in order to accurately calculate their volume. These frozen samples were then freeze-dried to remove their water. The resulting dry samples were subsequently weighed and their densities calculated by dividing their dry weight by their respective volume.

### 4. Macrofossil analysis and selection for radiocarbon measurement

Plant macrofossil samples (5 cm^3^) were boiled with 5% NaOH and sieved (mesh size 180 µm). Macrofossils were scanned using a binocular microscope (×10–×50), and identified using an extensive reference collection of type material collected during fieldwork in the study region in 2012. Plant macrofossils were assessed using the Quadrat and Leaf Count (QLC) method [16]. Volume percentages were estimated for all components with the exception of seeds, macroscopic charcoal particles, fungal fruit bodies and minerogenic grains, which were counted and expressed as the number (n) present in each sub-sample. The selection of plant macrofossils in 8 samples for AMS radiocarbon dating was made following established protocols [17]. The selection of aboveground plant macrofossils ensures that accurate dates are assigned (i.e. free of rootlets and therefore of contamination).

### 5. Radiocarbon sample preparation and measurement

The macrofossils from the 8 samples selected according to literature recommendations [15], [18] for radiocarbon dating mainly consisted of *Sphagnum magellanicum* leaves and stems except the basal sample, which consisted of unidentified graminoids. The deepest dated sample is at 440.6 cm depth (below this depth and down to the base of the core at 450 cm, no datable macrofossils could be retrieved). Enough macrofossils (15–20 *Sphagnum* stems or graminoid fragments) were picked to ensure a minimum carbon mass of ca. 1 mg. All samples were prepared at the GADAM Centre (Gliwice, Poland) using acid-alkali-acid washing (to remove carbonate, bacterial CO_2_ and humic/fulvic acid), drying, combustion and graphitisation [19]. Radiocarbon concentrations were measured at the Rafter Radiocarbon Laboratory (Lower Hutt, New Zealand) and at DirectAMS Laboratory (Bothell, USA) following established protocols [20]. Results are reported in Table 1.

**Table 1:** Results of the AMS radiocarbon dating of 8 selected samples from Karukinka.

Sample	Sample	Sample ID	AMS Lab ID	Age	Error	Depth
Name	Composition			^14^C BP	yr	cm
KAR12-PB01/111	*S. magellanicum* stems, leaves & branches	GdA-2764	NZA-51256	921	20	113.6
KAR12/PB01B/131	*S. magellanicum* leaves & stems	GdA-3034	D-AMS 002878	1528	25	141.1
KAR12/PB01B/166	*S. magellanicum* leaves & stems	GdA-3035	D-AMS 002879	1962	26	185.1
KAR12/PB01/209	*S. magellanicum* leaves & stems	GdA-2870	NZA-52239	2181	23	241.5
KAR12/PB01B/241	*S. magellanicum* leaves & stems	GdA-3036	D-AMS 002880	2850	27	285.2
KAR12-PB01/287	*S. magellanicum* leaves	GdA-2765	NZA-51257	3759	24	343.9
KAR12-PB01/315	*S. magellanicum* leaves & stems	GdA-2871	NZA-52232	4482	26	380.2
KAR12-PB01/363	Unidentified graminoids	GdA-2766	NZA-51258	7078	32	440.6

### 6. Acid digestion in clean lab and dilutions

Dried samples were manually crushed using a clean agate pestle and mortar, which was rinsed between each sample using mQ water and *pro analysis* grade ethanol. They were then stored in 20-ml plastic vials. Approximately 100 mg of dried and homogenized peat from each interval was weighed out and digested in Teflon beakers in a clean room with class 100 flow benches. All acids used were of ultrapure (purchased Optima grade) quality. The digestion procedure consisted of three steps each succeeded by a slow evaporation at 55°C [21]: (1) a mixture of 0.5 ml HF and 2 ml 16M HNO_3_ was added and left on the hotplate at 110°C for 2 days; (2) 1 ml of H_2_0_2_ was added to react for 6 h at room temperature; (3) 2 ml of 16M HNO_3_ was added and left at 90°C for 2 days to finalize the digestion. Although peat is mainly composed of organic matter, a strong acid digestion is needed to dissolve any silica-based mineral residue that forms part of the remaining inorganic content (for peat bogs this is likely to be atmospheric dust) [21], [22]. The samples were subsequently dissolved in 2 ml of 35% HNO_3_, transferred into 15 ml polypropylene tubes (Falcon®), and further diluted with Milli-Q water up 14 ml. All samples were stored in a fridge for future analyses.

### 7. Q-ICP-MS analyses

After proper dilution Cu, Pb and La concentrations were measured on a Quadrupole ICP-MS (Agilent Technologie 7500 ce) at the Observatoire Midi Pyrénée, Toulouse. The ICP-MS was calibrated using a synthetic multi-element standard, which was run every 8 samples, while an In-Re solution was used as an internal standard. The accuracy of the analyses was assessed by analysis of 3 international certified reference materials (SRM1947-peach leaves; SRM1515-apple leaves and GBW-07063-bush branches and leaves) and is reported in Table 2. The reproducibility, determined by repeat analyses (n = 3) of 1 CRM and 2 peat samples, is better <5% for Cu and Pb, and <12% for Sb, Sn and La. The blanks for all elements considered here, are <0.01%. Final dilution factors were approximately 700.

**Table 2:** Cu, Pb and La determined by Q-ICP-MS compared to certified values of SRM1947, SRM1515 and GBW-07063 (mean ± standard deviation, mg.kg^−1^).

	Measured	Certified
**GBW-07063 (n = 2)**		
** Cu**	6.4±1.0	6.6±0.8
** La**	1.1±0.6	1.3±0.06
** Pb**	49±1.4	47±3.0
** Sb**	0.1±3.7	0.1±0.01
**SRM1947 (n = 2)**		
** Cu**	3.6±0.6	3.7±0.4
** Pb**	0.7±0.4	0.87±0,03
**SRM1515 (n = 3)**		
** Cu**	6.3±0.8	5.6±0.24
** Pb**	0.45±1.5	0.47±0.02

La results are not certified for SRM1947 and SRM1515 hence not reported. Sn has no certified values. However, the good analytical reproducibility certifies the analytical quality of Sn measurement.

### 8. HR-ICP-MS

After the measurements of the Pb concentration by Q-ICP-MS, mother solutions of selected samples were sub-sampled and diluted to adjust the Pb concentration to 500 mg.kg^−1^ prior to analyses by HR-ICP-MS at the *Observatoire Midi-Pyrénées*, Toulouse [23]. A 500-mg.kg^−1^ SRM 981 standard solution was used to control and correct mass bias by standard bracketing of every sample. ^206^Pb/^207^Pb ratios in the NIMT peat standard were 1.175±0.001 (n = 4) compared to the reported value of 1.1763±0.0004 [24].

### 9. Sample preparation for tephra analysis

Based on the visual examination of the core as well as the lead isotope profile, 3 samples (237.5 cm, 34–301 yrs cal BC; 347 cm, 2354–2130 yrs cal BC; and 428 cm, 5745–5327 yrs cal BC) were processed to determine their potential volcanic origin by identifying glass shards. Dry samples were burned in a muffle furnace (550°C, 4 h) and ash residues were fine-sieved with the 10–125 µm fractions mounted in Canada Balsam on glass slides. Plane-polarised light, at ×100–400 magnification, was used to differentiate between tephra and other minerogenic material and 300 shards/grains were counted to determine their respective proportion. Fresh glass shards were identified and photographed.

### 10. Generation of the age-depth model

**Figure 2:**
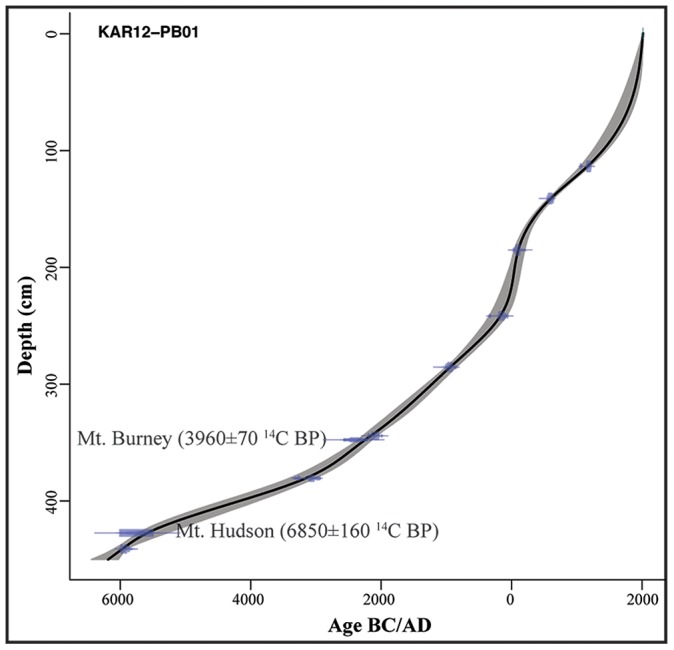
Age-depth model for the Karukinka peat core. Maximum likelihood ages are expressed in years Anno Domini (AD) and Before Christ (BC). The Hudson and Burney tephras are also used as chronological markers.

The 8 radiocarbon dates as well as two eruptions from Mt. Hudson (428 cm depth, 5745–5327 yrs cal BC) and Mt. Burney (347 cm depth, 2354–2130 yrs cal BC) were used as chronological time markers with the top of the core set to year 2012 AD (when it was recovered). CLAM software [25] was used to construct a cubic smooth spline age-depth model using the SHCal04 calibration curve [26]. The age-depth model is presented in Fig. 2. Based on 10000 iterations minimum and maximum ages for 2 sigma confidence interval were determined for each sample, as well as maximum likelihood age point estimates based on the weighted average of all generated age-depth curves. Maximum likelihood ages are given with the geochemical data in Table 3.

**Table 3:** Cu, Sn, Sb, Pb and La concentrations as well as ^206^Pb/^207^Pb isotopic ratios in the Karukinka core.

Depth	Density	Calendar Age	Cu	Sn	Sb	Pb	La	^206^Pb/^207^Pb	2σ
cm	(g.cm^−3^)	(BC/AD)	(mg kg^−1^)	(mg kg^−1^)	(mg kg^−1^)	(mg kg^−1^)	(mg kg^−1^)		
1.3	0.04	2010	1.59	0.04	0.02	0.14	0.14		
8.7	0.05	1998	0.99	0.02	0.02	0.14	0.07		
15.9	0.05	1985	0.84	0.03	0.02	0.28	0.25	1.181	0.001
22.1	0.05	1972	0.84	0.02	0.02	0.27	0.08		
28.0	0.10	1957	0.55	0.04	0.01	0.31	0.08		
30.8	0.14	1949	0.84	0.02	0.01	0.48	0.55	1.182	0.003
34.8	0.19	1936	0.89	0.02	0.01	0.20	0.20	1.179	0.0001
36.0	0.15	1932	0.40	0.03	0.01	0.21	0.07		
42.0	0.15	1910	0.46	0.01	0.01	0.19	0.06		
50.4	0.07	1874	0.85	0.01	0.01	0.12	0.15	1.195	0.0003
54.3	0.14	1853	1.14	0.06	0.02	0.23	0.30		
58.0	0.10	1831	0.81	0.01	0.01	0.15	0.15	1.185	0.004
62.0	0.09	1805	1.20	0.02	0.01	0.17	0.16		
66.0	0.06	1776	1.91	0.01	0.01	0.11	0.11		
72.6	0.06	1722	0.69	0.13	0.04	0.10	0.10	1.191	0.001
79.9	0.08	1653	0.99	0.01	0.01	0.15	0.20	1.193	0.001
84.1	0.09	1607	1.39	0.03	0.01	0.16	0.20	1.188	0.003
92.1	0.08	1509	2.24	0.02	0.01	0.17	0.33	1.190	0.002
96.1	0.10	1454	2.38	0.02	0.01	0.13	0.46	1.198	0.004
102.7	0.06	1355	2.12	0.03	0.02	0.14	0.63		
109.2	0.07	1244	2.48	0.03	0.02	0.22	0.63		
117.1	0.05	1093	3.63	0.02	0.02	0.12	0.54		
122.2	0.08	988	4.56	0.07	0.02	0.20	0.87	1.194	0.002
127.7	0.09	871	3.43	0.05	0.02	0.19	0.64		
135.3	0.09	713	4.03	0.08	0.05	0.19	1.14		
142.7	0.08	571	3.62	0.04	0.01	0.37	3.13	1.193	0.001
146.9	0.11	499	6.71	0.06	0.02	0.85	7.30	1.197	0.0002
158.0	0.13	339	15.28	0.15	0.07	1.59	15.19	1.198	0.001
161.8	0.12	293	21.53	0.11	0.10	2.00	14.76		
164.4	0.11	264	21.55	0.15	0.12	1.90	14.72		
168.0	0.10	228	17.10	0.12	0.08	1.56	11.37		
171.8	0.11	194	24.03	0.13	0.08	1.36	10.32	1.197	0.002
175.4	0.08	165	22.19	0.06	0.07	1.28	9.35		
179.0	0.07	140	24.72	0.05	0.08	1.23	7.62		
182.8	0.09	117	10.69	0.07	0.06	0.80	6.26		
190.0	0.08	83	18.05	0.05	0.06	0.91	5.37		
195.4	0.07	64	14.49	0.05	0.05	0.93	5.73	1.190	0.001
201.3	0.06	46	12.46	0.05	0.07	0.68	4.81		
207.8	0.08	29	12.91	0.05	0.05	0.87	6.66		
215.2	0.07	6	9.1	0.1	0.1	1.4	5.4		
221.9	0.08	−21	13.86	0.07	0.04	1.39	6.85		
227.4	0.06	−50	21.63	0.26	0.07	1.63	7.68		
231.5	0.06	−77	20.04	0.09	0.06	1.33	6.21	1.196	0.002
237.5	0.16	−128	24.53	0.09	0.06	1.50	9.00	1.198	0.003
244.3	0.13	−204	4.49	0.04	0.03	0.44	5.64		
250.2	0.09	−285	9.26	0.04	0.04	0.80	6.35		
253.1	0.14	−330	4.68	0.03	0.03	0.40	4.78	1.199	0.001
258.7	0.12	−424	5.30	0.04	0.03	0.41	4.36		
264.8	0.10	−535	5.19	0.03	0.03	0.33	2.96		
270.2	0.10	−639	5.78	0.05	0.03	0.41	3.79	1.196	0.001
274.0	0.09	−714	5.64	0.04	0.03	0.39	3.98		
279.7	0.10	−828	3.0	0.1	0.0	0.4	2.9		
285.2	0.09	−937	2.35	0.02	0.02	0.32	2.03	1.198	0.001
289.3	0.09	−1017	2.52	0.03	0.02	0.31	1.82		
293.4	0.14	−1096	3.49	0.08	0.04	0.25	2.04		
300.9	0.10	−1239	5.88	0.03	0.04	0.41	2.15	1.196	0.003
304.9	0.09	−1316	6.28	0.04	0.03	0.21	1.41		
308.6	0.08	−1387	4.44	0.02	0.02	0.26	1.45		
312.2	0.13	−1457	4.63	0.07	0.06	0.58	2.87	1.195	0.002
320.5	0.10	−1623	6.46	0.04	0.04	0.27	1.38		
325.6	0.13	−1729	9.90	0.07	0.06	0.54	2.47		
330.8	0.11	−1842	6.31	0.06	0.04	0.54	2.78	1.199	0.002
334.8	0.12	−1932	6.25	0.06	0.05	0.68	3.29		
339.8	0.12	−2050	6.60	0.04	0.05	0.48	2.79		
343.9	0.12	−2152	6.62	0.09	0.04	0.85	3.56		

Sample depths and maximum likelihood calendar ages (positive values are ages Anno Domini, negative values are ages Before Christ) are also reported.

### 11. Back trajectory calculation

The Hybrid Single Particle Lagrangian Integrated Trajectory (HYSPLIT) model [27] has been used to run a series of back-trajectories from January 1, 1948 until December 31, 2012. The model is driven by meteorological data fields from the NCEP/NCAR Reanalysis Project (http://ready.arl.noaa.gov/gbl_reanalysis.php and ftp://arlftp.arlhq.noaa.gov/archives/reanalysis/) [28]. The tools (http://www.meteozone.com/home/tutorial/html/traj_freq.html) used to generate the trajectories are publicly available for the scientific community to reproduce the results. Previous observational and modelling palaoclimate studies have shown strong analogies between present and past climate and wind conditions [7]–[10], and that climate variability is partly controlled by modes or short-term pluriannual to decadal-scale oscillations [7]. Therefore, our calculations, which cover more than 60 years, encompass these climatological drivers. The calculation includes back-trajectories initialized from the measurement site every six hours and the duration of each trajectory is set to 15 days. The trajectory starting heights are set to 500 m above ground level to represent air parcels within the planetary boundary layer reaching the measurement site. A total of 93440 trajectories were computed for this study. Once all the trajectories were calculated, the model counts the number of trajectories that fall within each grid cell from an arbitrarily set horizontal grid that covers the Southern Hemisphere. The grid resolution is 1.0 degree. The trajectory frequency (F) is just the sum of the number of trajectories (T) that passed through each (i, j) grid cell divided by the total number (N) of trajectories analyzed:
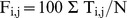



Note that all trajectories are counted once in the source location grid cell and once per intersecting grid cell.

**Figure 3:**
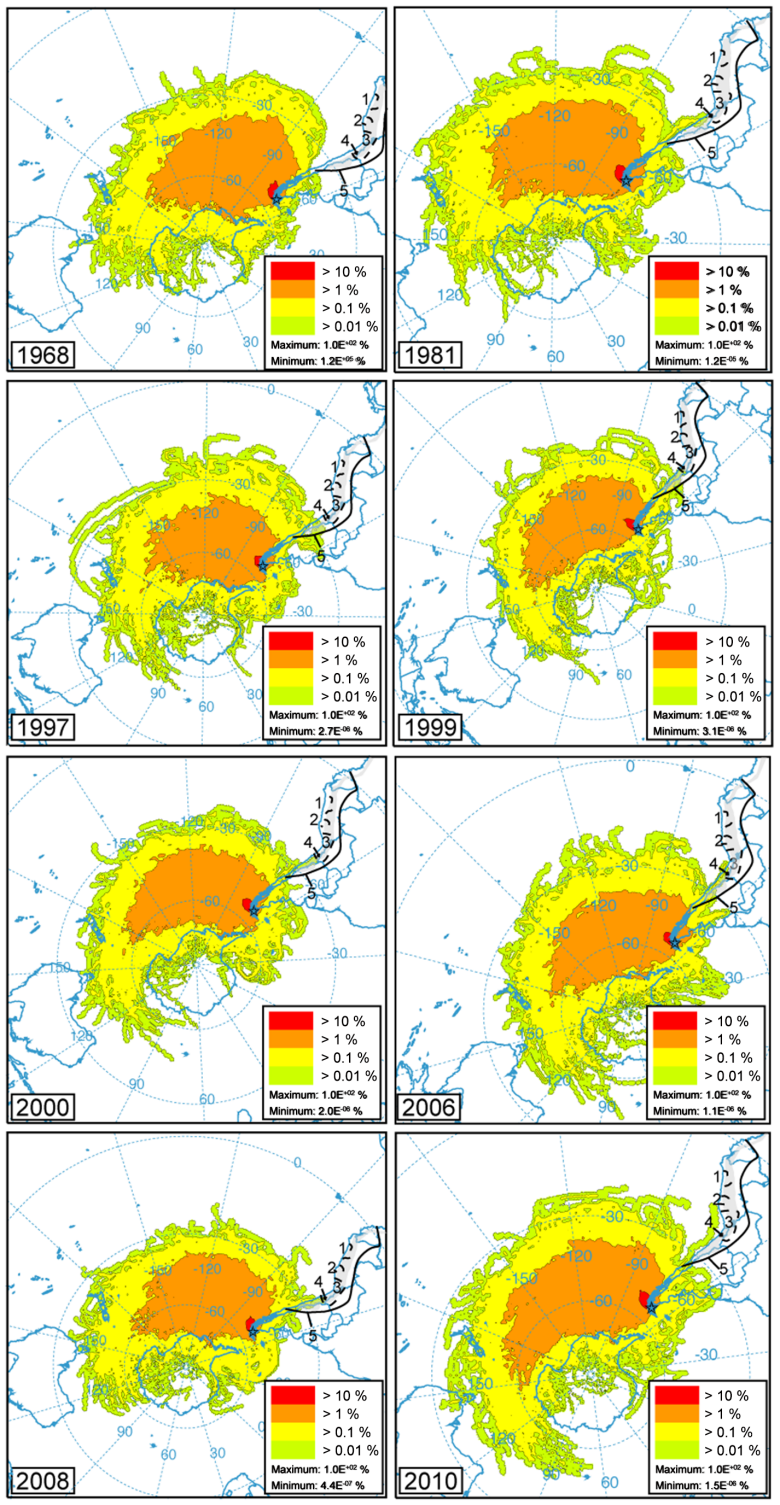
Back trajectory frequency corresponding to calendar years where air parcels are partially coming from NSA. The year 1968 is given as a comparison to show a year where no air back trajectory is coming from NSA (see figure S1 for all years from 1948 to 2012).

Out of the 64 years investigated in this model (from 1948 to 2012, figure S1 for more information), air parcels coming partially from NSA and the pre-Hispanic metallurgical territories 7 times (Fig. 3). This represents more than 10% of the total period. As each of the peat slice from Karukinka represents several decades of peat accumulation, and assuming that the 64-year climatology used to drive the back-trajectories is representative of the atmospheric circulation of the past 4500 years, each slice from Karukinka is likely to capture several years where small but significant amounts of metals coming from Pre-Hispanic metallurgical territories are incorporated in the air parcels travelling to Tierra del Fuego.

## Results and Interpretation

### 1. Determination of the bog trophic status

**Figure 4:**
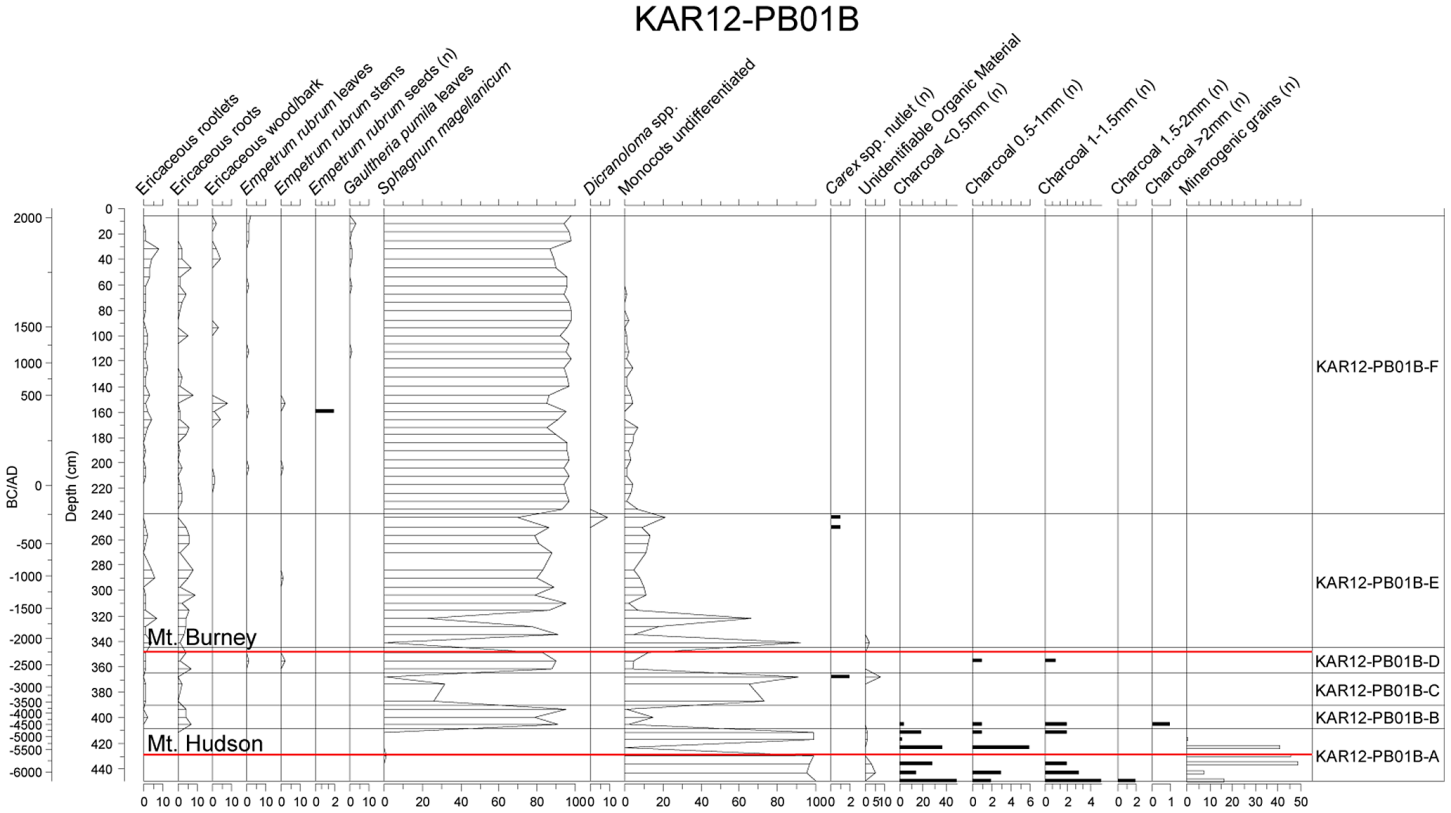
Detailed macrofossil age and depth profile for the Karukinka peat profile. Mineral grains are from the Hudson and Burney tephras (red lines).

Karukinka bog is located on a small hill beside the main Karukinka Valley. It consists of an open area where the surface vegetation is dominated by *Sphagnum magellanicum* mosses. The 5-cm resolution macrofossil diagram records a poor-fen stage (zone KAR12-PB01B-A from the base of the core (450 cm) to approximately 415 cm depth (4555–4215 yrs cal BC) and is composed of graminoid remains (Monocots undifferentiated). The presence of abundant macrofossil charcoal fragments indicates that local fires may have occurred during this period. The fen stage (Fig. 4 is followed by a transition to a bog (zone KAR12-PB01B-B) at 344 cm depth (2269–2057 yrs cal BC). This transition stage is however largely dominated by *Sphagnum magellanicum*. The ombrotrophic stage at 344 cm persists to the present, with a dominance of *Sphagnum magellanicum* (up to 95% of the total macrofossil assemblage). Moreover, from 344 cm to the surface, the peat bulk density is low (<0.2 g.cm^−2^), which also suggests ombrotrophic conditions spanning this depth interval. These low bulk density values indicate that minimal inwash of mineral material from lateral streams has occurred and therefore the peat deposits are likely to record predominantly low atmospheric dust inputs. Under these conditions, previous studies have shown that the elements analysed here (Pb, Cu, Sn, Sb) are not prone to post-depositional mobility and should therefore effectively record the history of metallurgy and other human activities [5], [29]–[32].

### 2. Tephra identification

Of the 3 samples submitted for petrographic analysis, tephra horizons were identifiable in two (347 cm depth, 2354–2130 yrs cal BC and 428 cm depth, 5745–5327 yrs cal BC). The remaining sample contained small numbers of tephra shards but did not exceed baseline levels, which are typically present in such close proximity to numerous volcanic sources.

**Figure 5:**
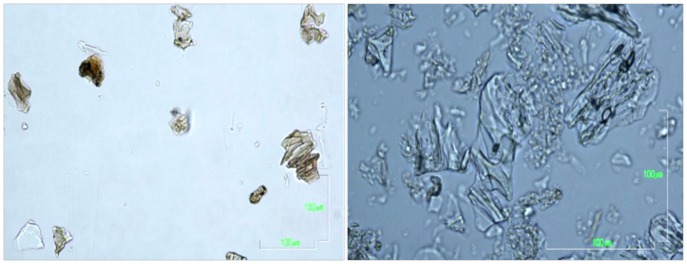
Picture of Mt. Hudson 5745–5327 yrs cal BC (428 cm depth, left) and Mt. Burney 2354–2130 yrs cal BC (347 cm depth right) shards found in Karukinka peat core.

The tephrochronology of SSA is relatively well known [33]. Subsequently, the two tephra horizons identified in the Karukinka peat core could be confidently attributed to known eruptions based on the characteristics of the shards present and their respective ages, as calculated from the age-depth model. The Mt. Hudson eruption was identified at 428 cm depth (5745–5327 yrs cal BC), with shard colour and morphology consistent with an eruption from the predominantly andesitic southern Volcanic Zone in which Mt. Hudson is located (Fig. 5). Conversely, the transparent, rhyolitic shards found at 347 cm were consistent with the Mt. Burney eruption (2354–2130 yrs cal BC) and the Austral Volcanic Zone in which the volcano is situated [33]. These tephra horizons have been identified in various peat cores from Southern South America [33]–[36]. The presence of a younger eruption from Mt. Burney reported in the literature at 358 cal BC –201 cal AD in Southern Patagonia [33] is not clear in the Karukinka peat sequence, since no significant glass shard concentrations were found in the layer corresponding to this time range.

### 3. Brief description of the raw geochemical results (Cu, Pb, La, ^206^Pb/^207^Pb) and calculation of Metal/La_UCC_ ratio

Copper and Sb concentrations remain below 7 mg.kg^−1^ from the base of the core to approximately 250 cm depth (Table 3). From 250 cm to 150 cm depth, the Cu concentration increases periodically, reaching more than 24 mg.kg^−1^ to finally decrease rapidly and remain low from 150 cm depth to the surface. Lead and Sn concentrations remain mostly below 1 mg.kg^−1^ except around 250 cm–150 cm where concentrations double and peak twice. Lanthanum concentration are stable between ca. 1 mg.kg^−1^ and 6.5 mg.kg^−1^ from 344 cm to 250 cm depth, and then increase twice from 3 mg.kg^−1^ to 15 mg.kg^−1^ between 250 cm to 150 cm, to then decrease below 1 mg.kg^−1^. These increases in metals and La between 150 and 250 cm are not interpreted here since the concentration profiles are prone to changes in the accumulation rates or in the influx of dust to the bog. In order to accurately interpret changes in metal inputs to the bog, we will be using Me/La ratios in the rest of the manuscript. Upper Continental Crust (UCC)-normalized metal/La ratios were calculated by dividing the metal/La ratio by the same ratio in the UCC, with 25 mg.kg^−1^, 17 mg.kg^−1^, 0.2 mg.kg^−1^, 5.5 mg.kg^−1^ and 30 mg.kg^−1^ as the UCC concentrations for Cu, Pb, Sb, Sn and La, respectively [37].

Lead isotopes fluctuate within a range typical for the Upper Continental Crust (1.18<^206^Pb/^207^Pb <1.21) with three slight shifts towards more radiogenic values around 425 cm, 351 cm and 195 cm depth. In shallower samples (above 60 cm depth), the ^206^Pb/^207^Pb shifts towards more radiogenic values, which are typical for a mix between anthropogenic and natural sources.

### 4. History of metallurgy as recorded in Karukinka bog

Whereas metallurgical activities occurred throughout South America since ca 2000 BC, there is no evidence that pre-Hispanic metal objects or artifacts reached Tierra del Fuego [38]. Hunters and gatherers as well as canoe-borne nomadic people from Tierra del Fuego were seemingly unaware of purified metals before the Argentinean and Chilean colonization of these territories occurred in the 19^th^ century [39], [40]. Hence, compared to the Northern Hemisphere and mainly Europe, Tierra del Fuego remained almost pristine and free of any local metal contamination until the last two centuries.

**Figure 6:**
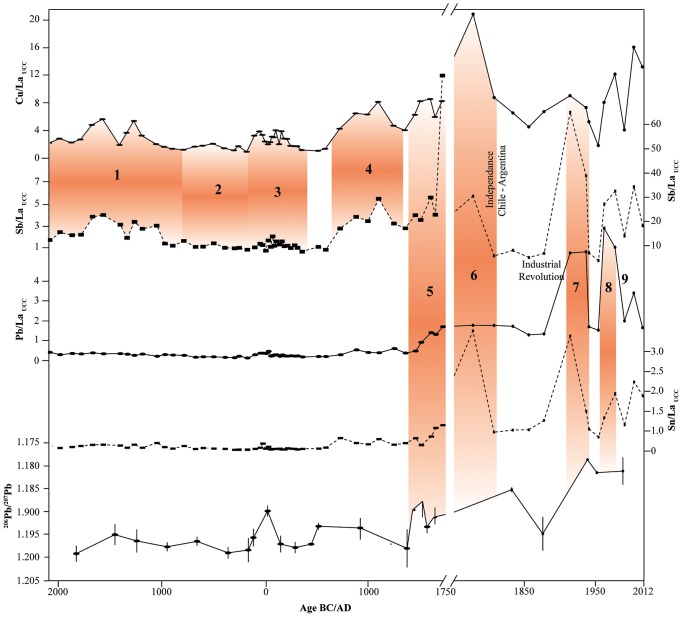
UCC-normalized Cu, Sb, Pb and Sn vs. La ratios [37] and ^206^Pb/^207^Pb isotopic ratios in the Karukinka peat core during the last 4200 yrs (ombrotrophic part of the core). Maximum likelihood ages are expressed in years Anno Domini (AD) and Before Christ (BC). The age scale is cut at 1750 AD and the Sb/La_UCC_ scale changes at 1750AD. 1. Onset of Cu metallurgy, 2. Chavin, 3. Nazca and Ramaditas, 4. Tiwanaku, 5. Inca (transition from Cu to Ag mining), 6. European Colonization, 7. Gold rush and bronze industry, 8. Leaded gasoline period, 9. Unleaded gasoline period (see text for details).

The upper part of the Karukinka peat bog (4200 years) is ombrotrophic as demonstrated by the plant macrofossil succession (see Figure 4) and therefore is a suitable archive of atmospheric metal deposition. By accurately analysing Pb, Cu, Sb, Sn, La, and Pb isotopes in peat samples using Q-ICP-MS and HR-ICP-MS, supported by a ^14^C age-depth model, we provide a unique history of anthropogenic activities and metallurgical developments in South America since the mid-Holocene (from *ca.* 4 kyrs cal BP) (Fig. 6). Modern-time back trajectory calculations further demonstrate that air parcels can be transported from the main metallurgical areas in NSA to SSA. The trajectory frequency plot confirms that this is the case for 7 times (1981, 1997, 1999, 2000, 2006, 2008, 2010) during the 64 investigated years. Accordingly, NSA, and especially northern Chile, southern Bolivia and southern Peru are potential source regions of metals found in Karukinka (Fig. 1). Assuming that the 64-year climatology is representative of the atmospheric circulation of the past 4200 years and as each peat slice from Karukinka represents several decades, each single peat slice is likely to capture several years where small but significant amounts of metals coming from pre-Hispanic metallurgical territories are incorporated in the air parcels travelling to Tierra del Fuego.

The lower part of the peat profile (6500 BC to 2200 BC) is not ombrotrophic and may have been exposed to local in-washed mineral inputs and changes in redox conditions. As we want to investigate atmospheric metal deposition, we therefore do not interpret this part of the profile in detail but focus our interpretations on the ombrotrophic part of the sequence (2200 BC to present day).

The oldest archaeological evidence for copper metallurgy dates back to ca. 1400 BC in Southern Peru and the following centuries saw its development mainly in the Bolivian Altiplano, Northern Chile but also in the Antofagasta and northeast Argentina regions. This is based on archaeological evidence for metal ore extraction and smelting, various ritual objects, artifacts and weapons [41]–[47]. In the Karukinka core, the Upper Continental Crust (UCC)-normalized Cu/La_UCC_ ratio increases starting at 2000 BC (ca. 600 yrs before the first archaeological evidence), suggesting that small but detectable amounts of Cu would be transported to SSA and that Andean Cu metallurgy may be older than previously reported. Antimony, associated with Cu ores such as tetrahedrite, also displays an increase in the Sb/La_UCC_ ratio. A shift in Pb isotopes can also be observed around the BC-AD transition. Pb metallurgy was not yet developed in South America, therefore this shift may be an early manifestation of metallurgy of unknown origin or a shift in Pb isotopes from a natural origin. An eruption from Mt. Burney with a large associated age range (358 BC–201 AD) has been reported in various Patagonian sites [33], [34] and could explain the shift observed in Karukinka. While volcanic eruptions can shift isotopic ratios [48], we found no volcanic material in this sample. Therefore, we interpret this shift as of an unknown anthropogenic origin, or caused by trace levels of Pb contained in the Cu ores that were processed by the Nazca populations at that time.

Civilizations such as the Chavin and Nazca are mainly known to have worked textiles and ceramics but they also processed copper [38], [42]. At the BC-AD transition, the Atacama Desert was the cradle of a new Cu metallurgical technique in the Ramaditas area, northern Chile, with wind being directed into partly opened furnaces to increase the temperature and melt the ores [49]. The subsequent increase in metal production, as well as to a certain extent Chavin and Nazca metallurgical activities, also emitted metals into the atmosphere. Increases in Cu/La_UCC_ and Sb/La_UCC_ between 80 BC and 200 AD in Karukinka and the modern-time back-trajectories indicate that some of these metals may have been transported south as far as Tierra del Fuego. Between 500 and 1000 AD, the Tiwanaku civilization spread through and dominated the South-Central Andes, the Titicaca Basin and northern Chile [50] and most likely increased the metallurgical activities in these areas [51]. This constitutes a turning point in South American metallurgy, with the development of smelting techniques (wax moulds and tin-copper alloys) as recorded by a significant increase in metal objects and artefacts at this time in northern Chile, particularly in San Pedro de Atacama [48]. Between 650 and 850 AD, metallurgical activities also developed in northwest Argentina [42], [52]. This boom in Cu metallurgy is shown by a drastic increase in the Cu/La_UCC_ and Sb/La_UCC_ ratios in Karukinka, starting around 550 AD and culminating at 1100 AD. Interestingly, an increase in Cu/La_UCC_ during this period was reported in an ice core collected in Bolivia [1], but the resolution was too low to make a conclusive interpretation.

Copper production intensified under the control of the Incas (1480–1532 AD) [54]. During the Inca domination and during the Hispanic colonization, northwest Argentina and northern Chile remained important copper production areas [52], [53]. The Karukinka peat record highlights the period of Inca copper metallurgy with an increase of Cu/La_UCC_ and Sb/La_UCC_ starting at 1400 AD and persisting until ca. 1600 AD. At the same time, the mining of silver in the Andes triggered a substitution of copper to silver smelting from ca. 1450–1533 AD (i.e. during the Inca Period) around Peru, the Titicaca basin and Potosi, Bolivia [51]. In order to reconstruct the Ag mining history, we report Pb and Sn data as these metals have been shown to be associated with Ag ores, for example in Potosi [51]. An increase in Pb/La_UCC_ and Sn/La_UCC_ from ca. 1400 to ca. 1800 AD indicates that Pb and Sn were dispersed into the atmosphere and transported to SSA during the silver mining era. This is consistent with similar increases in Pb and Sn in NSA lake sediments [51], [53] as well as the Inca and European Ag production, especially at the famous Potosi mine. The increase in Ag and Pb smelting is also clearly marked in Karukinka by a shift in the ^206^Pb/^207^Pb ratio towards values of ca. 1.19, corresponding to isotopic signatures found in Bolivian and Peruvian ores [55], [56].

Although the most recent section of the Karukinka core is not as well chronologically constrained as the pre-Hispanic section, we can identify excursions in the metals/La_UCC_ ratios and Pb isotopic profiles, which match historical data from the 18^th^ to 20^th^ century. We therefore present a tentative interpretation for this section of the profile. The early 18^th^ century sees a depletion in Ag resources and a transition to Sn mining in the Potosi area [51], which could explain the increase in Sn/La_UCC_ but also Cu/La_UCC_ in Karukinka, reflecting the increase of bronze alloy production. The 19^th^ century is marked by a period of unrest associated with the independence of South American countries, especially Chile and Argentina and is associated with a decrease of Cu and Ag production. In Karukinka, the decrease of Cu/La_UCC_ and Sn/La_UCC_ around 1750–1820 AD, the slight decrease in Pb/La_UCC_ and Sb/La_UCC_ around 1800 AD, and the shift in Pb isotopic values could reflected this period of unrest. Copper and Pb production remained low in South America until 1920–1930 AD [1]. The Fuegian gold rush in 1903–1908 AD [57] and two phases of coal exploitation – during the first half of the 19^th^ century and from 1980 onwards [58] - lead to the first emissions of local anthropogenic metal particles in Tierra del Fuego. These events seem to be recorded in the Pb/La_UCC_ profile, which increased drastically around 1900 AD. This regional ‘industrial revolution’ persisted into the 20^th^ century and saw the introduction of leaded gasoline as well as the intensification of lead metallurgy until 1980 [1]. This would be recorded by an increase in the Pb/La_UCC_ at the beginning of the second half of the 20^th^ century to a maximum around 1970–1980 and by a shift in the Pb isotopic signatures towards less radiogenic values. The last period of atmospheric metal deposition recorded in Karukinka could be the introduction of unleaded gasoline in the late 1980’s [59], which is seen by a drastic decrease in Pb/La_UCC_ as well as a shift in Pb isotopes towards more radiogenic compositions, while the Cu/La_UCC_ does not decrease, as Cu production is still flourishing in South America [1].

## Conclusions

The Karukinka peat bog sequence records past metallurgical activities in South America, as the area was isolated from any local anthropogenic sources until the 19^th^ century. Based upon the excellent agreement between variations in UCC-normalized Cu, Pb, Sb and Sn to La ratios, modern-time back-trajectories and historical data, we conclude that Cu metallurgy predates archaeological evidence and that NSA atmospheric emissions from pre-Hispanic civilizations through to the Industrial Revolution are recorded in SSA.

## Supporting Information

Figure S1 Back trajectory frequency corresponding over Tierra del Fuego 1948 to 2012.
(PDF)
