# Supplementary material for: Emissions from Pre-Hispanic Metallurgy in the South American Atmosphere
Source: PLoS One. 2014 Oct 29;9(10):e111315. doi: 10.1371/journal.pone.0111315 (PMC4213032; doi:10.1371/journal.pone.0111315)
Supplement: Figure S1 — Back trajectory frequency corresponding over Tierra del Fuego 1948 to 2012. (PDF) [file pone.0111315.s001.pdf]

Trajectory Frequency Plot year 1948

Values (%) averaged between 0 m and 2000 m

Integrated from 0000 00 to 0000 00 00 (UTC)

Freq Release started at 0000 00 00 (UTC)

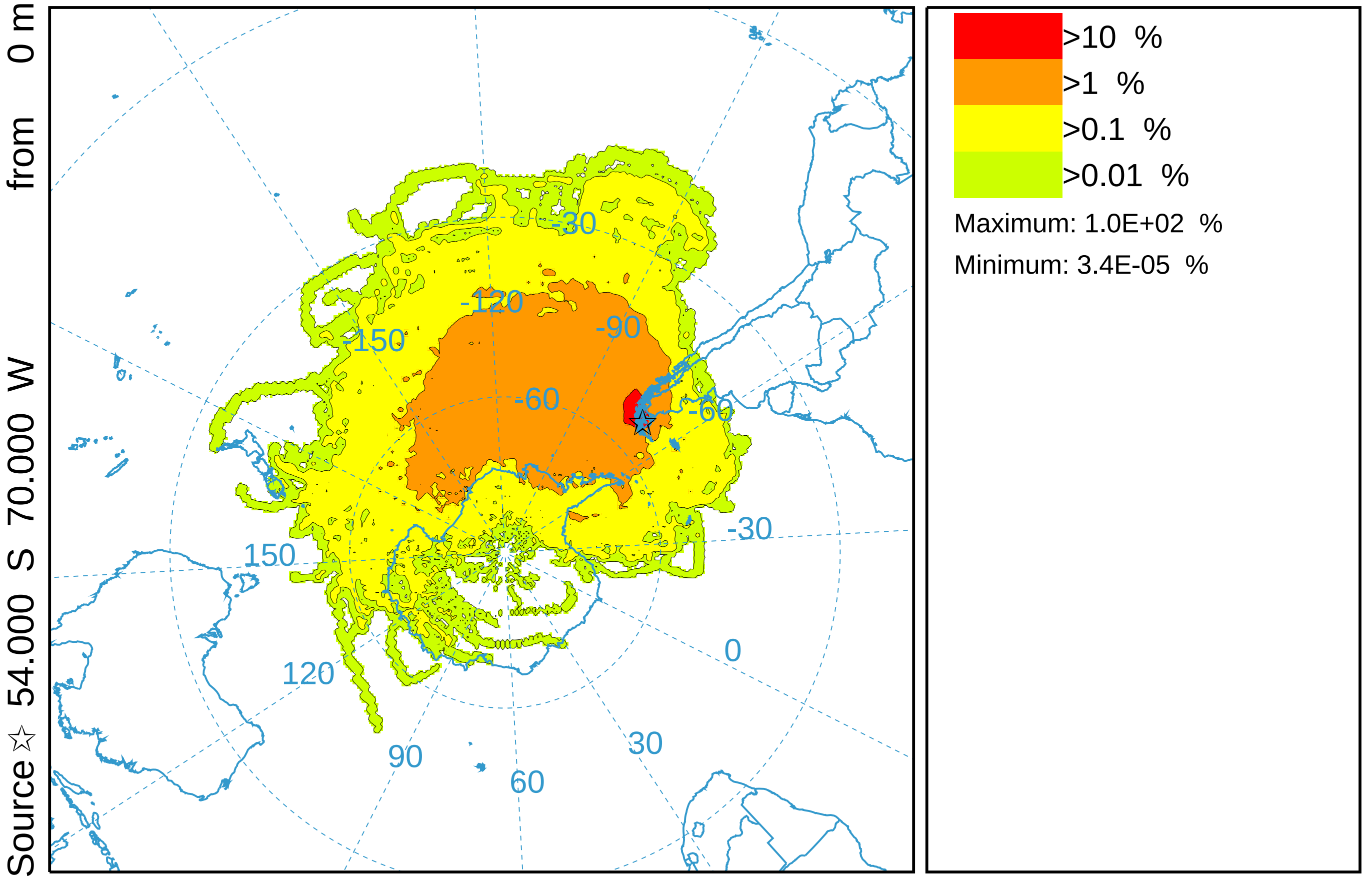

METEOROLOGICAL DATA

Trajectory Frequency Plot year 1949

Values (%) averaged between 0 m and 2000 m
Integrated from 0000 00 to 0000 00 00 (UTC)
Freq Release started at 0000 00 00 (UTC)

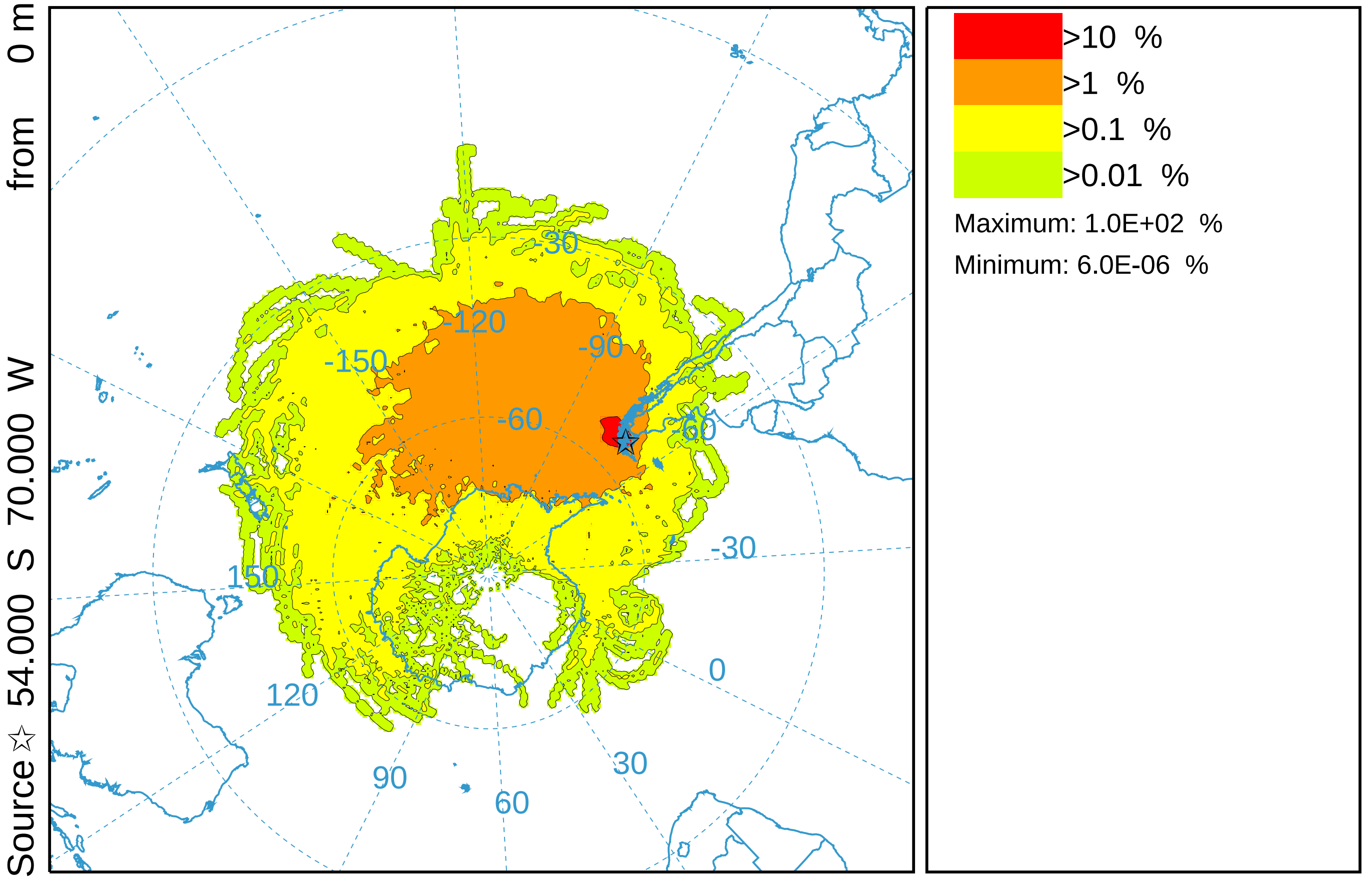

METEOROLOGICAL DATA

Trajectory Frequency Plot year 1950

Values (%) averaged between 0 m and 2000 m
Integrated from 0000 00 to 0000 00 00 (UTC)
Freq Release started at 0000 00 00 (UTC)

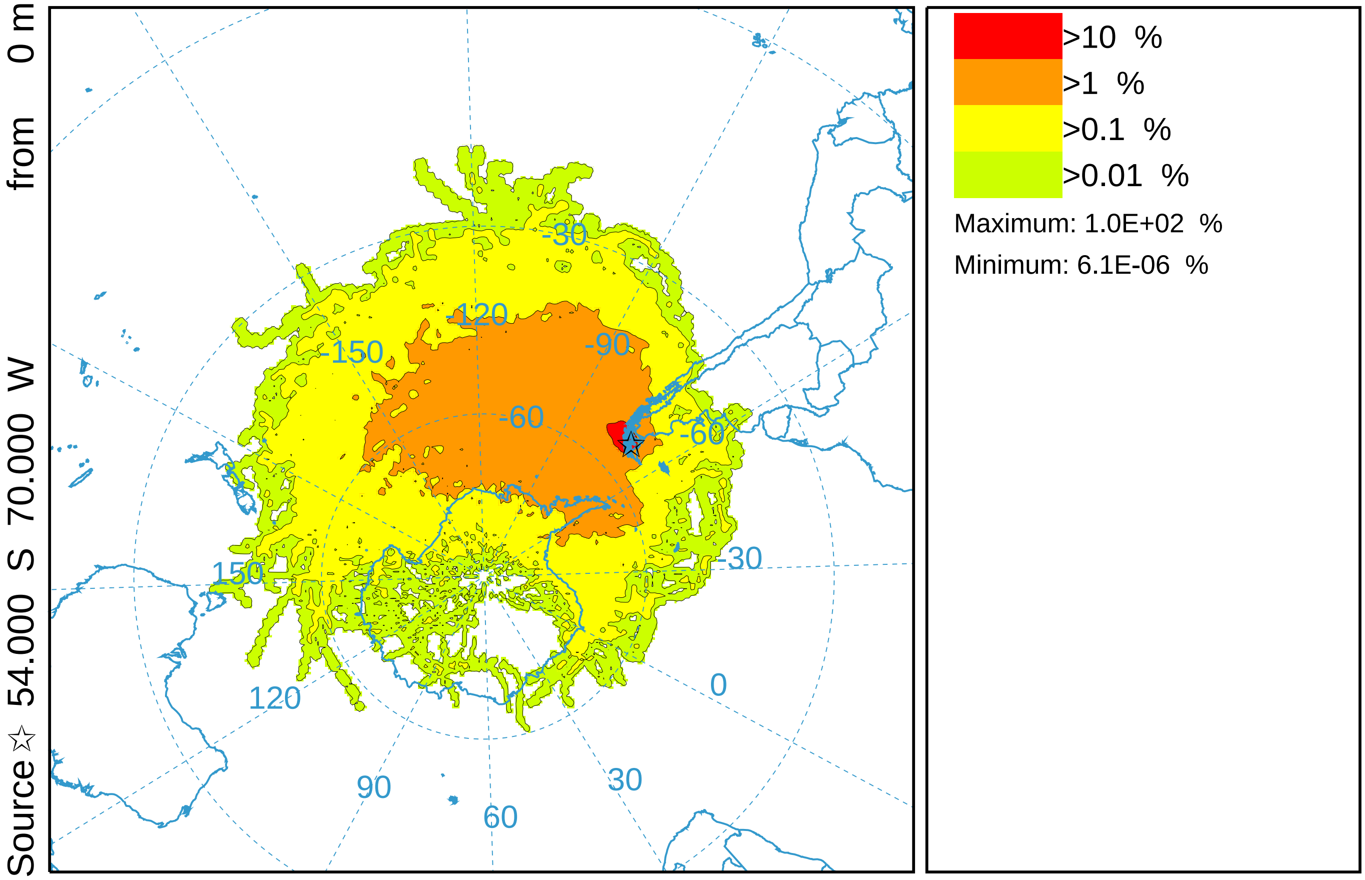

METEOROLOGICAL DATA

Trajectory Frequency Plot year 1951

Values (%) averaged between 0 m and 2000 m
Integrated from 0000 00 to 0000 00 00 (UTC)
Freq Release started at 0000 00 00 (UTC)

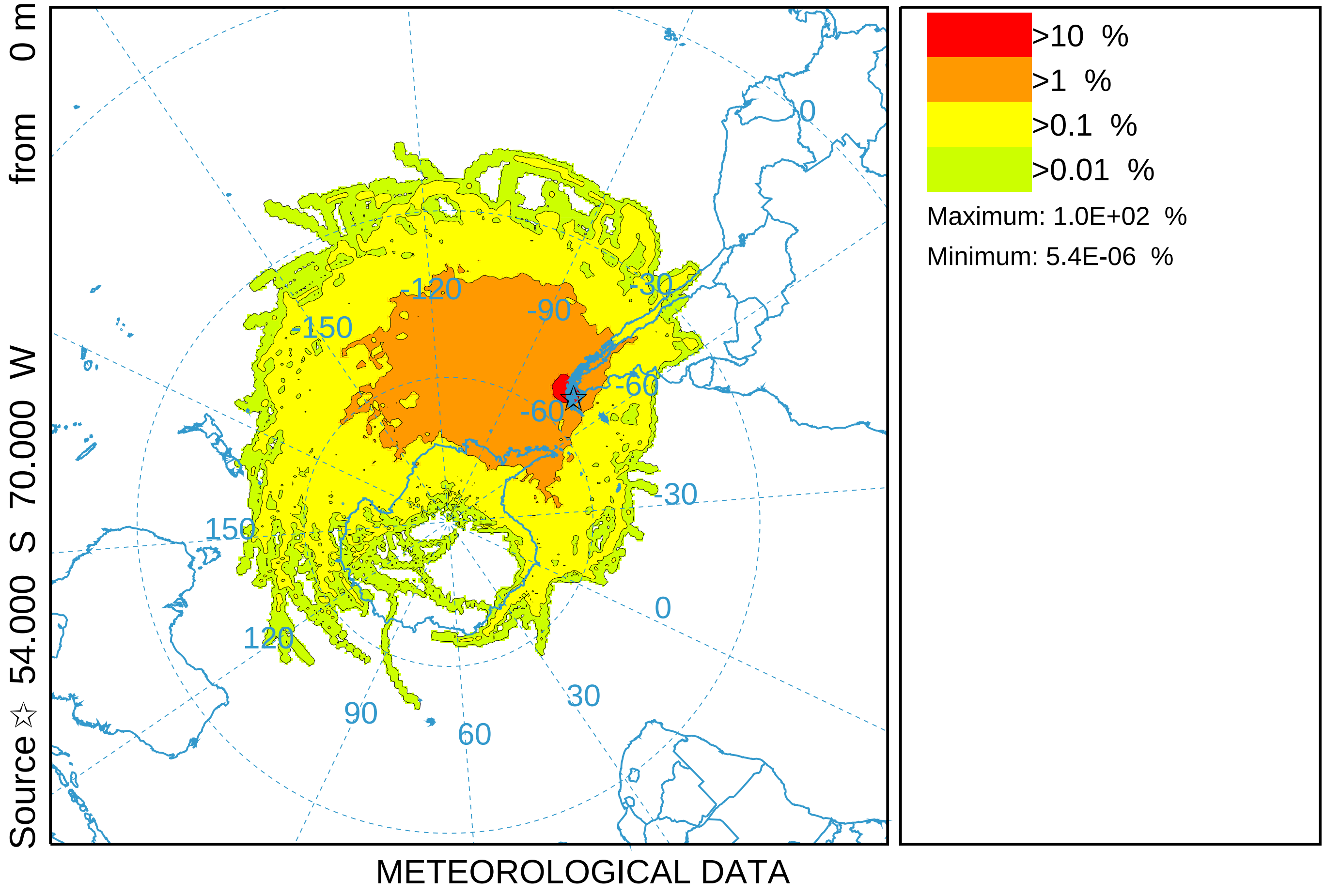

Trajectory Frequency Plot year 1952
Values (%) averaged between 0 m and 2000 m
Integrated from 0000 00 to 0000 00 00 (UTC)
Freq Release started at 0000 00 00 (UTC)

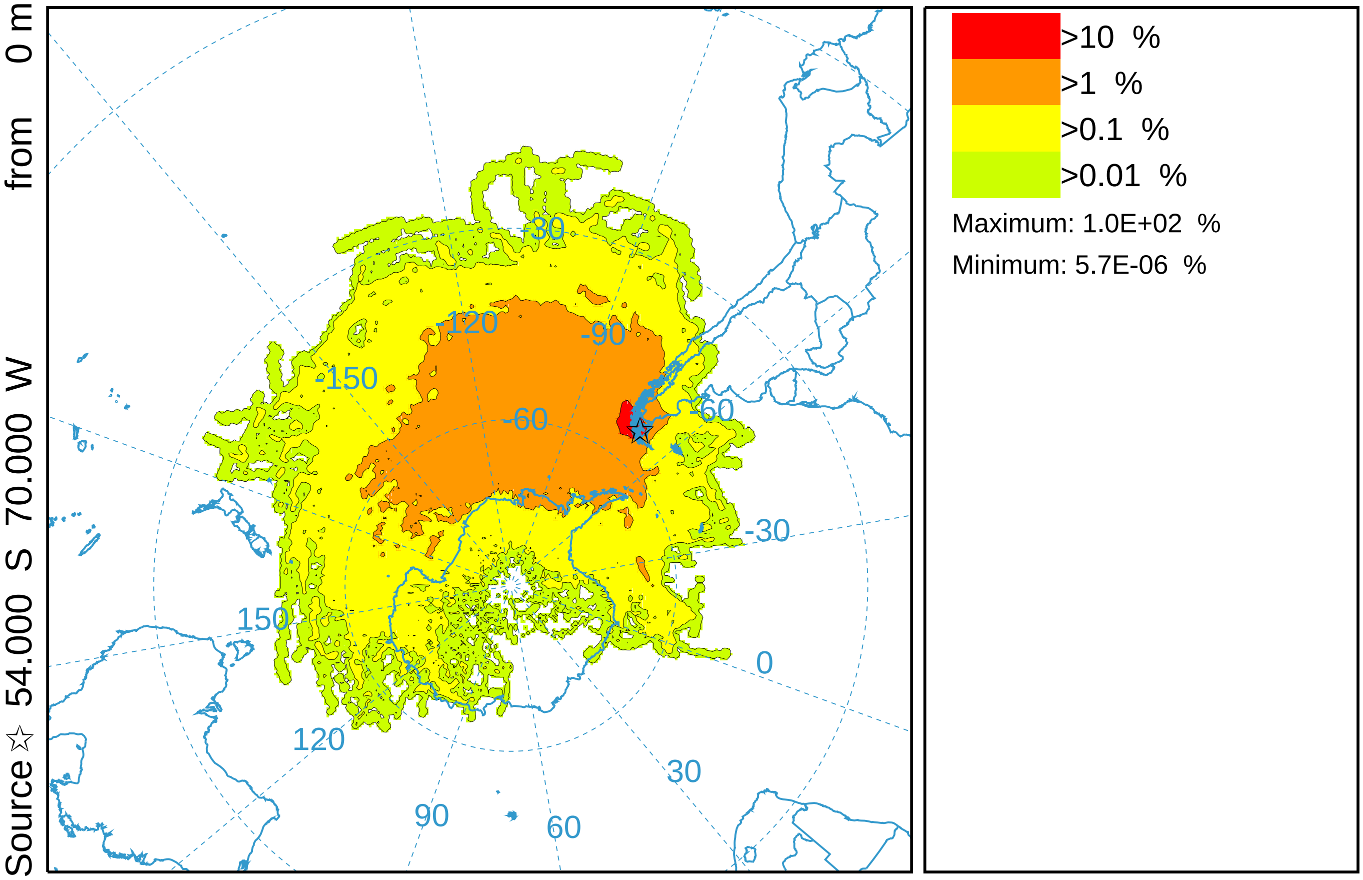

METEOROLOGICAL DATA

Trajectory Frequency Plot year 1953

Values (%) averaged between 0 m and 2000 m

Integrated from 0000 00 to 0000 00 00 (UTC)

Freq Release started at 0000 00 00 (UTC)

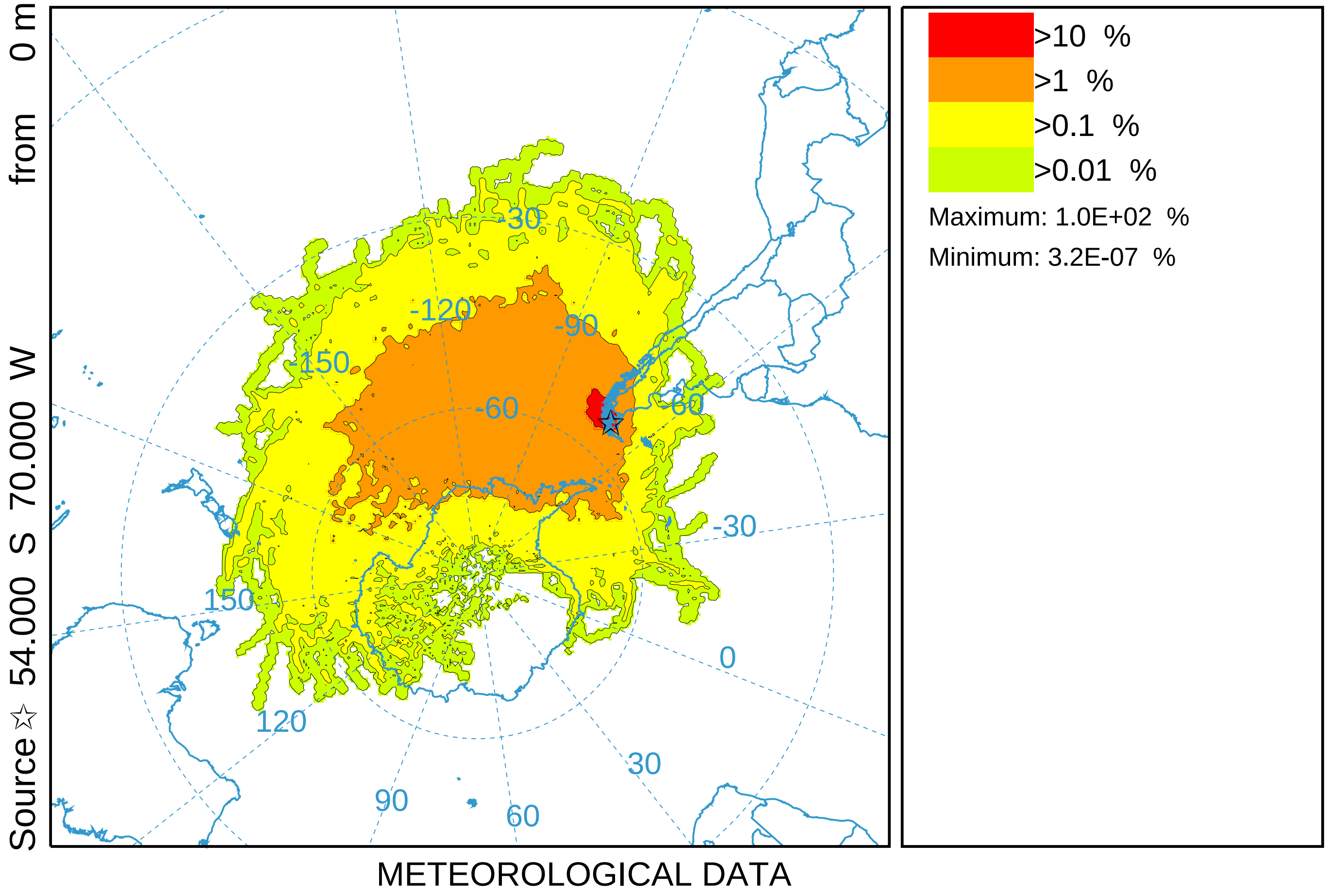

Trajectory Frequency Plot year 1954

Values (%) averaged between 0 m and 2000 m

Integrated from 0000 00 to 0000 00 00 (UTC)

Freq Release started at 0000 00 00 (UTC)

Source ☆ 54.000 S 70.000 W from 0 m

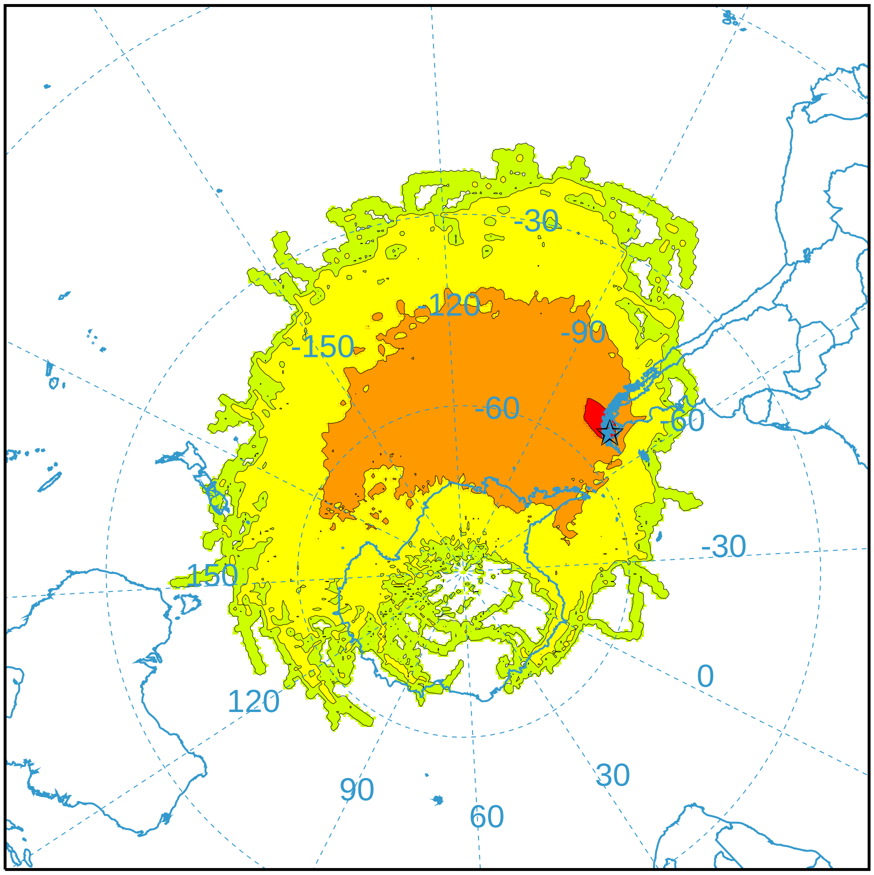

>10 %	
>1 %	
>0.1 %	
>0.01 %	

Maximum: 1.0E+02 %

Minimum: 5.8E-06 %

METEOROLOGICAL DATA

Trajectory Frequency Plot year 1955
Values (%) averaged between 0 m and 2000 m
Integrated from 0000 00 to 0000 00 00 (UTC)
Freq Release started at 0000 00 00 (UTC)

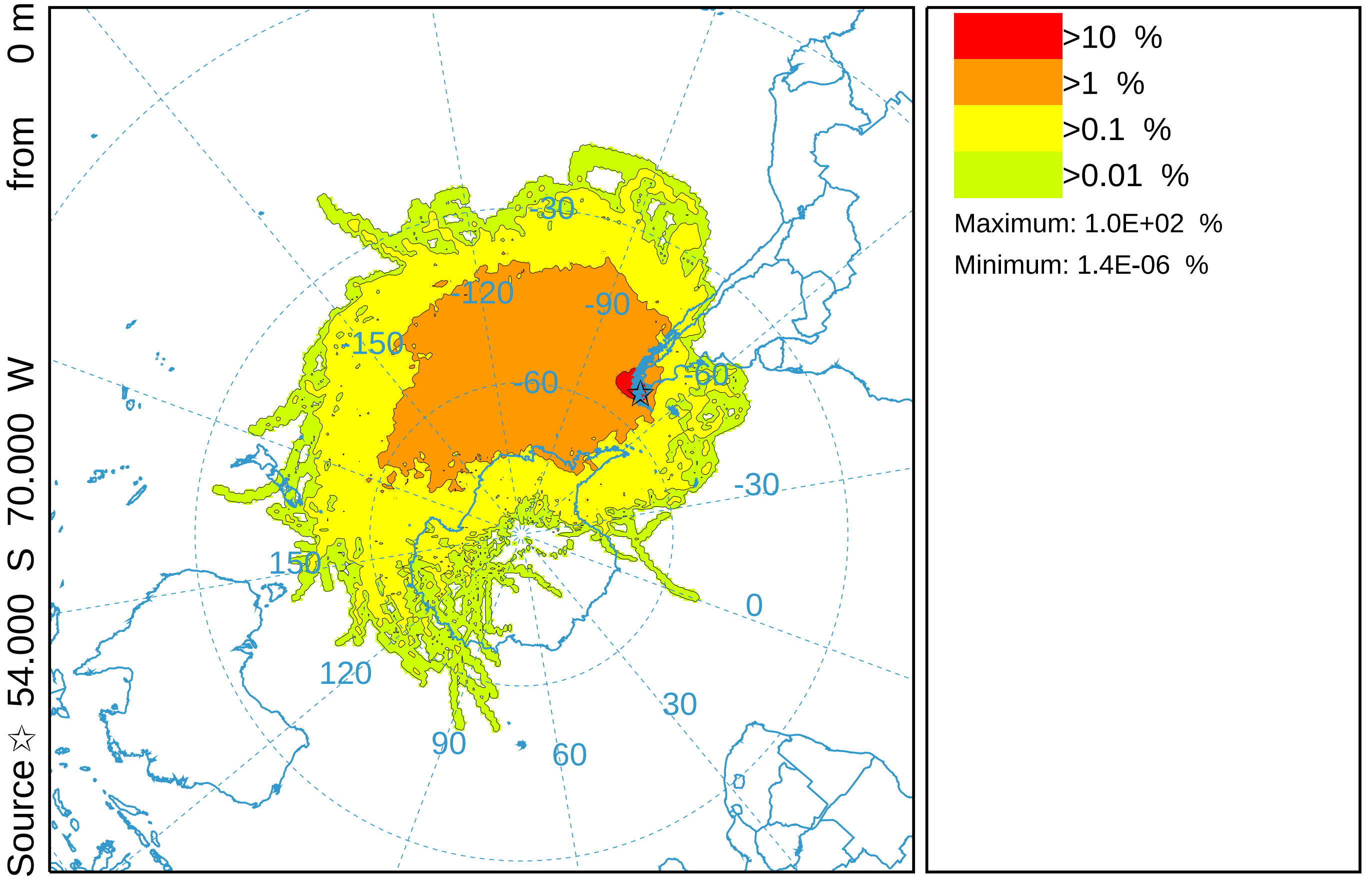

METEOROLOGICAL DATA

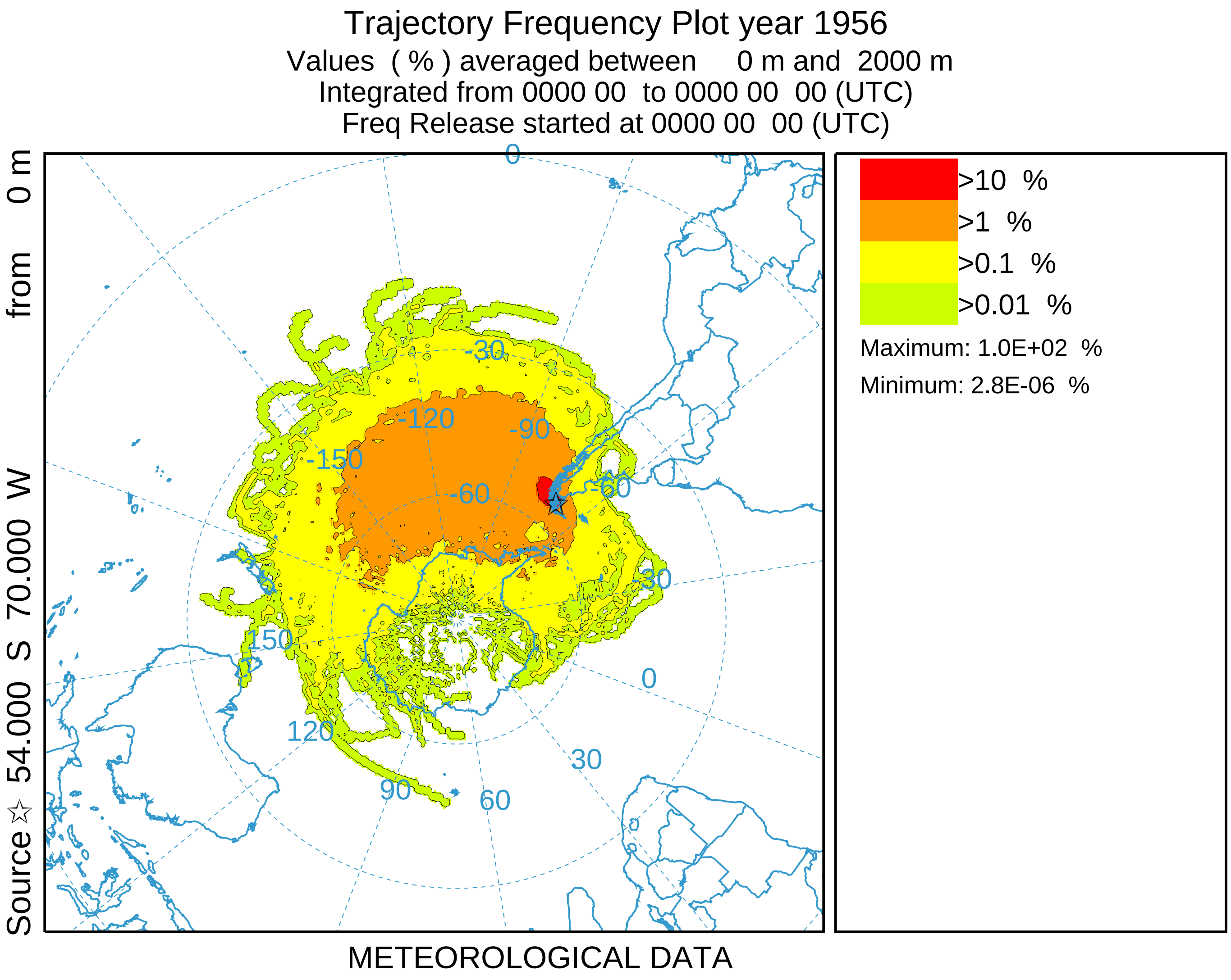
Trajectory Frequency Plot year 1956
Values (%) averaged between 0 m and 2000 m
Integrated from 0000 00 to 0000 00 00 (UTC)
Freq Release started at 0000 00 00 (UTC)
Source ☆ 54.000 S 70.000 W from 0 m
>10 %
>1 %
>0.1 %
>0.01 %
Maximum: 1.0E+02 %
Minimum: 2.8E-06 %
METEOROLOGICAL DATA

Trajectory Frequency Plot year 1957

Values (%) averaged between 0 m and 2000 m

Integrated from 0000 00 to 0000 00 00 (UTC)

Freq Release started at 0000 00 00 (UTC)

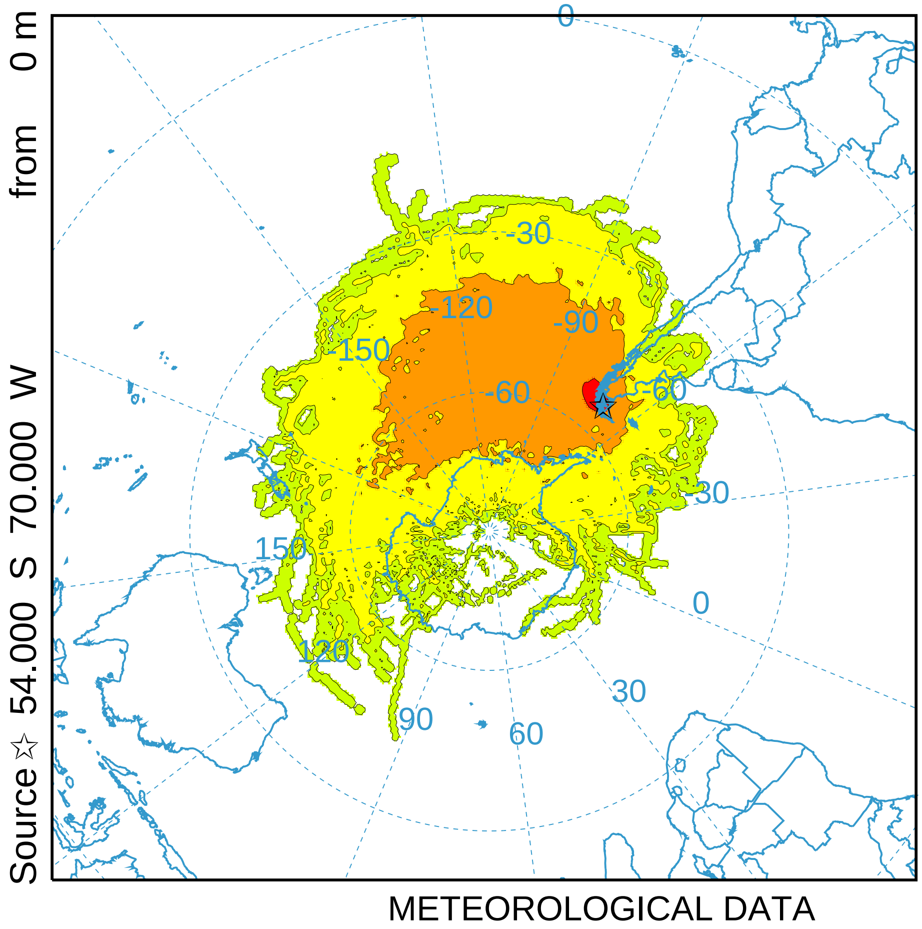

>10 %	
>1 %	
>0.1 %	
>0.01 %	

Maximum: 1.0E+02 %

Minimum: 5.1E-08 %

Trajectory Frequency Plot year 1958

Values (%) averaged between 0 m and 2000 m

Integrated from 0000 00 to 0000 00 00 (UTC)

Freq Release started at 0000 00 00 (UTC)

Source ☆ 54.000 S 70.000 W from 0 m

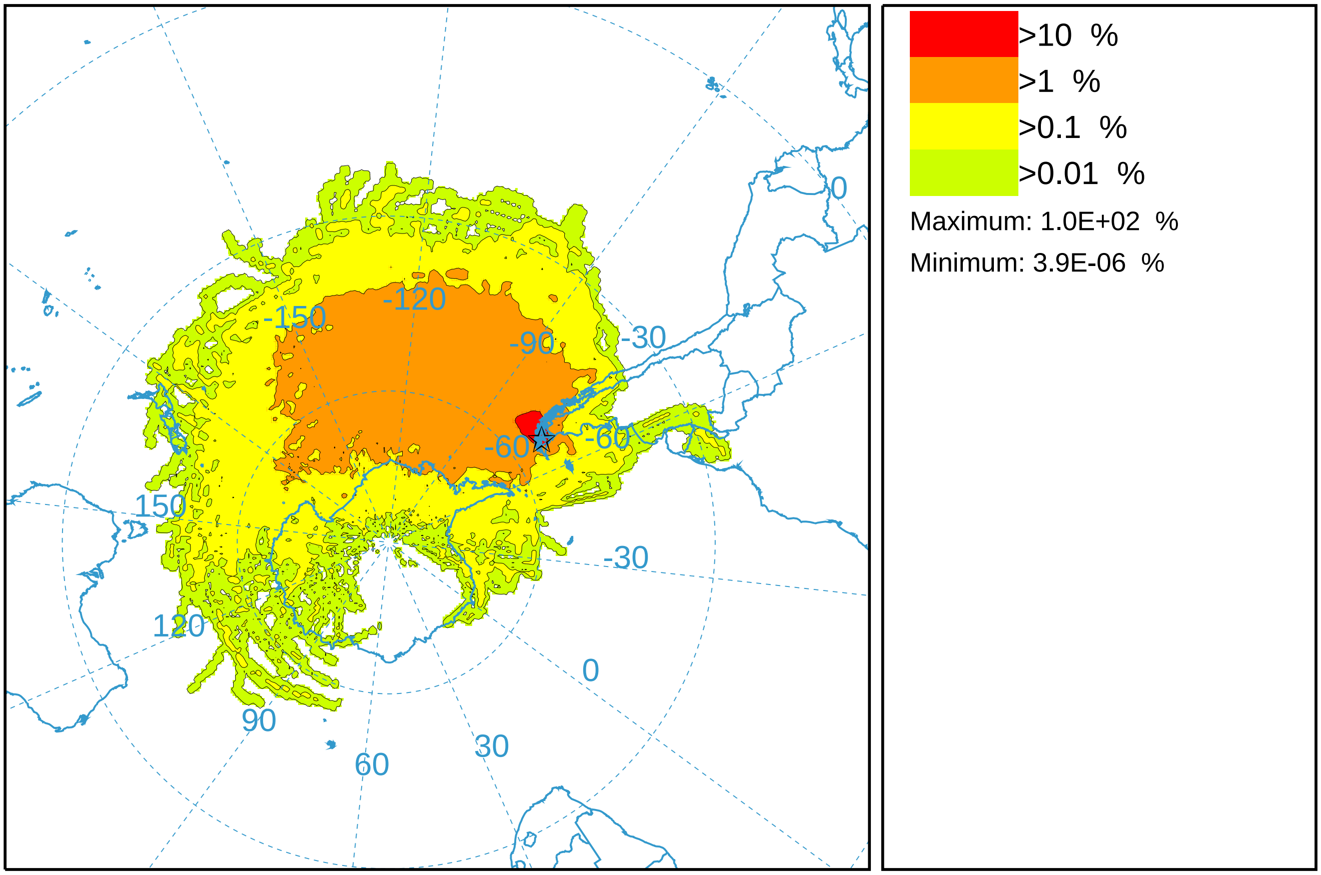

METEOROLOGICAL DATA

Trajectory Frequency Plot year 1959

Values (%) averaged between 0 m and 2000 m

Integrated from 0000 00 to 0000 00 00 (UTC)

Freq Release started at 0000 00 00 (UTC)

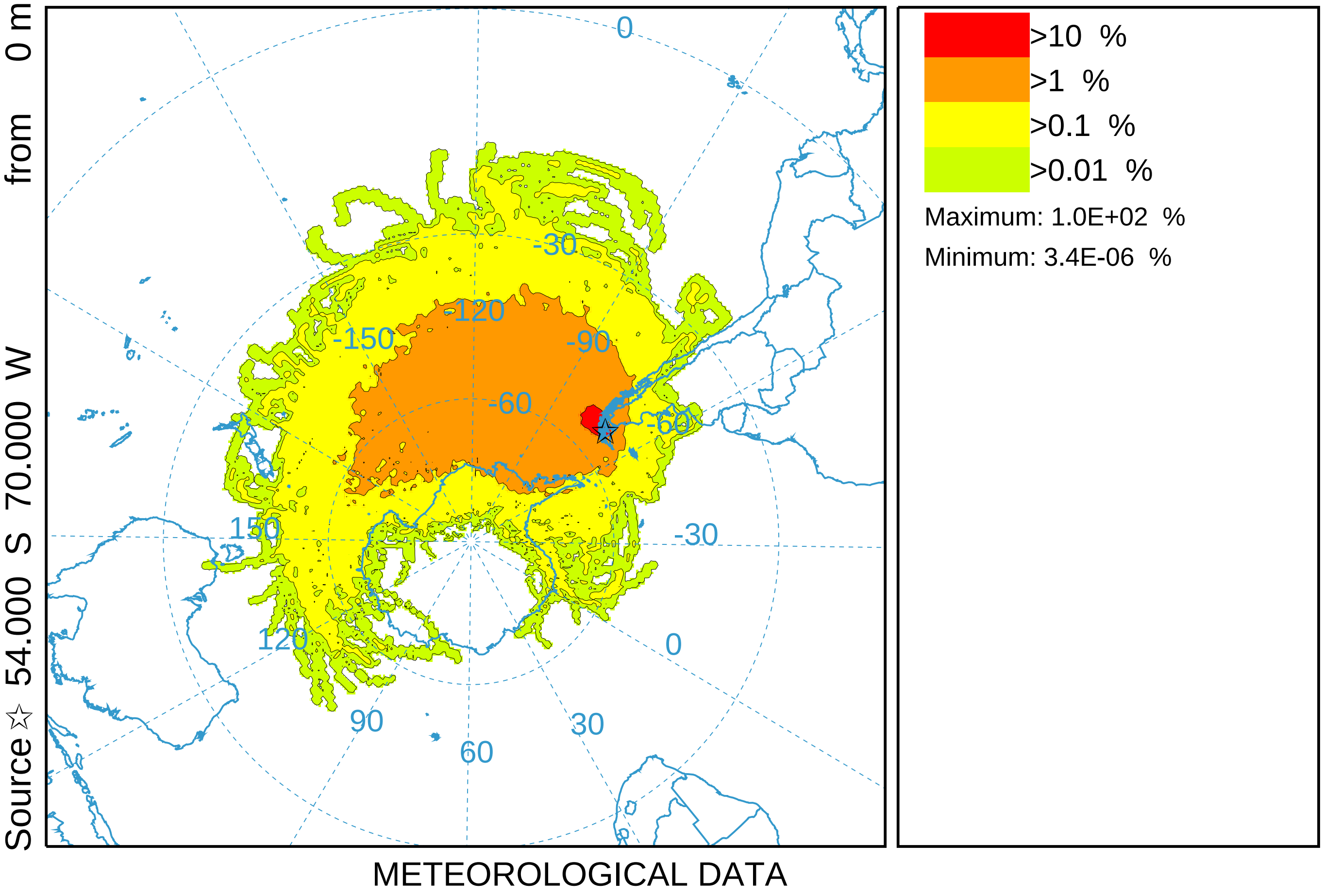

Trajectory Frequency Plot year 1960

Values (%) averaged between 0 m and 2000 m
Integrated from 0000 00 to 0000 00 00 (UTC)
Freq Release started at 0000 00 00 (UTC)

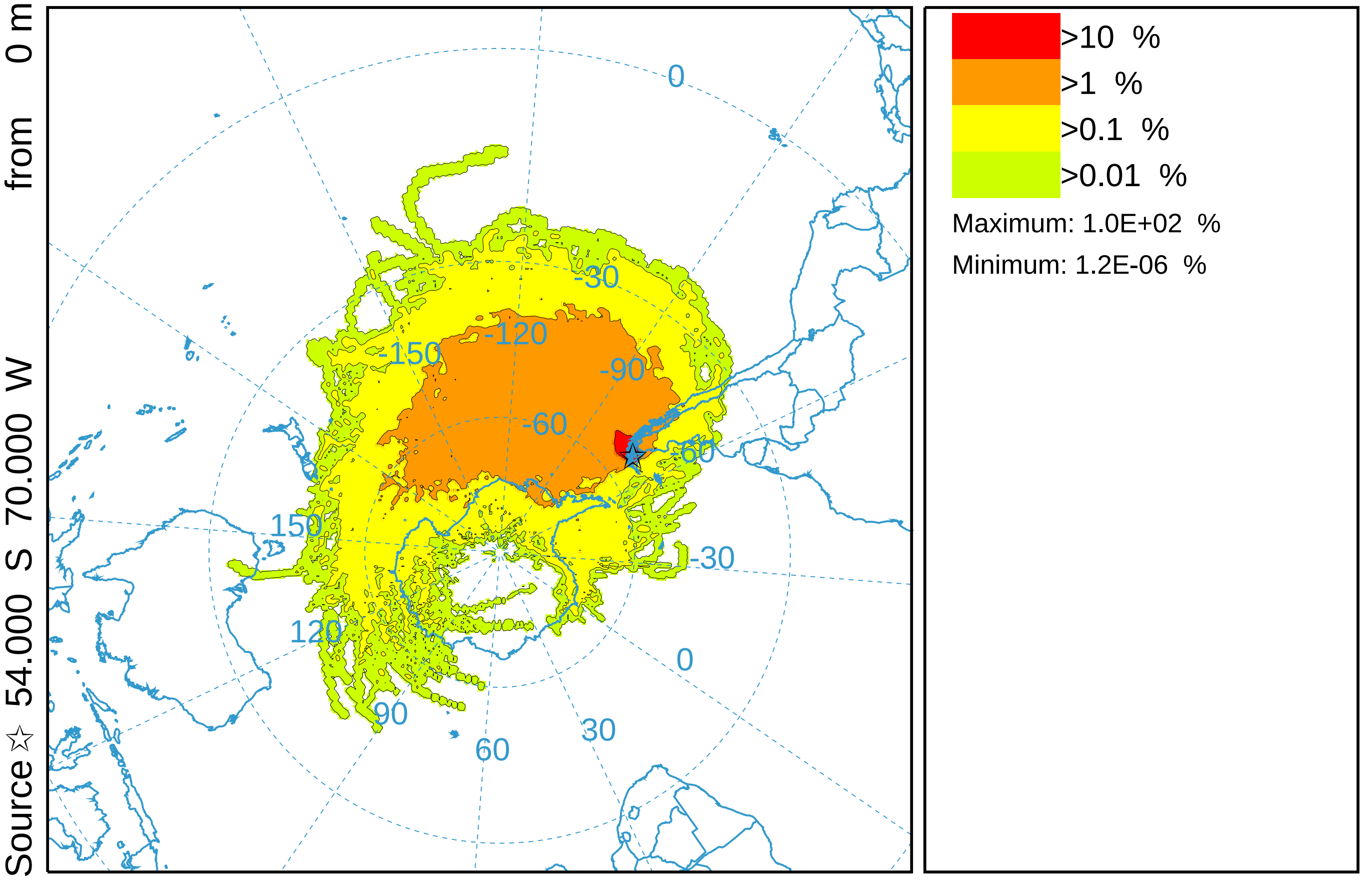

METEOROLOGICAL DATA

Trajectory Frequency Plot year 1961

Values (%) averaged between 0 m and 2000 m

Integrated from 0000 00 to 0000 00 00 (UTC)

Freq Release started at 0000 00 00 (UTC)

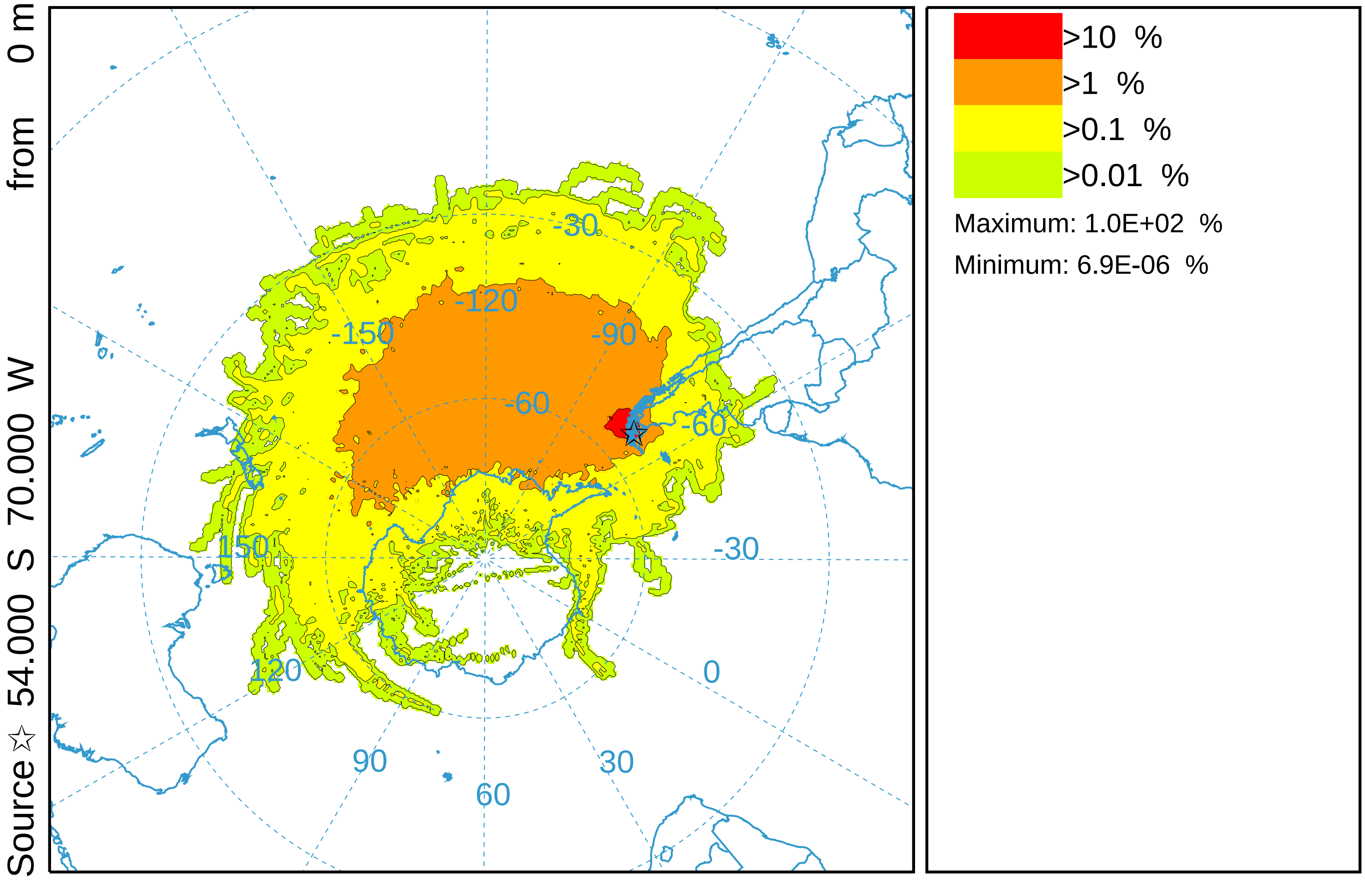

METEOROLOGICAL DATA

Trajectory Frequency Plot year 1962

Values (%) averaged between 0 m and 2000 m
Integrated from 0000 00 to 0000 00 00 (UTC)
Freq Release started at 0000 00 00 (UTC)

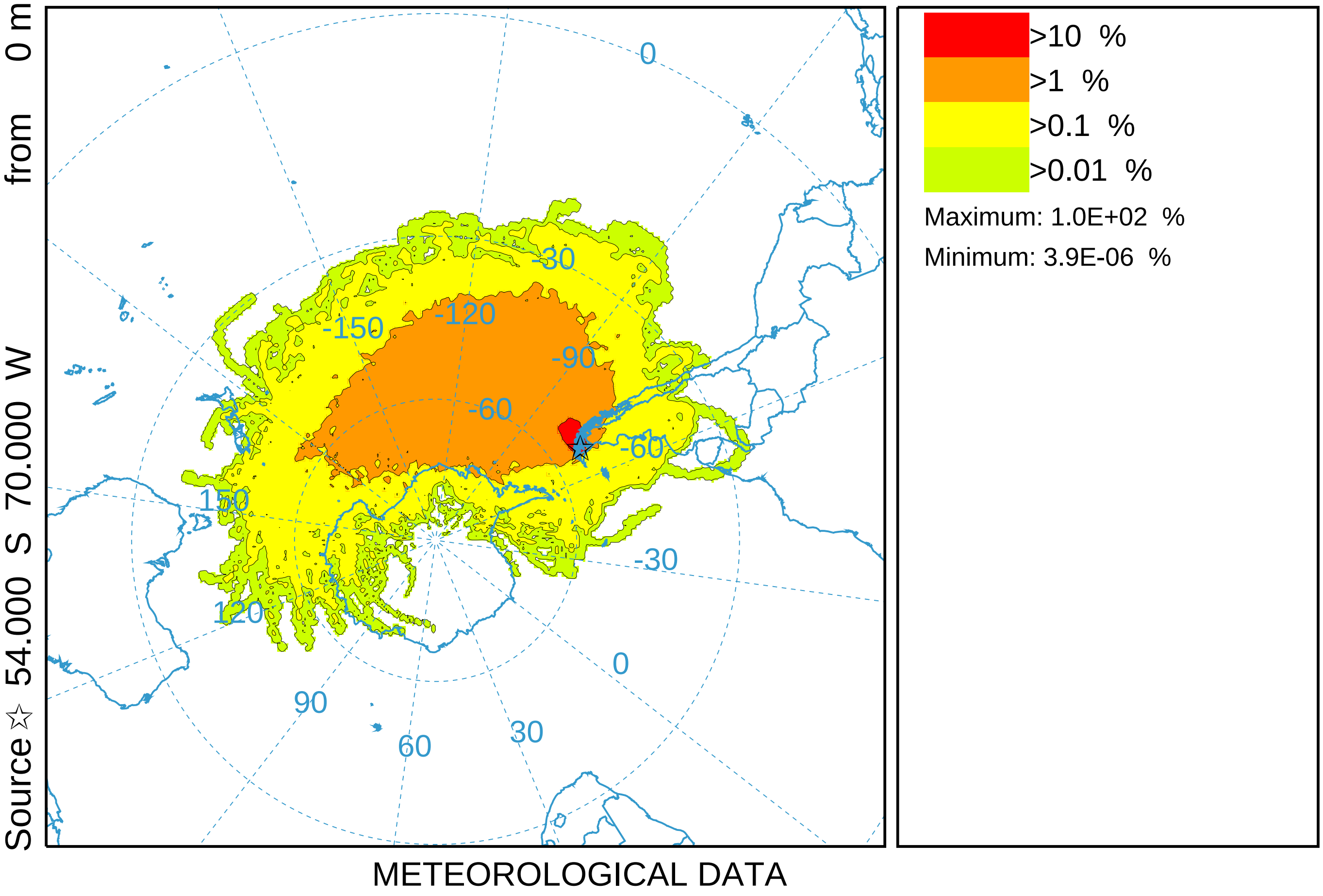

Trajectory Frequency Plot year 1963

Values (%) averaged between 0 m and 2000 m

Integrated from 0000 00 to 0000 00 00 (UTC)

Freq Release started at 0000 00 00 (UTC)

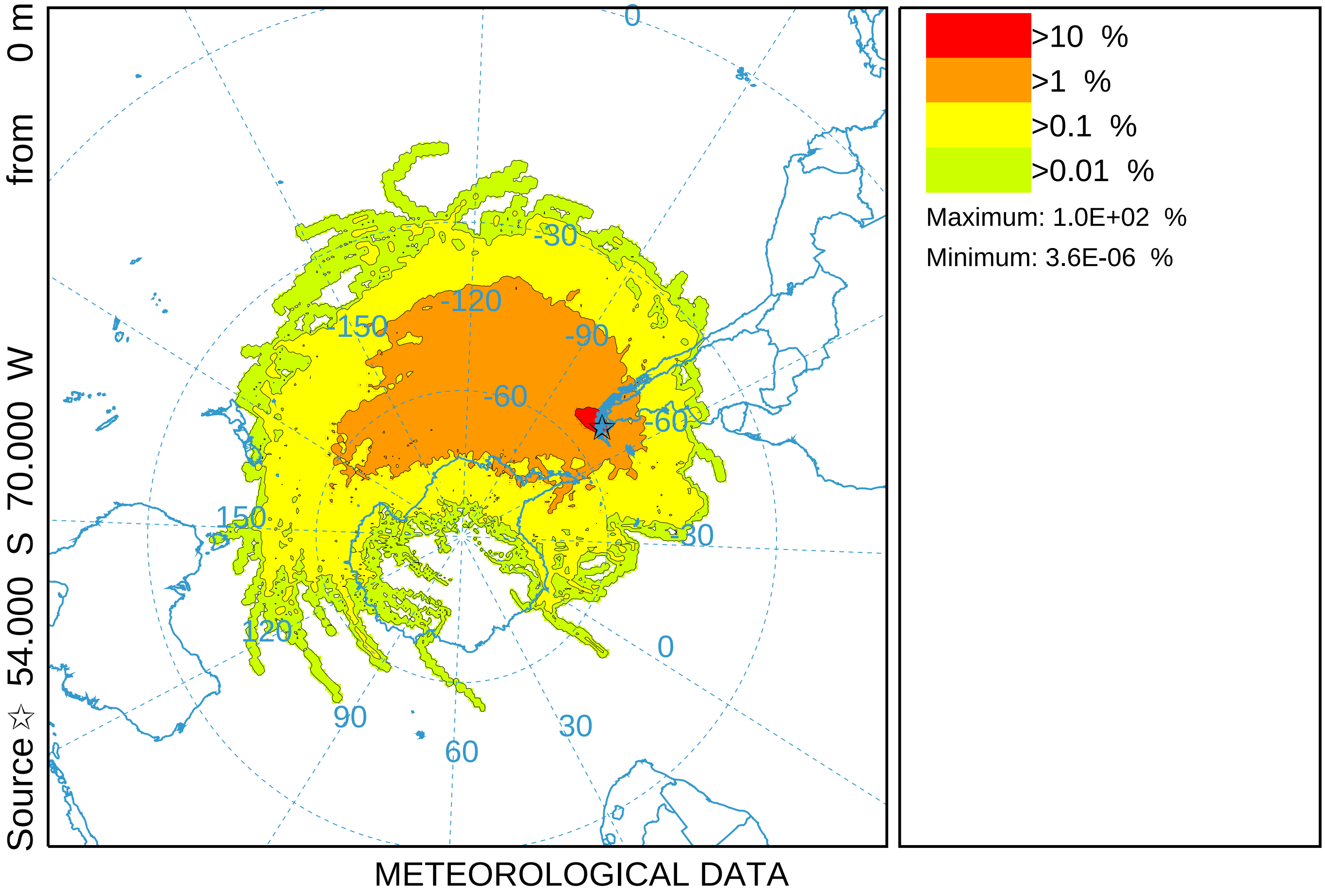

Trajectory Frequency Plot year 1964

Values (%) averaged between 0 m and 2000 m
Integrated from 0000 00 to 0000 00 00 (UTC)
Freq Release started at 0000 00 00 (UTC)

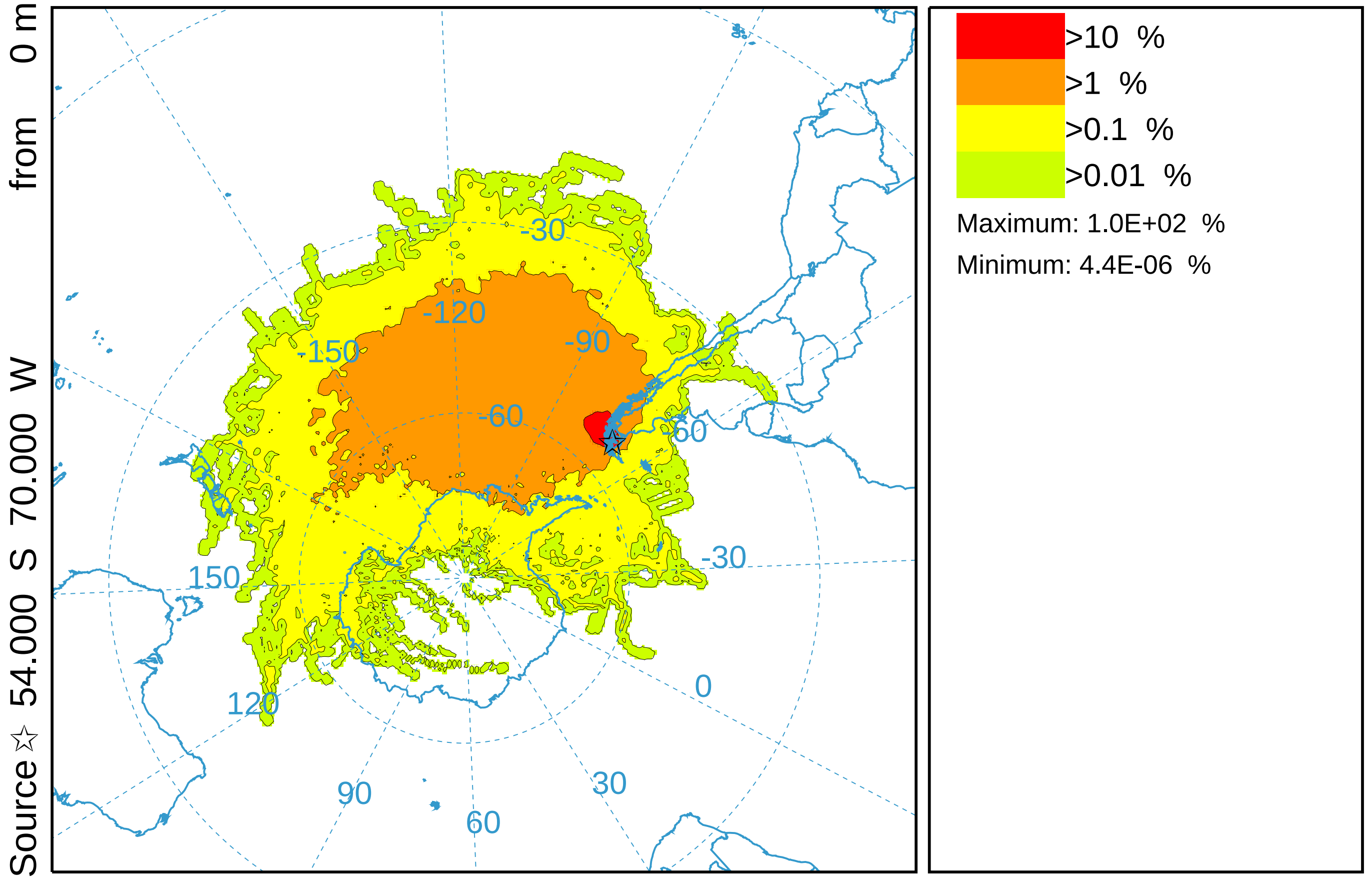

METEOROLOGICAL DATA

Trajectory Frequency Plot year 1965
Values (%) averaged between 0 m and 2000 m
Integrated from 0000 00 to 0000 00 00 (UTC)
Freq Release started at 0000 00 00 (UTC)

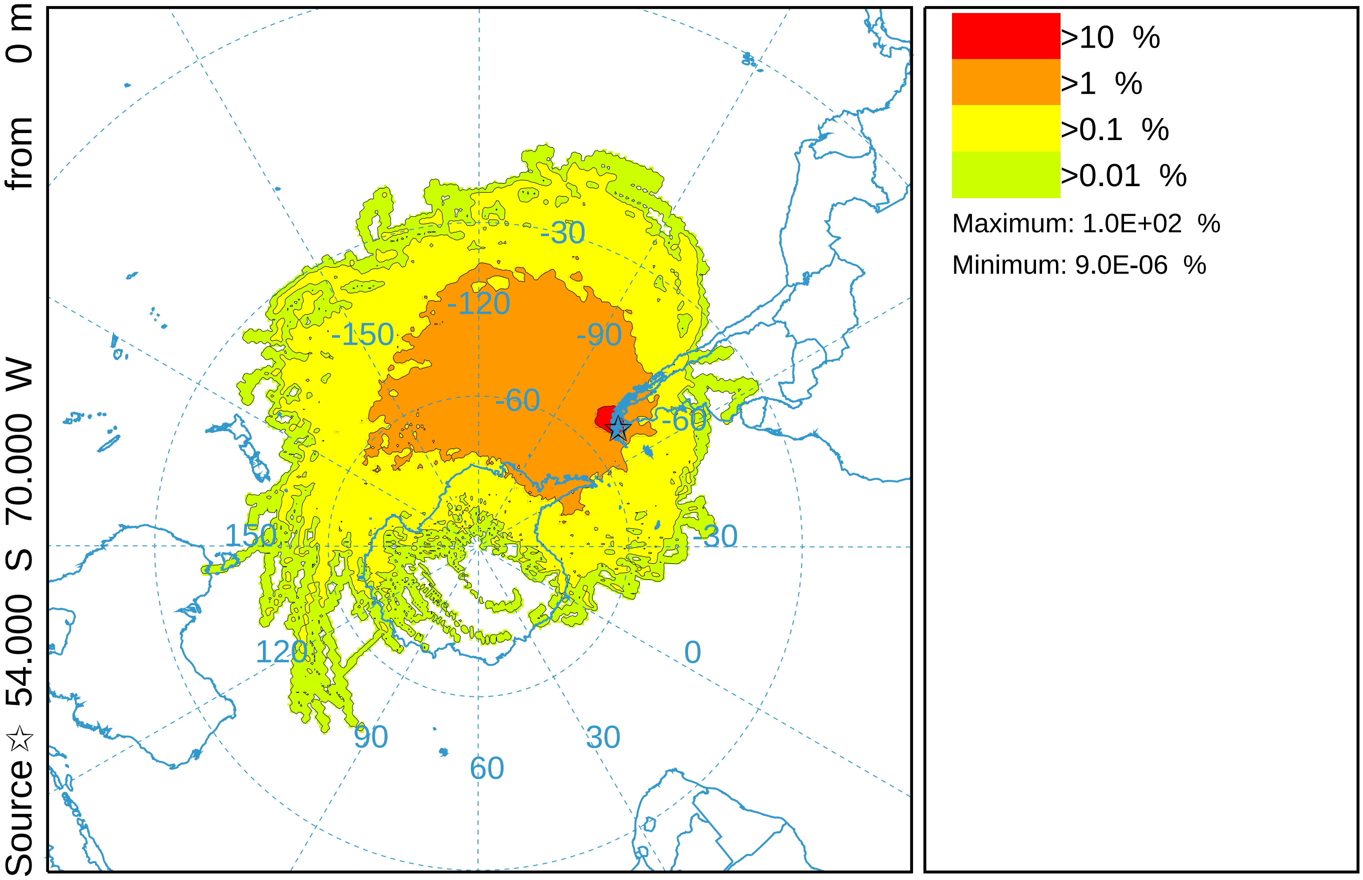

METEOROLOGICAL DATA

Trajectory Frequency Plot year 1966

Values (%) averaged between 0 m and 2000 m

Integrated from 0000 00 to 0000 00 00 (UTC)

Freq Release started at 0000 00 00 (UTC)

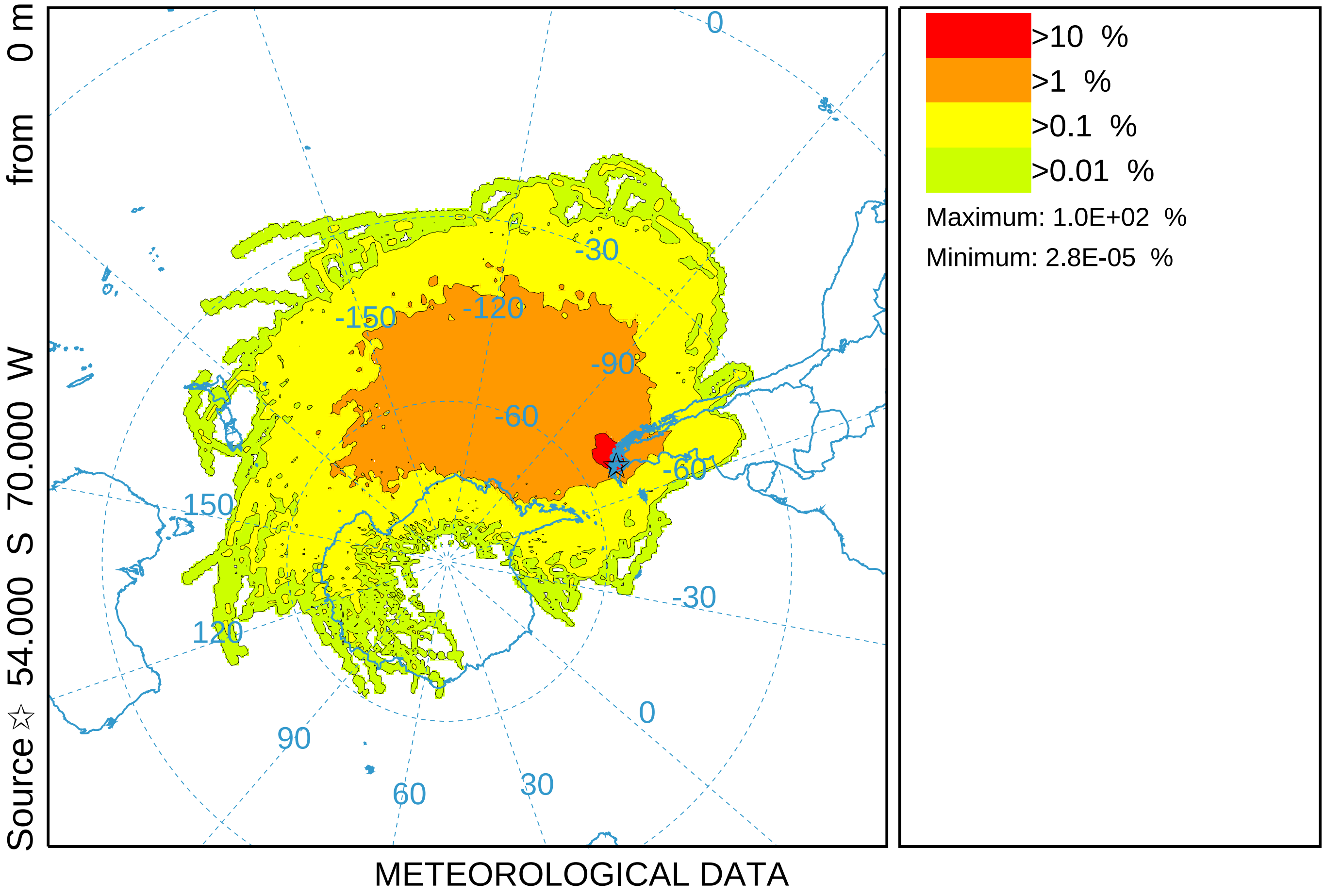

Trajectory Frequency Plot year 1967

Values (%) averaged between 0 m and 2000 m

Integrated from 0000 00 to 0000 00 00 (UTC)

Freq Release started at 0000 00 00 (UTC)

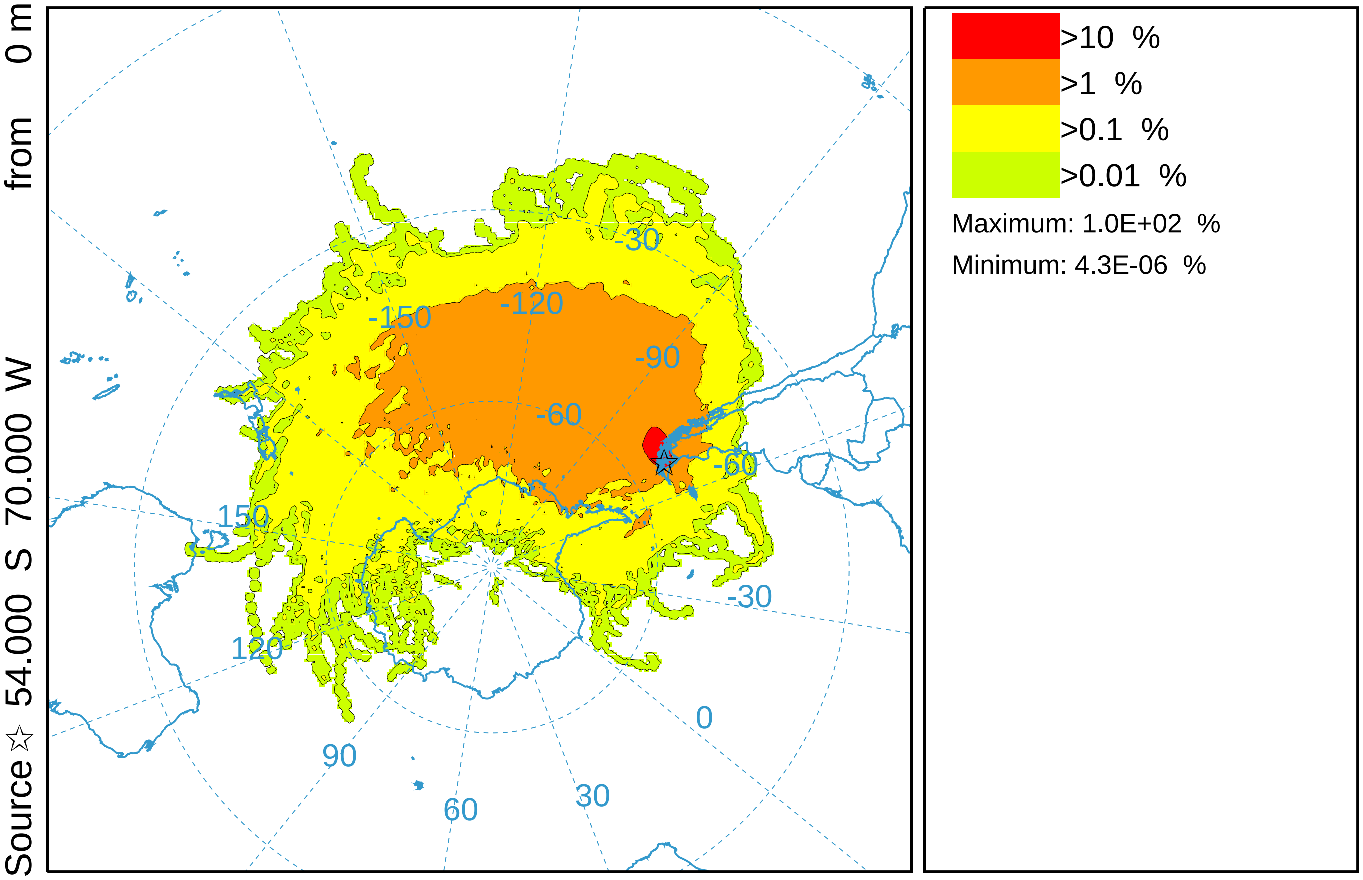

METEOROLOGICAL DATA

Trajectory Frequency Plot year 1968

Values (%) averaged between 0 m and 2000 m
Integrated from 0000 00 to 0000 00 00 (UTC)
Freq Release started at 0000 00 00 (UTC)

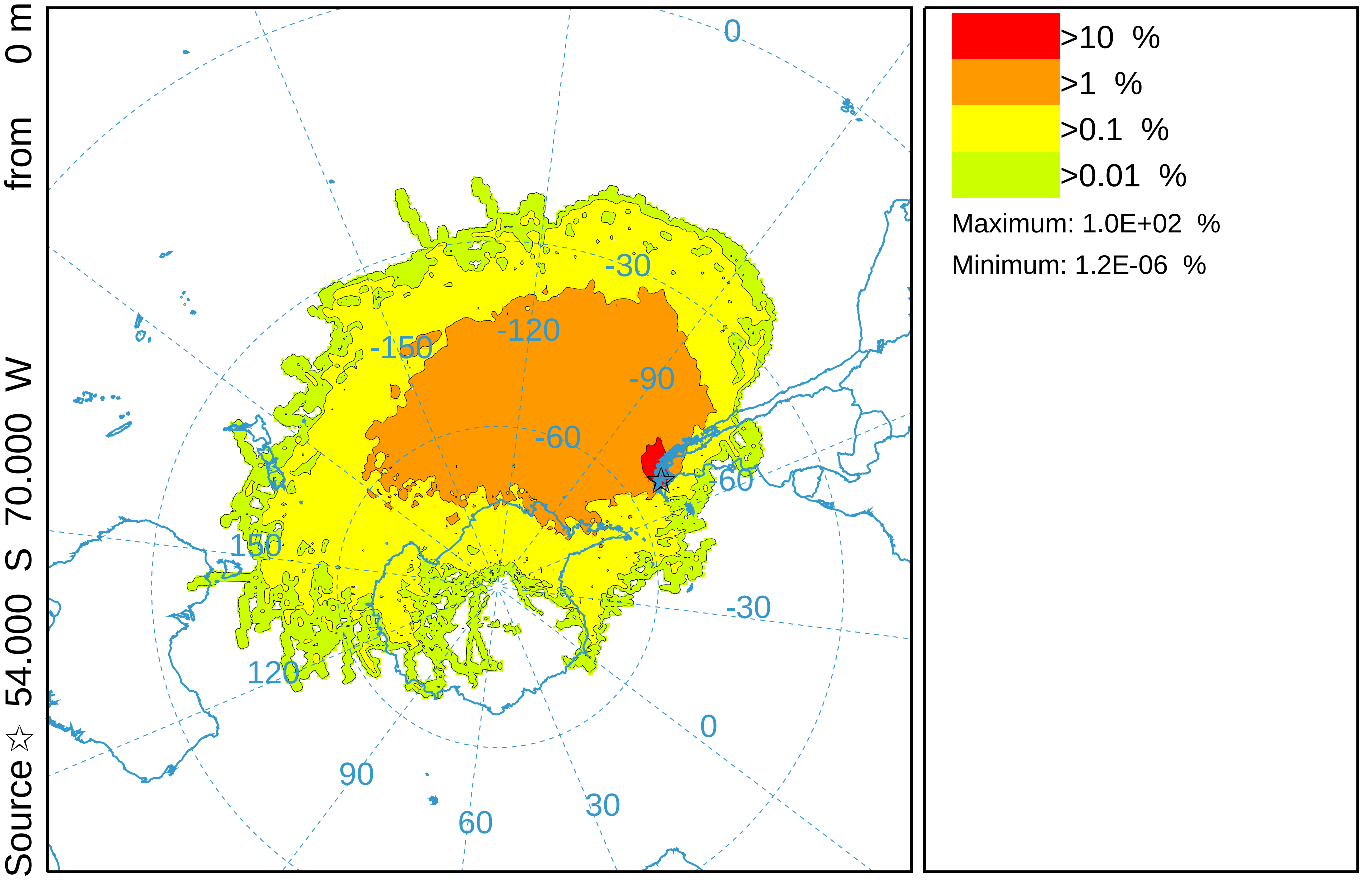

METEOROLOGICAL DATA

Trajectory Frequency Plot year 1969
Values (%) averaged between 0 m and 2000 m
Integrated from 0000 00 to 0000 00 00 (UTC)
Freq Release started at 0000 00 00 (UTC)

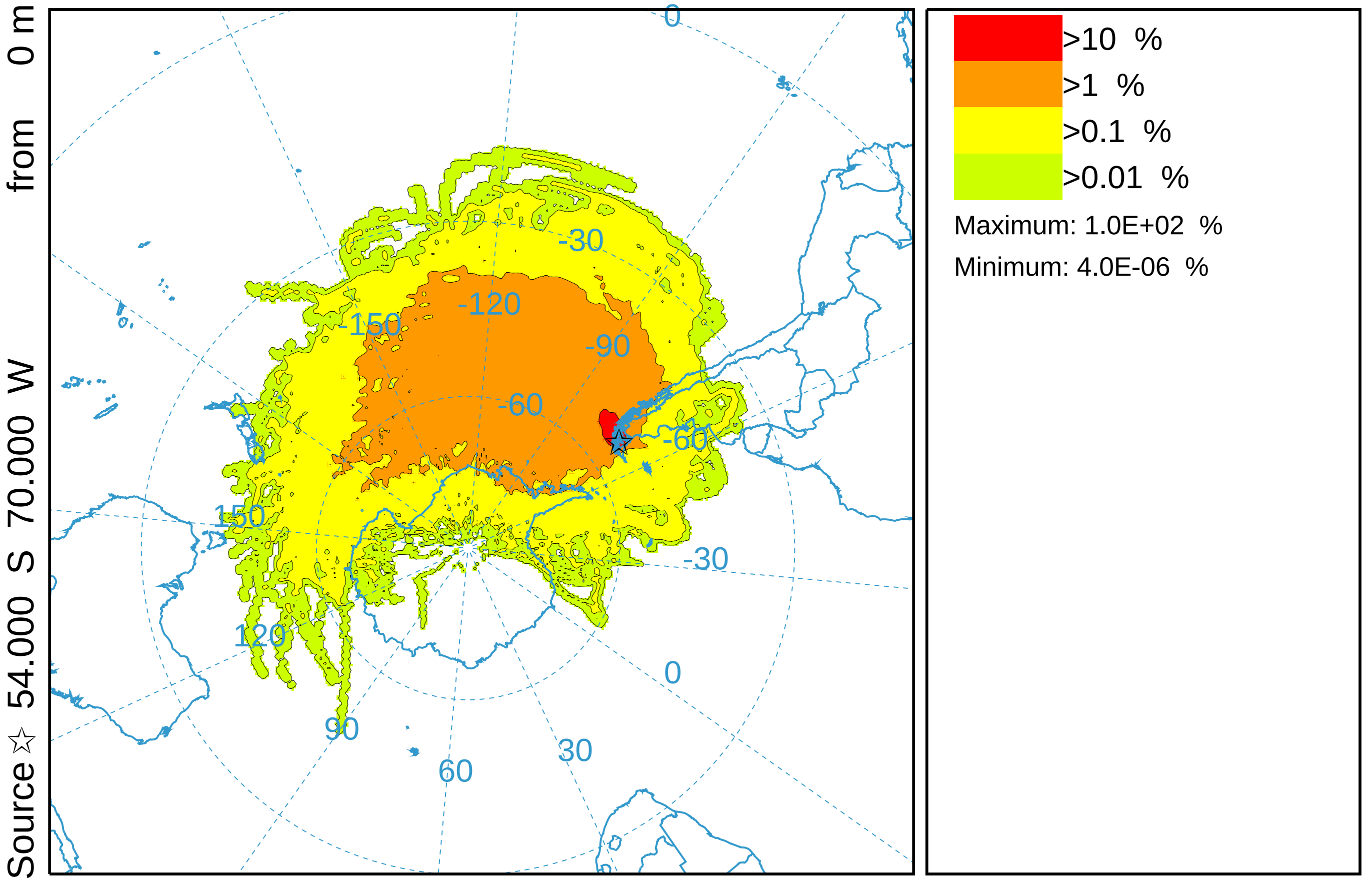

METEOROLOGICAL DATA

Trajectory Frequency Plot year 1970

Values (%) averaged between 0 m and 2000 m

Integrated from 0000 00 to 0000 00 00 (UTC)

Freq Release started at 0000 00 00 (UTC)

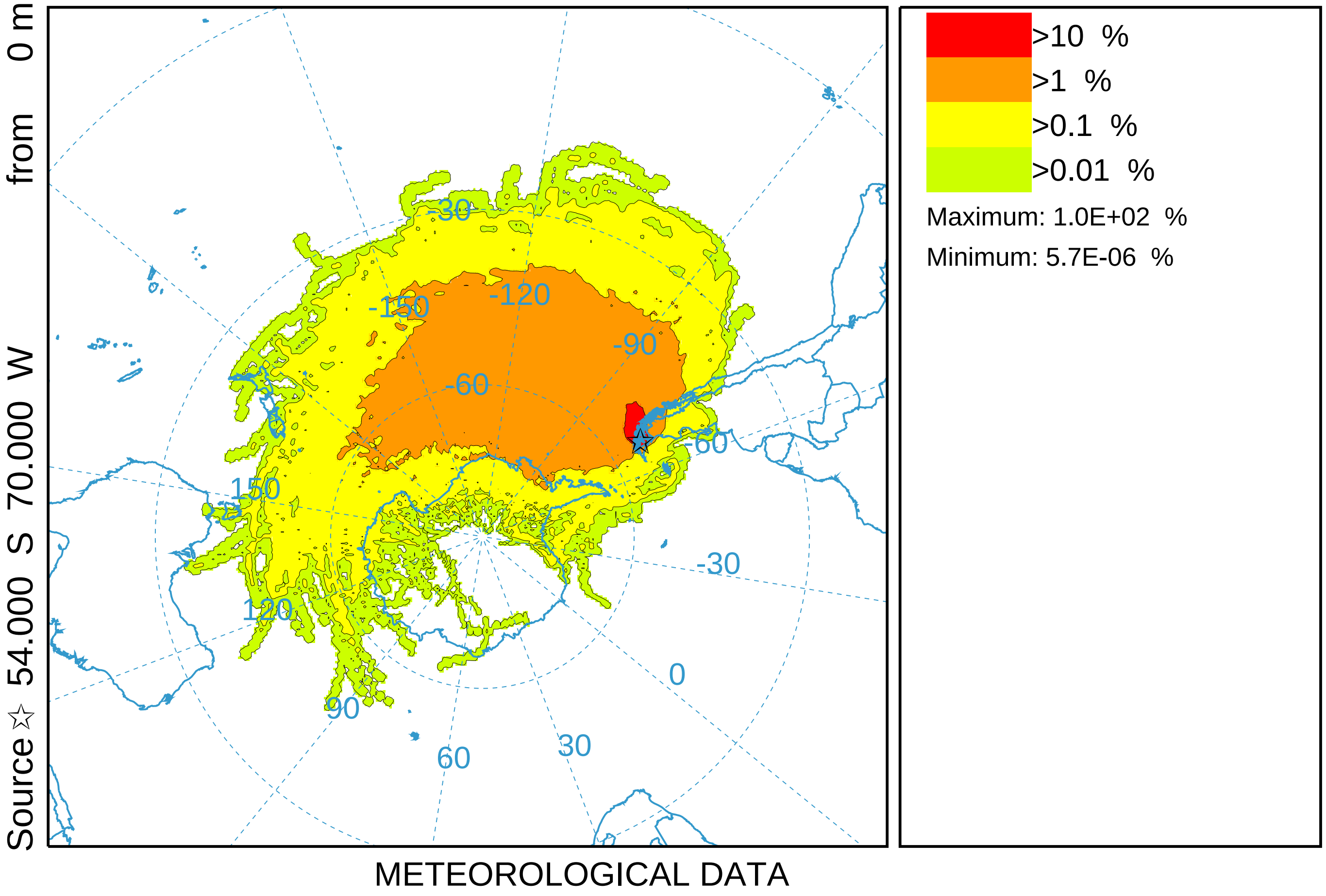

Trajectory Frequency Plot year 1971

Values (%) averaged between 0 m and 2000 m

Integrated from 0000 00 to 0000 00 00 (UTC)

Freq Release started at 0000 00 00 (UTC)

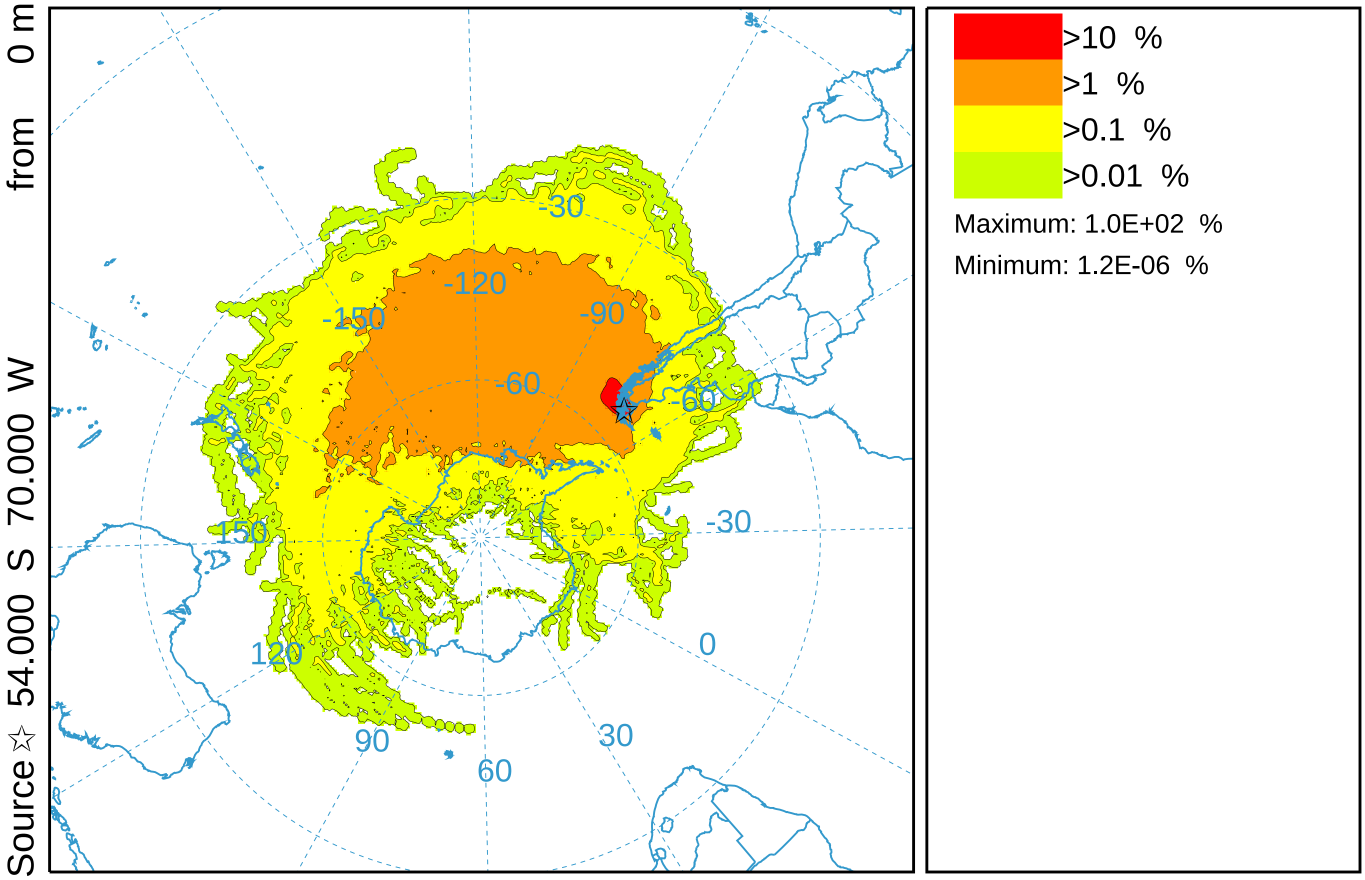

METEOROLOGICAL DATA

Trajectory Frequency Plot year 1972
Values (%) averaged between 0 m and 2000 m
Integrated from 0000 00 to 0000 00 00 (UTC)
Freq Release started at 0000 00 00 (UTC)

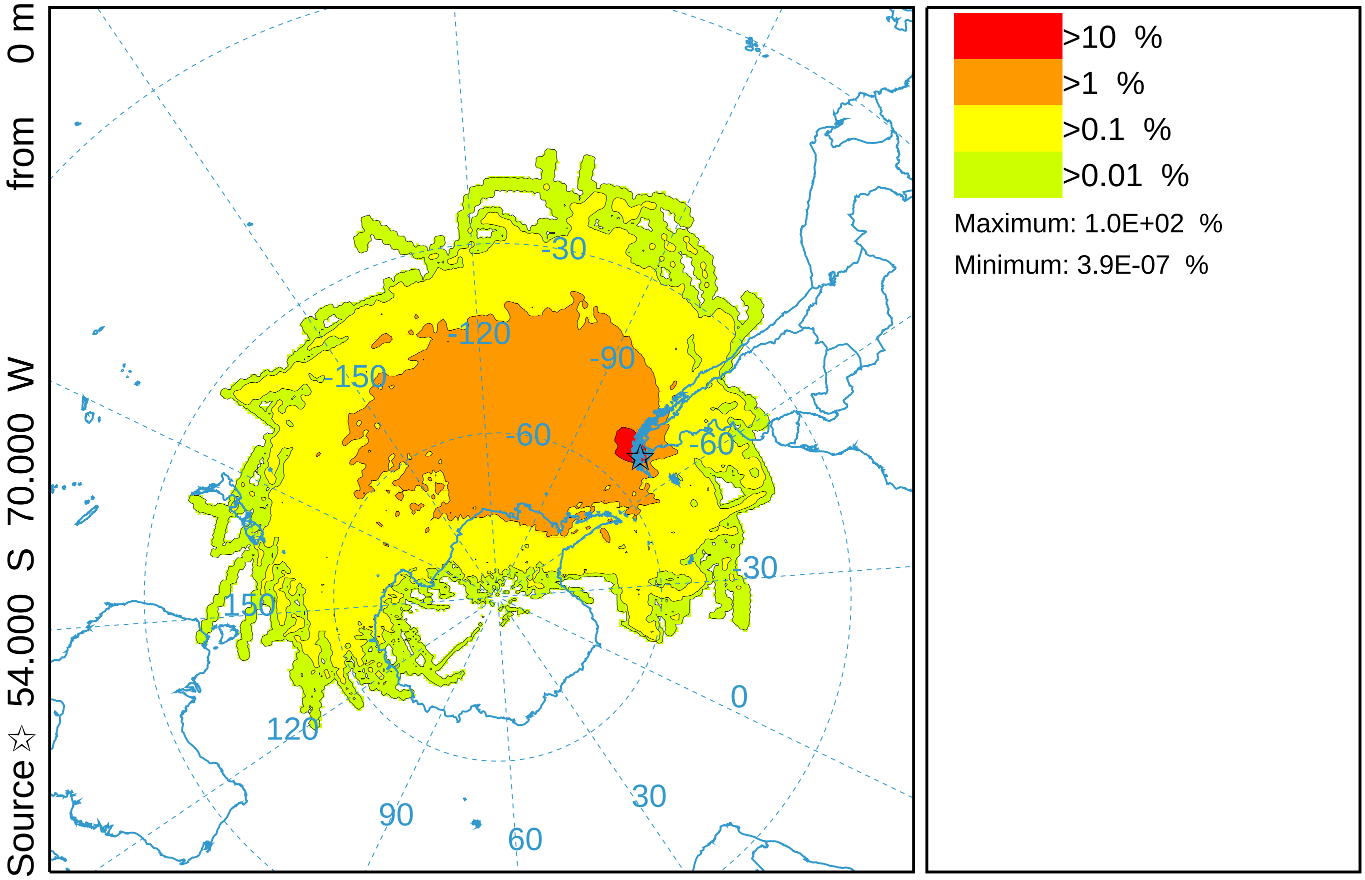

METEOROLOGICAL DATA

Trajectory Frequency Plot year 1973
Values (%) averaged between 0 m and 2000 m
Integrated from 0000 00 to 0000 00 00 (UTC)
Freq Release started at 0000 00 00 (UTC)

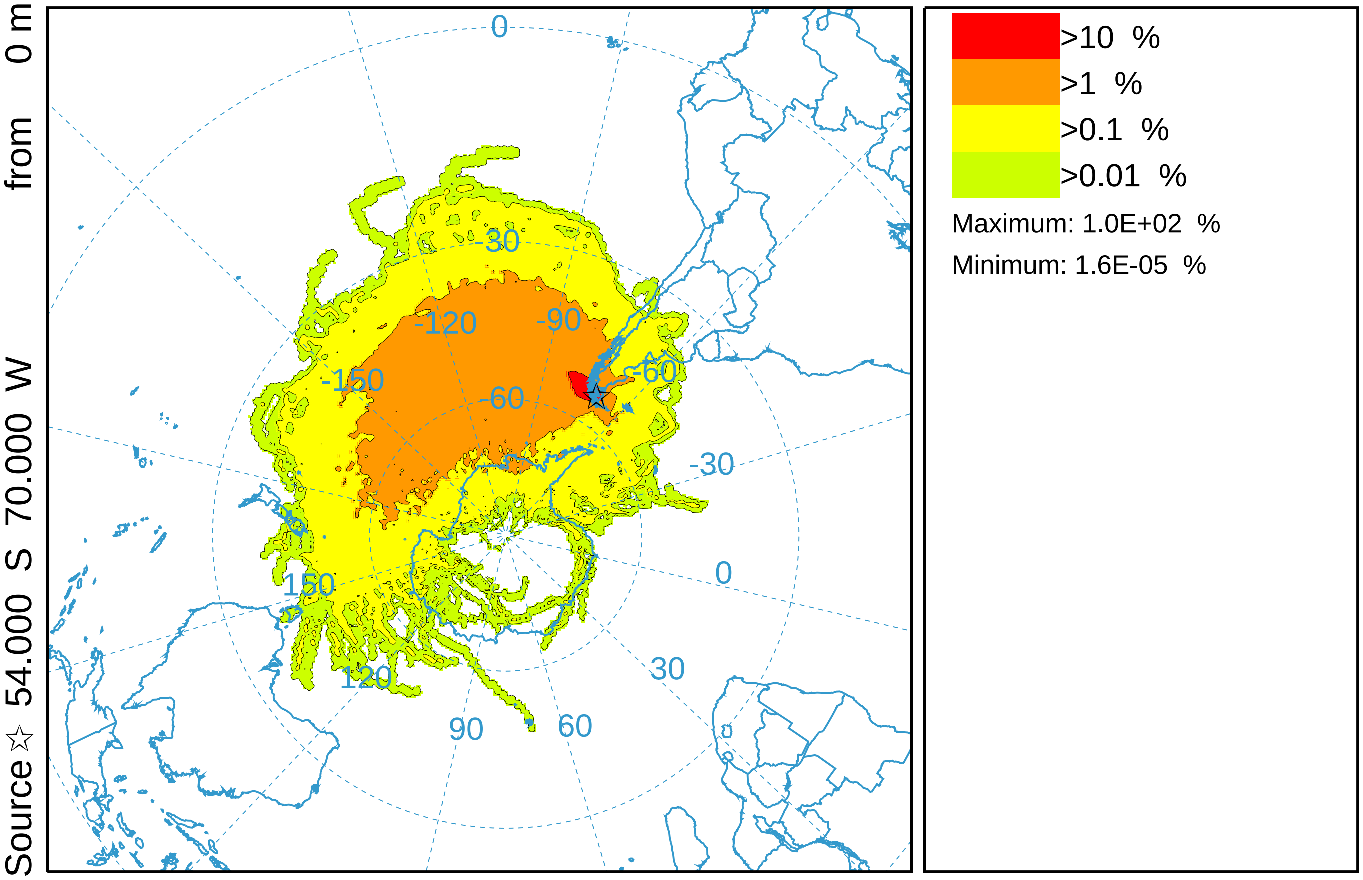

METEOROLOGICAL DATA

Trajectory Frequency Plot year 1974
Values (%) averaged between 0 m and 2000 m
Integrated from 0000 00 to 0000 00 00 (UTC)
Freq Release started at 0000 00 00 (UTC)

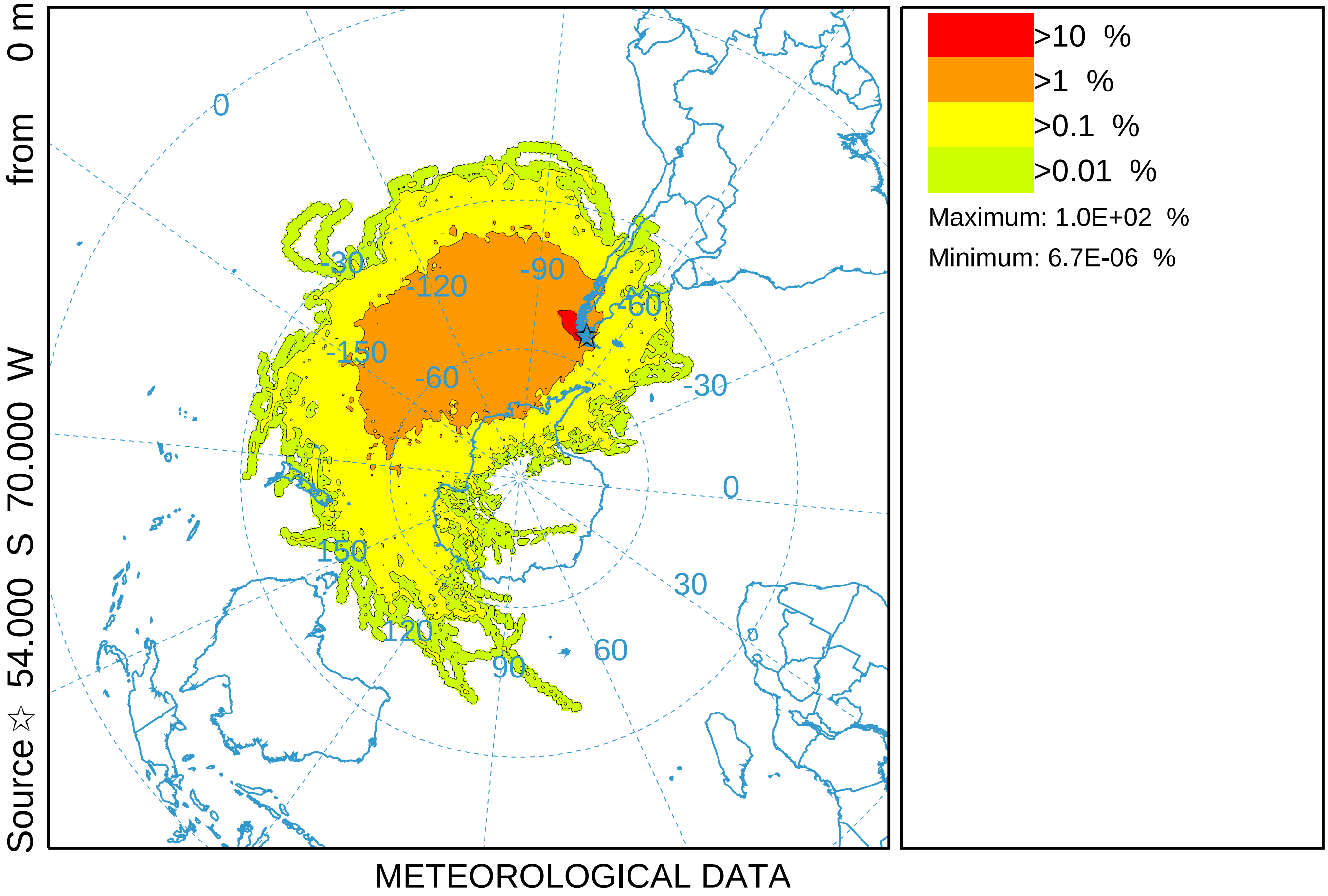

Trajectory Frequency Plot year 1975
Values (%) averaged between 0 m and 2000 m
Integrated from 0000 00 to 0000 00 00 (UTC)
Freq Release started at 0000 00 00 (UTC)

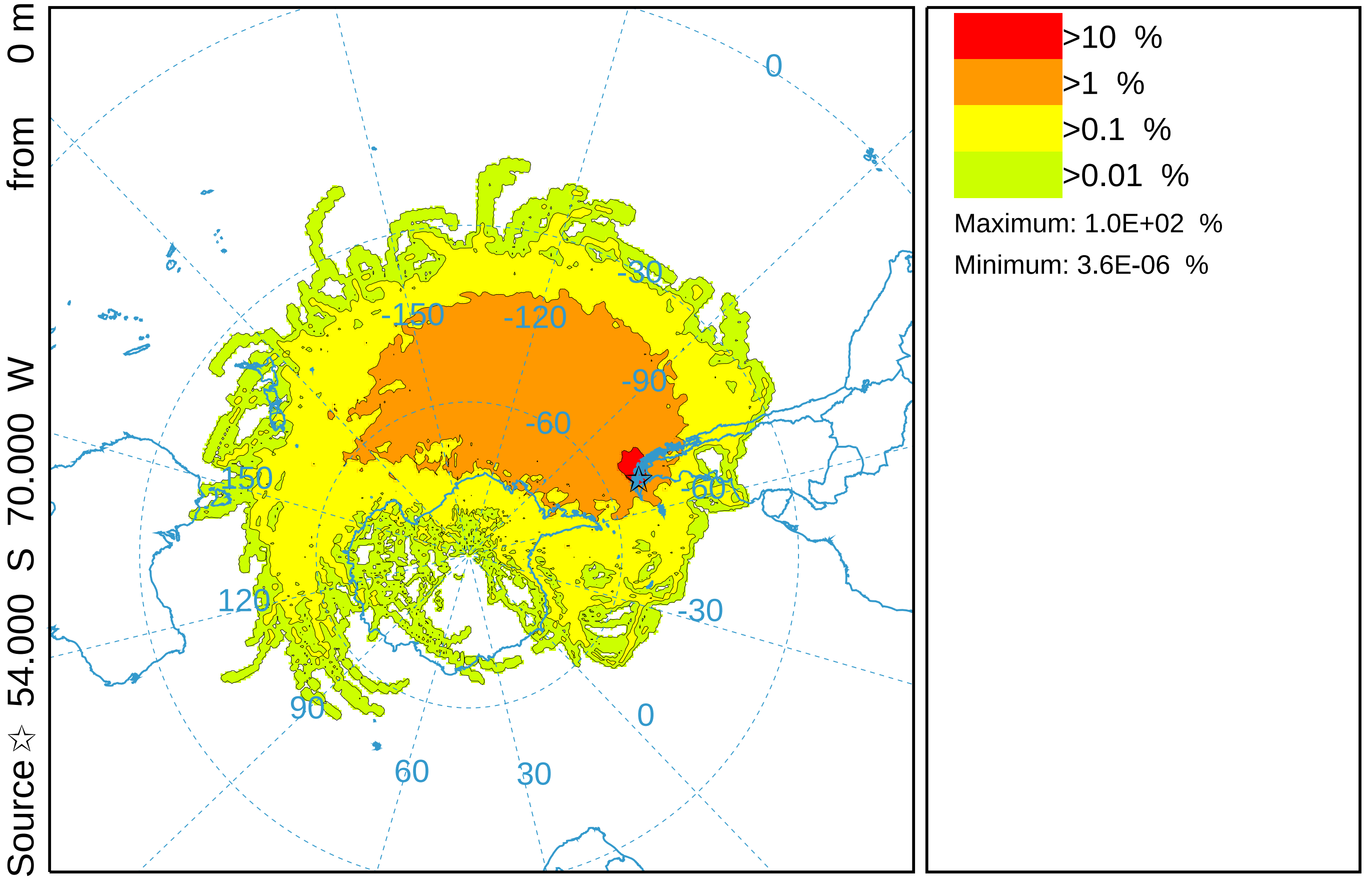

METEOROLOGICAL DATA

Trajectory Frequency Plot year 1976

Values (%) averaged between 0 m and 2000 m
Integrated from 0000 00 to 0000 00 00 (UTC)
Freq Release started at 0000 00 00 (UTC)

Source ☆ 54.000 S 70.000 W from 0 m

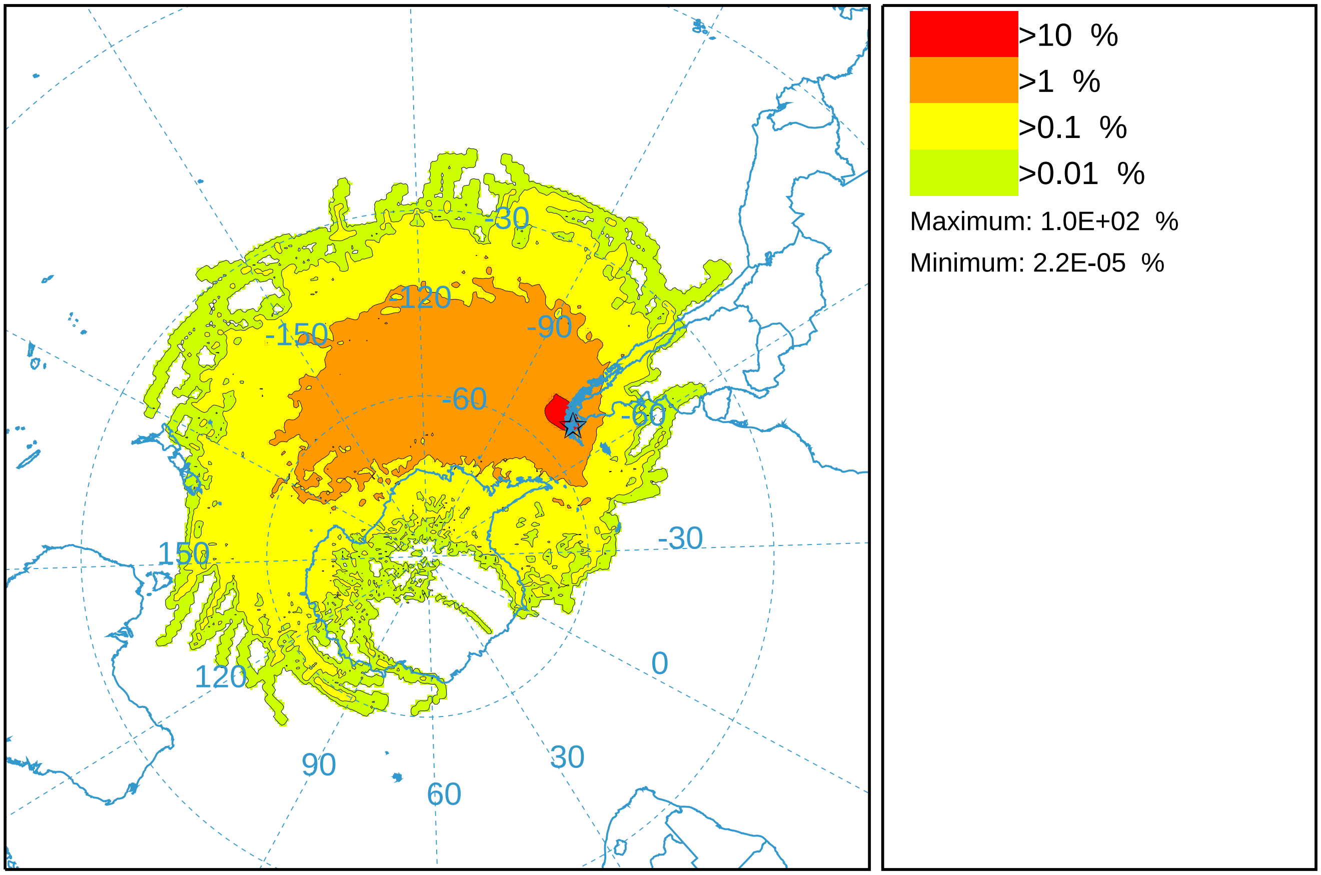

METEOROLOGICAL DATA

Trajectory Frequency Plot year 1977
Values (%) averaged between 0 m and 2000 m
Integrated from 0000 00 to 0000 00 00 (UTC)
Freq Release started at 0000 00 00 (UTC)

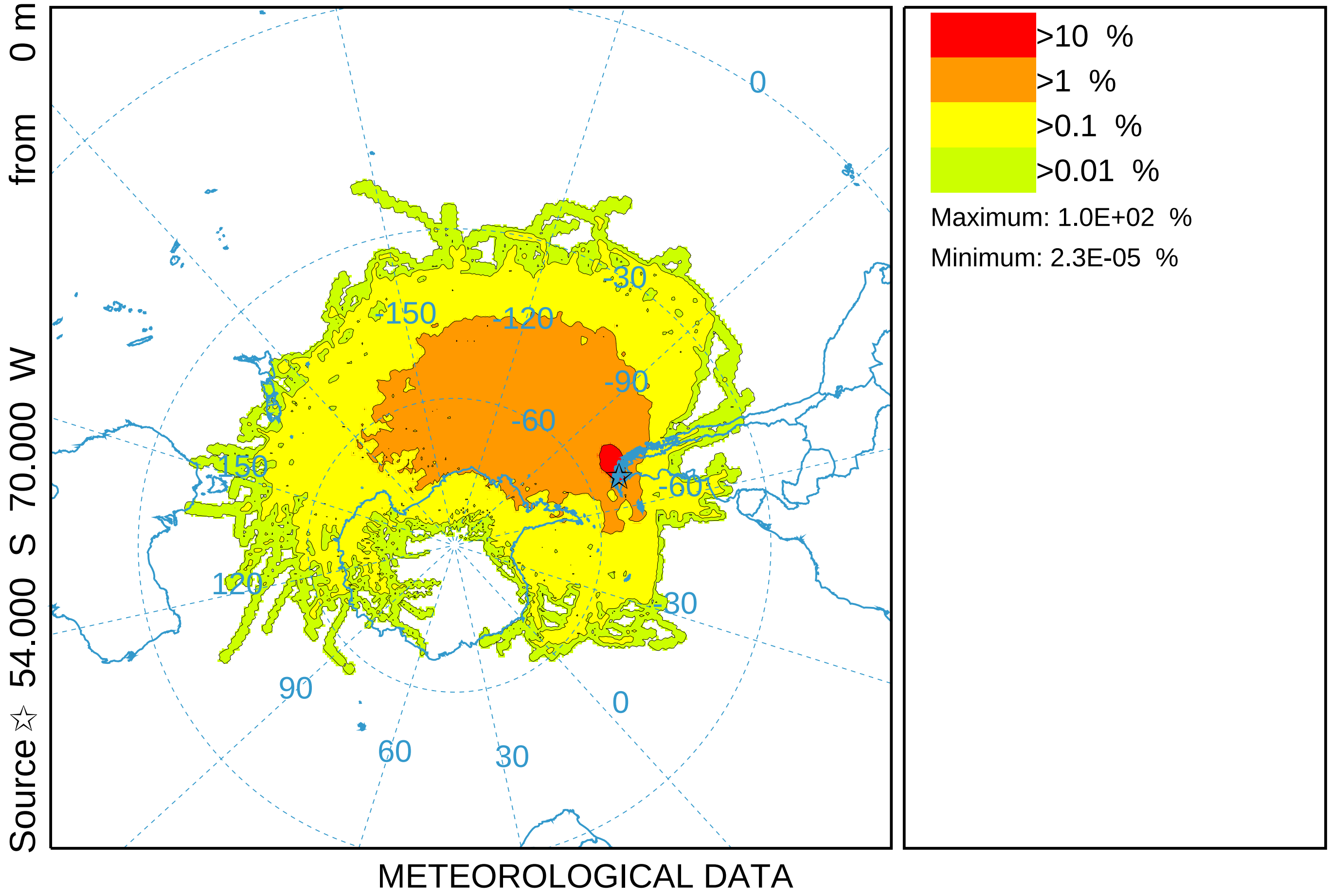

Trajectory Frequency Plot year 1978
Values (%) averaged between 0 m and 2000 m
Integrated from 0000 00 to 0000 00 00 (UTC)
Freq Release started at 0000 00 00 (UTC)

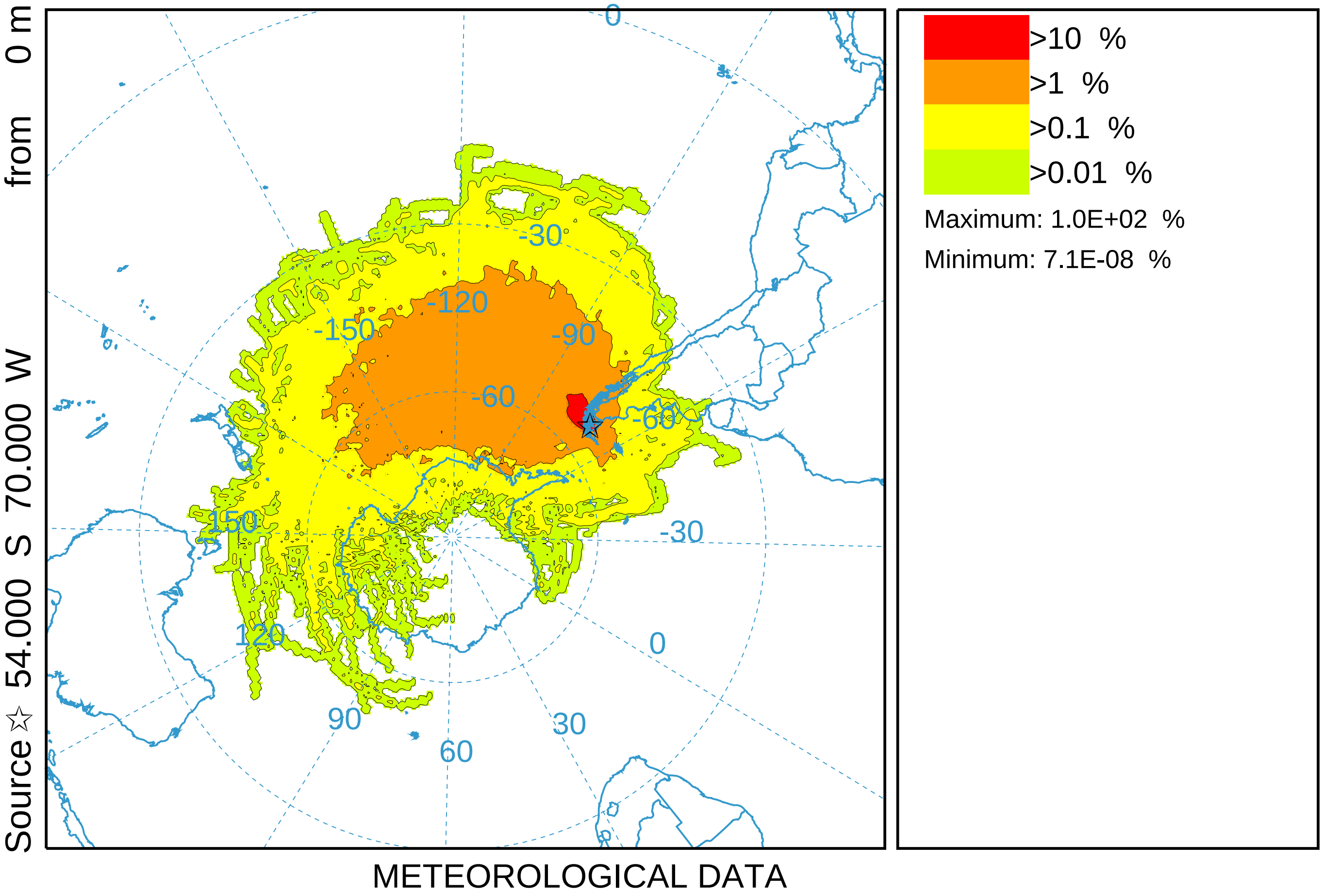

Trajectory Frequency Plot year 1979
Values (%) averaged between 0 m and 2000 m
Integrated from 0000 00 to 0000 00 00 (UTC)
Freq Release started at 0000 00 00 (UTC)

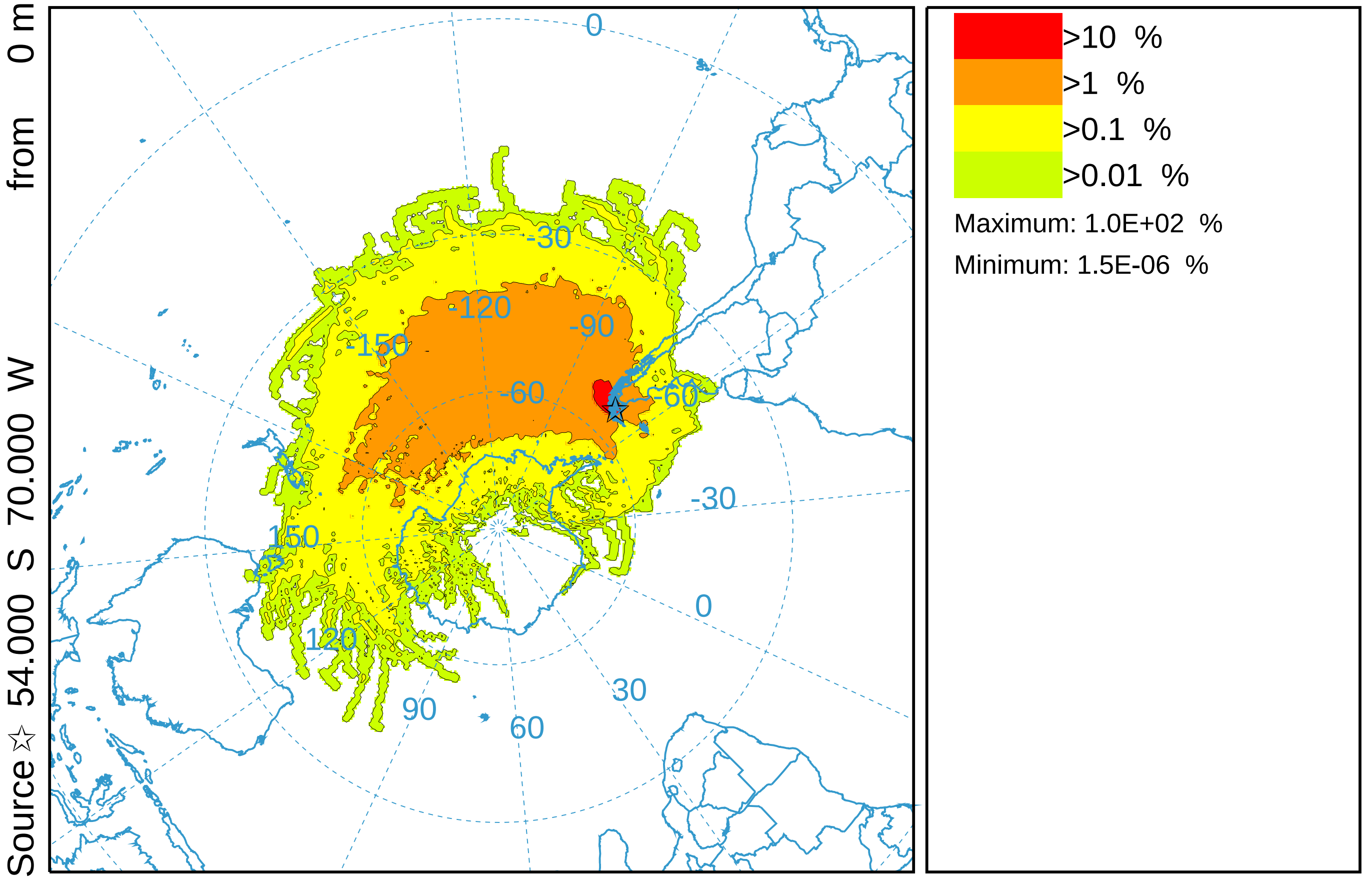

METEOROLOGICAL DATA

Trajectory Frequency Plot year 1980
Values (%) averaged between 0 m and 2000 m
Integrated from 0000 00 to 0000 00 00 (UTC)
Freq Release started at 0000 00 00 (UTC)

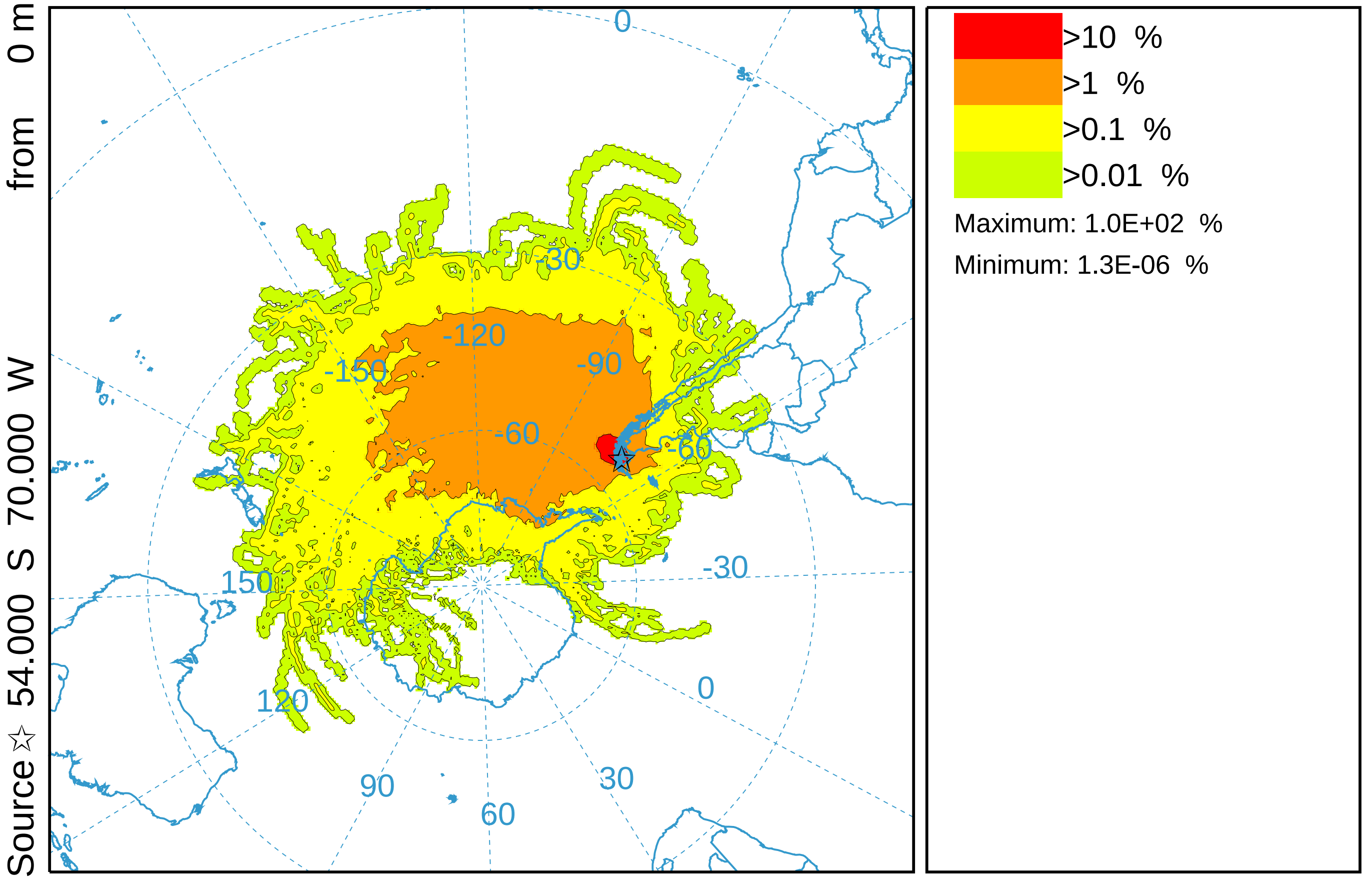

METEOROLOGICAL DATA

Trajectory Frequency Plot year 1981

Values (%) averaged between 0 m and 2000 m

Integrated from 0000 00 to 0000 00 00 (UTC)

Freq Release started at 0000 00 00 (UTC)

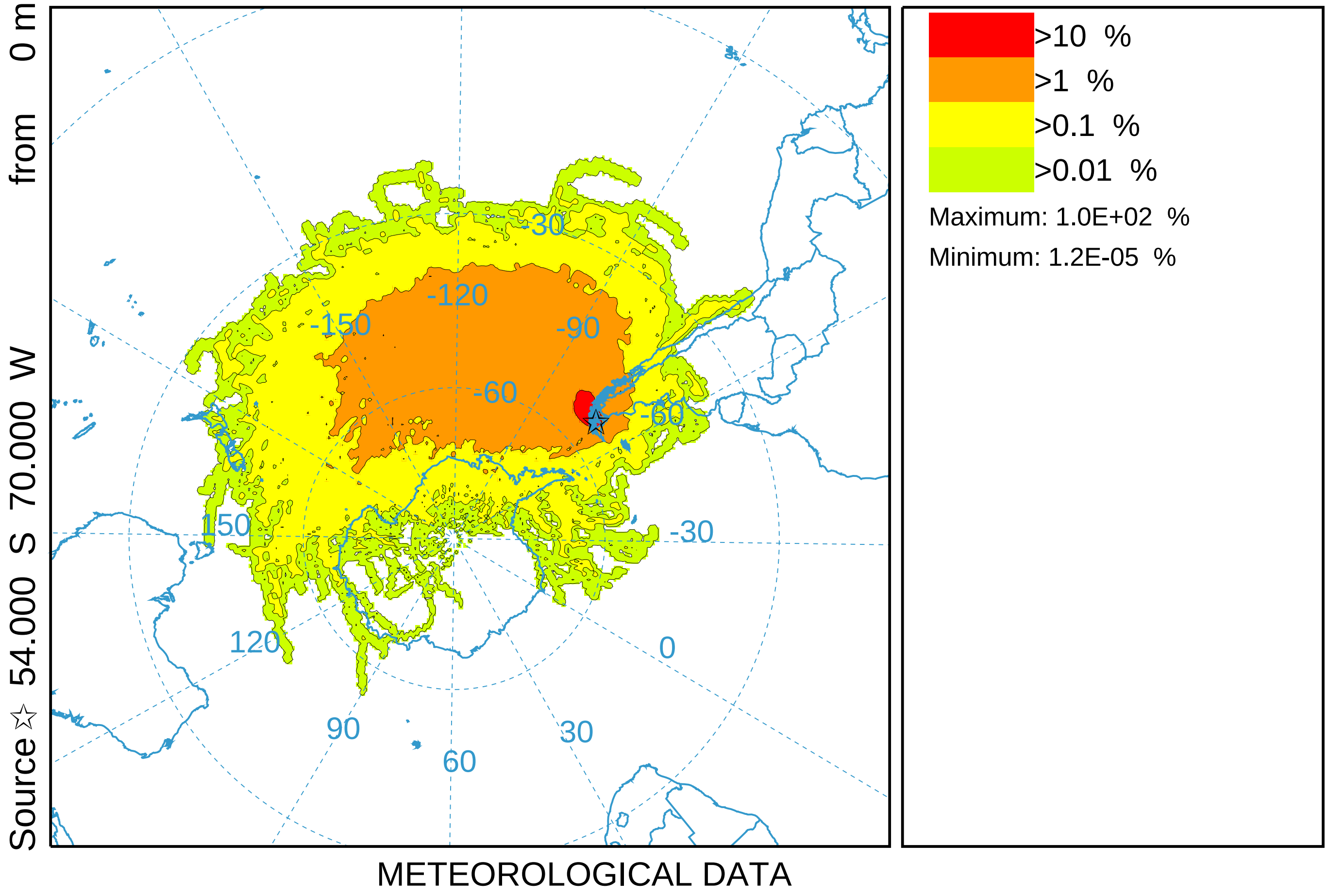

Trajectory Frequency Plot year 1982
Values (%) averaged between 0 m and 2000 m
Integrated from 0000 00 to 0000 00 00 (UTC)
Freq Release started at 0000 00 00 (UTC)

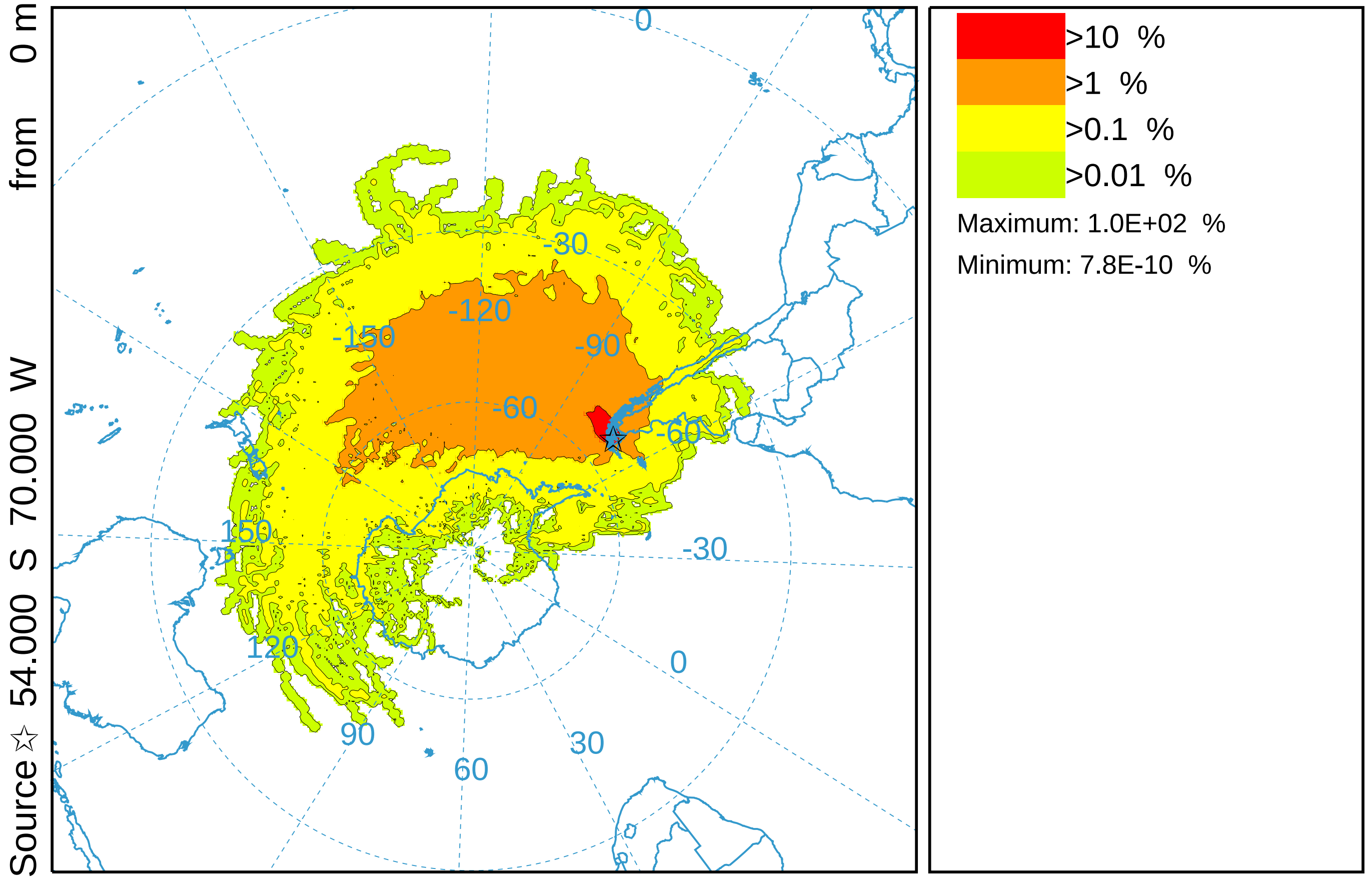

METEOROLOGICAL DATA

Trajectory Frequency Plot year 1983

Values (%) averaged between 0 m and 2000 m

Integrated from 0000 00 to 0000 00 00 (UTC)

Freq Release started at 0000 00 00 (UTC)

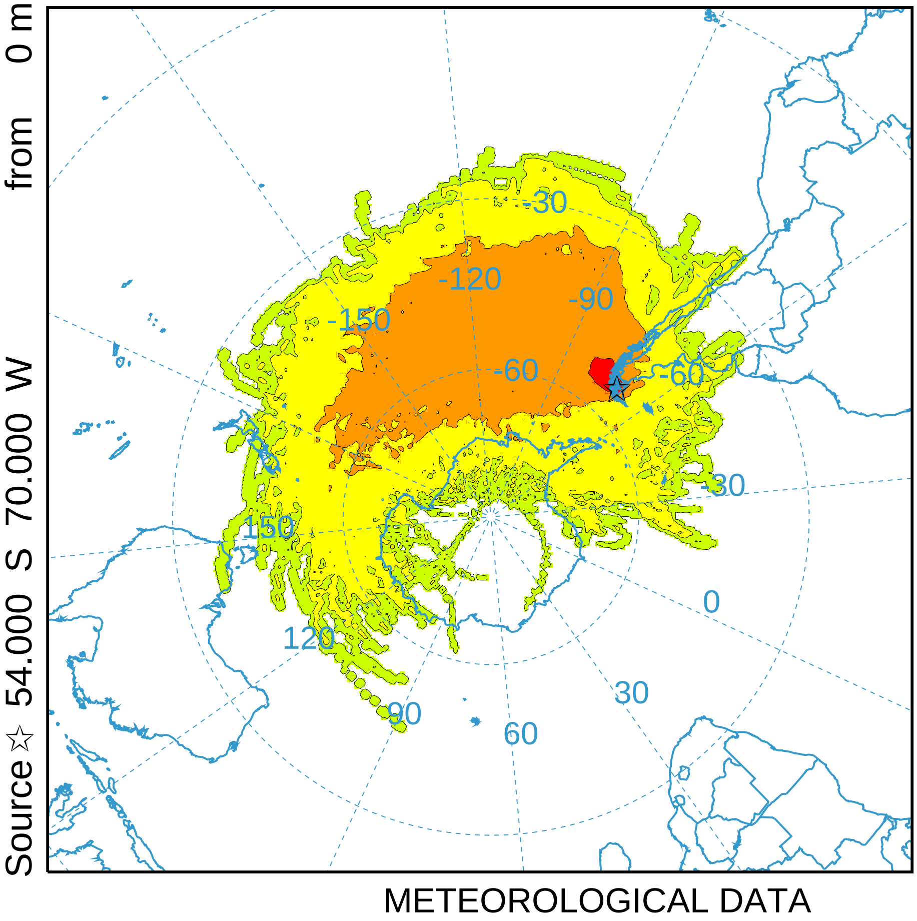

>10 %

>1 %

>0.1 %

>0.01 %

Maximum: 1.0E+02 %

Minimum: 6.8E-07 %

Trajectory Frequency Plot year 1984
Values (%) averaged between 0 m and 2000 m
Integrated from 0000 00 to 0000 00 00 (UTC)
Freq Release started at 0000 00 00 (UTC)

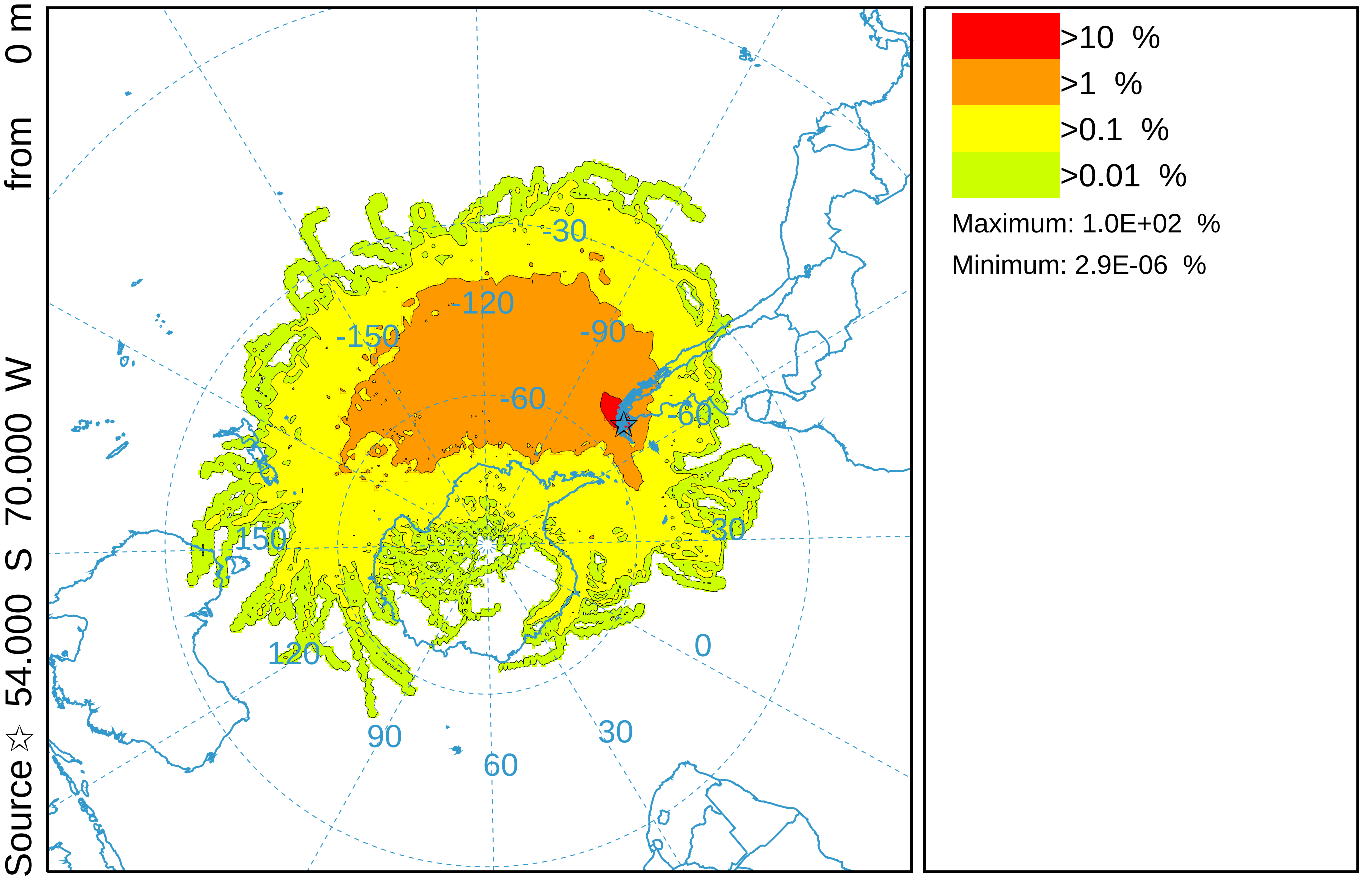

METEOROLOGICAL DATA

Trajectory Frequency Plot year 1985
Values (%) averaged between 0 m and 2000 m
Integrated from 0000 00 to 0000 00 00 (UTC)
Freq Release started at 0000 00 00 (UTC)

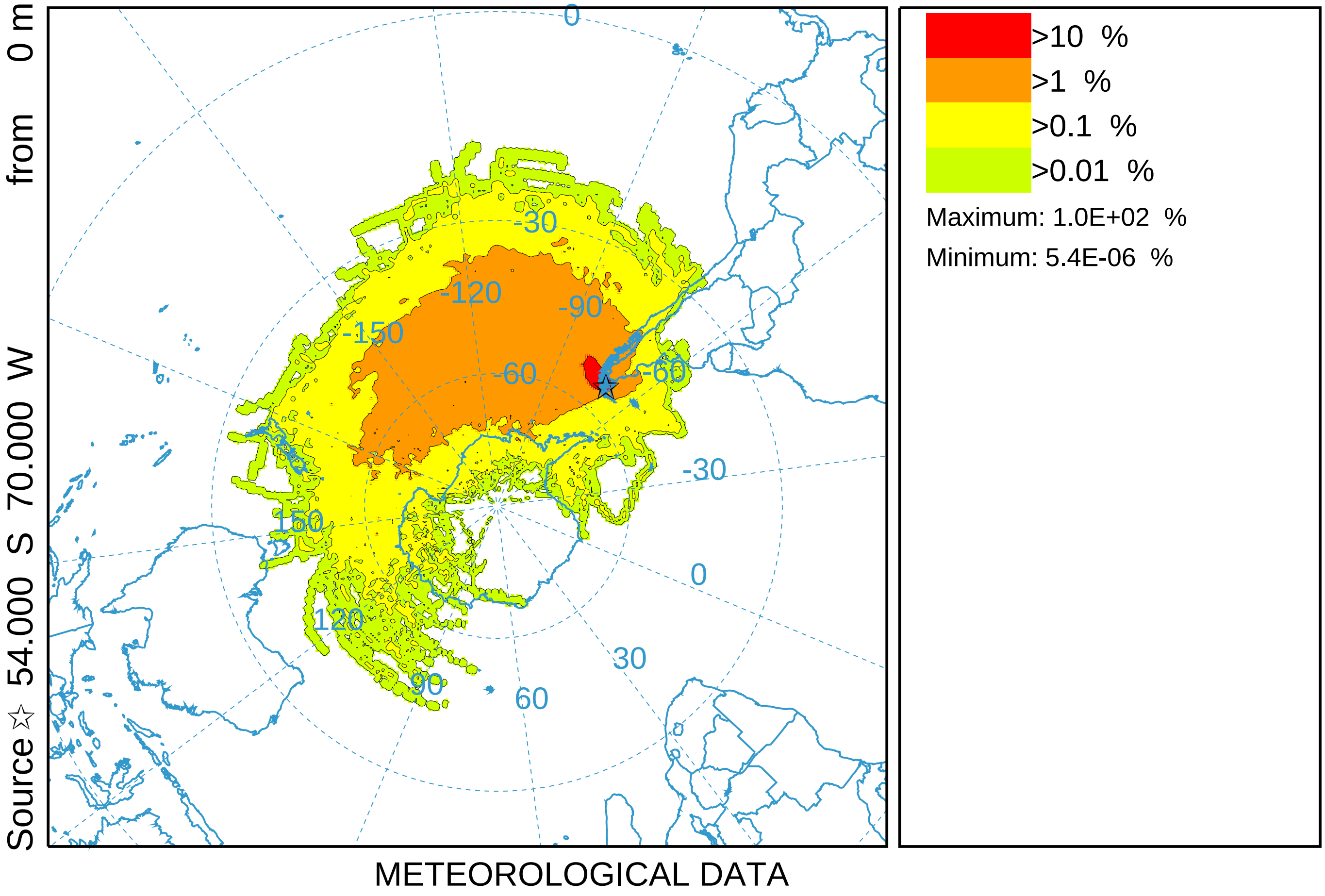

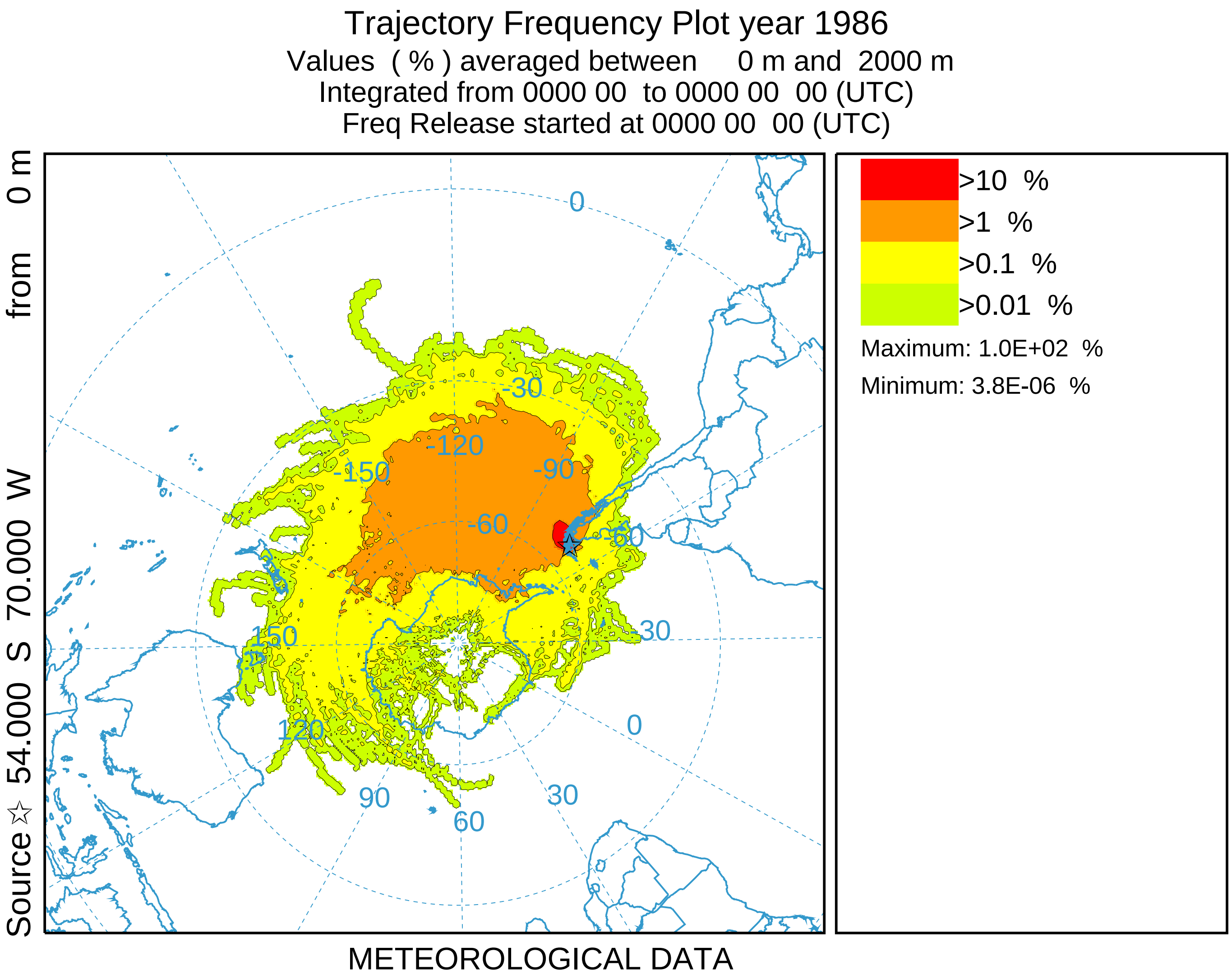

Trajectory Frequency Plot year 1986
Values (%) averaged between 0 m and 2000 m
Integrated from 0000 00 to 0000 00 00 (UTC)
Freq Release started at 0000 00 00 (UTC)
Source ☆ 54.000 S 70.000 W from 0 m
>10 %
>1 %
>0.1 %
>0.01 %
Maximum: 1.0E+02 %
Minimum: 3.8E-06 %
0
-30
-120
-90
-150
-60
-60
-30
150
0
120
90
60
30
METEOROLOGICAL DATA

Trajectory Frequency Plot year 1987

Values (%) averaged between 0 m and 2000 m

Integrated from 0000 00 to 0000 00 00 (UTC)

Freq Release started at 0000 00 00 (UTC)

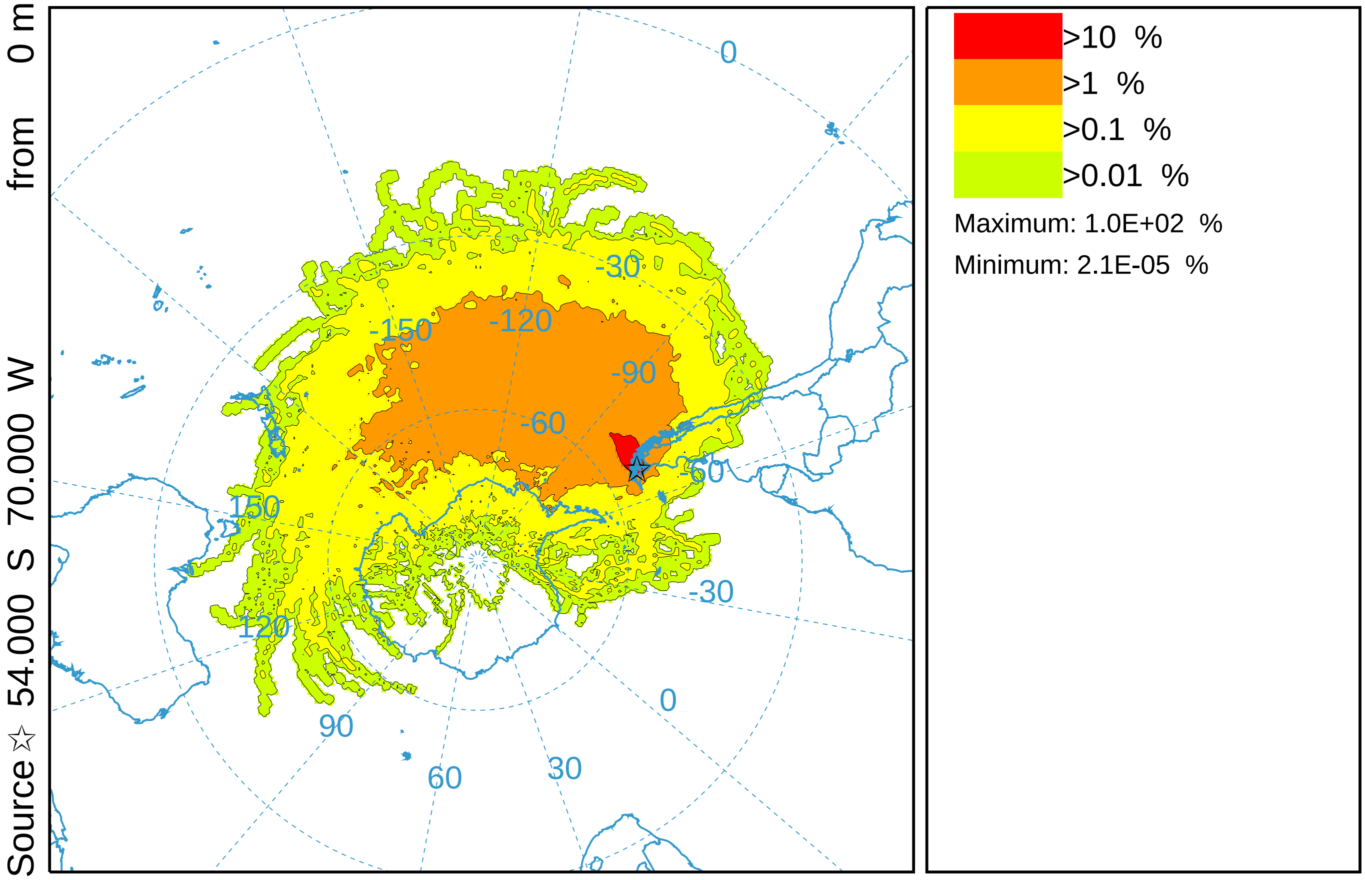

METEOROLOGICAL DATA

Trajectory Frequency Plot year 1988

Values (%) averaged between 0 m and 2000 m

Integrated from 0000 00 to 0000 00 00 (UTC)

Freq Release started at 0000 00 00 (UTC)

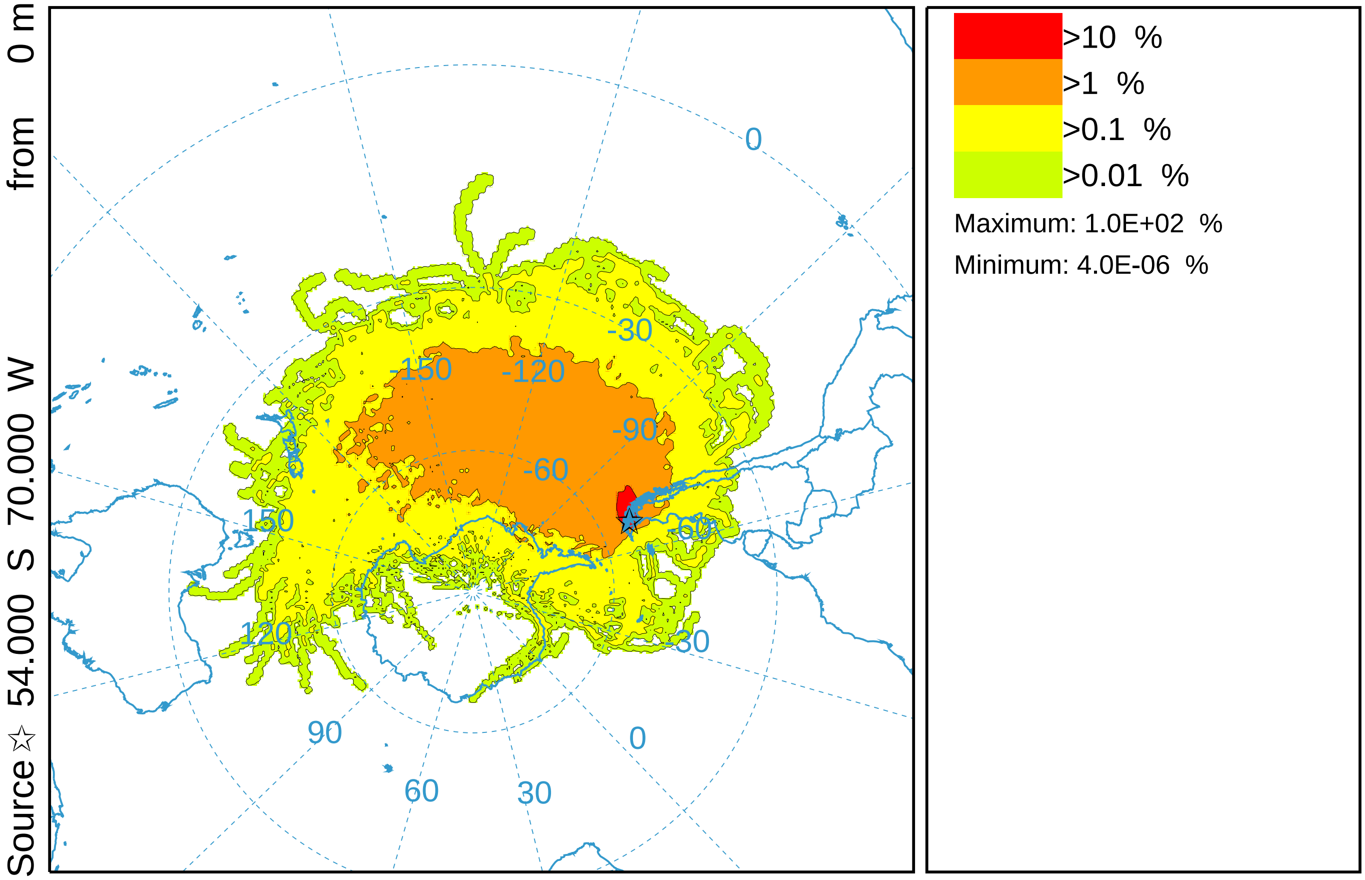

METEOROLOGICAL DATA

Trajectory Frequency Plot year 1989

Values (%) averaged between 0 m and 2000 m

Integrated from 0000 00 to 0000 00 00 (UTC)

Freq Release started at 0000 00 00 (UTC)

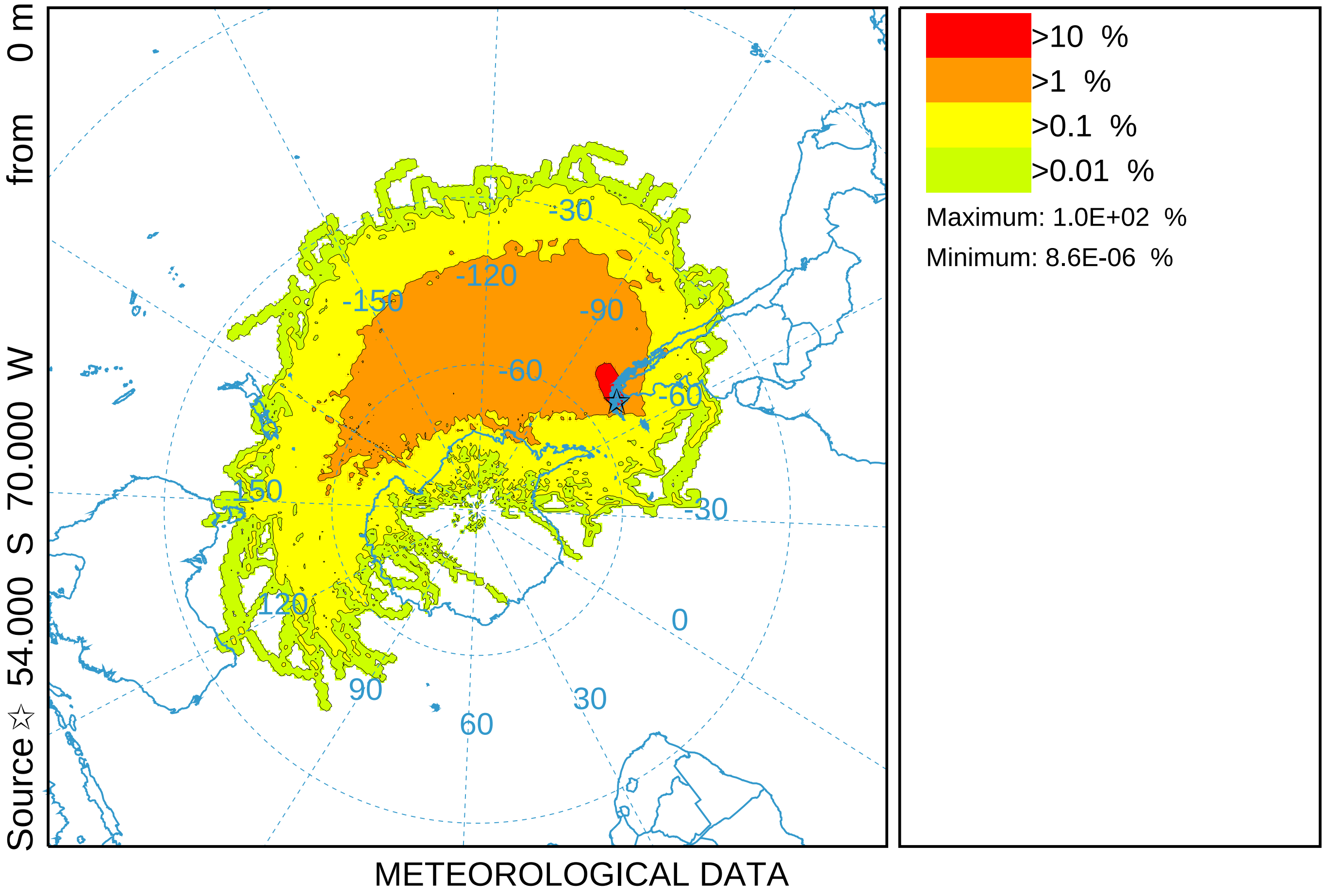

Trajectory Frequency Plot year 1990

Values (%) averaged between 0 m and 2000 m
Integrated from 0000 00 to 0000 00 00 (UTC)
Freq Release started at 0000 00 00 (UTC)

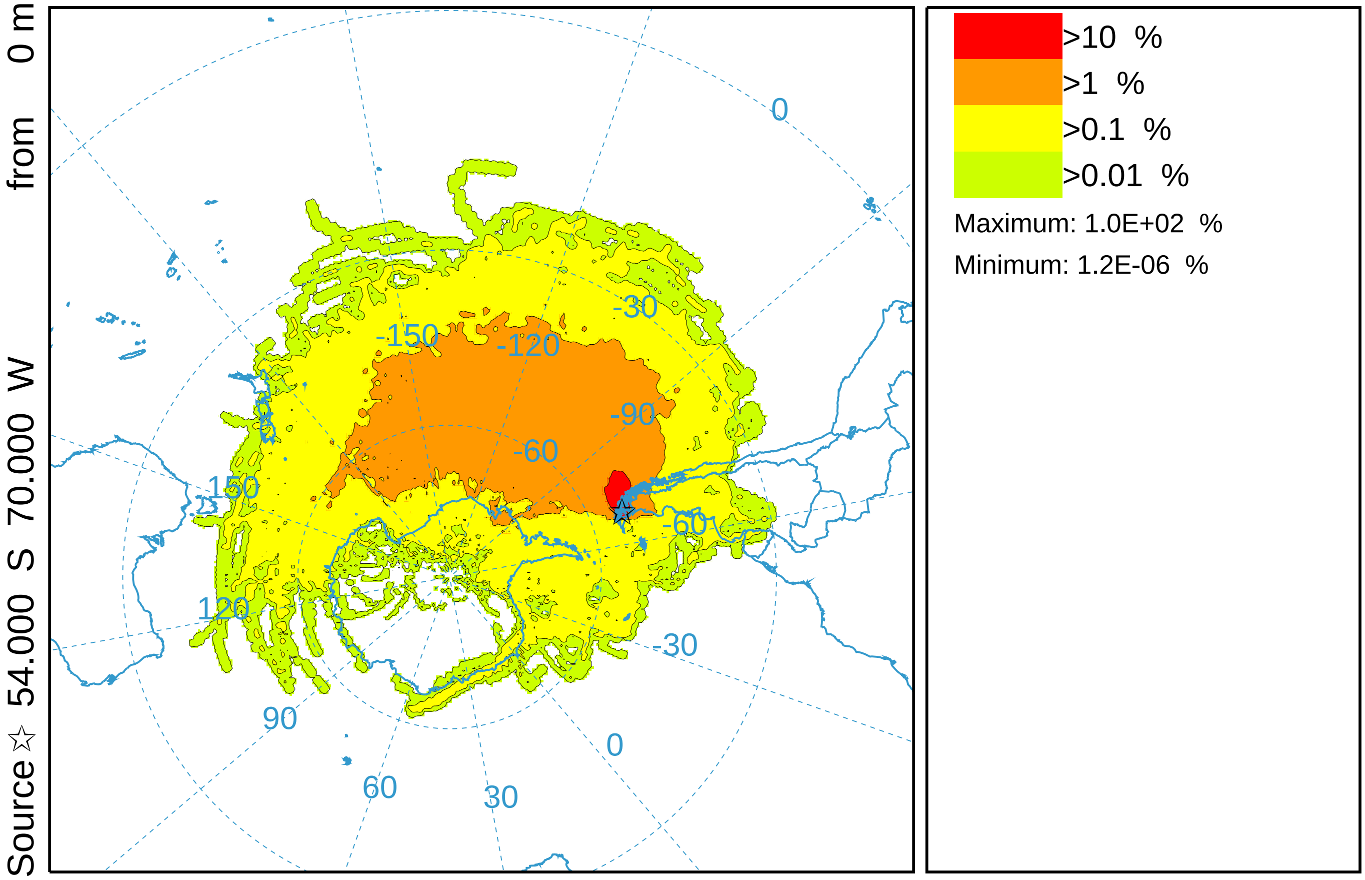

METEOROLOGICAL DATA

Trajectory Frequency Plot year 1991

Values (%) averaged between 0 m and 2000 m
Integrated from 0000 00 to 0000 00 00 (UTC)
Freq Release started at 0000 00 00 (UTC)

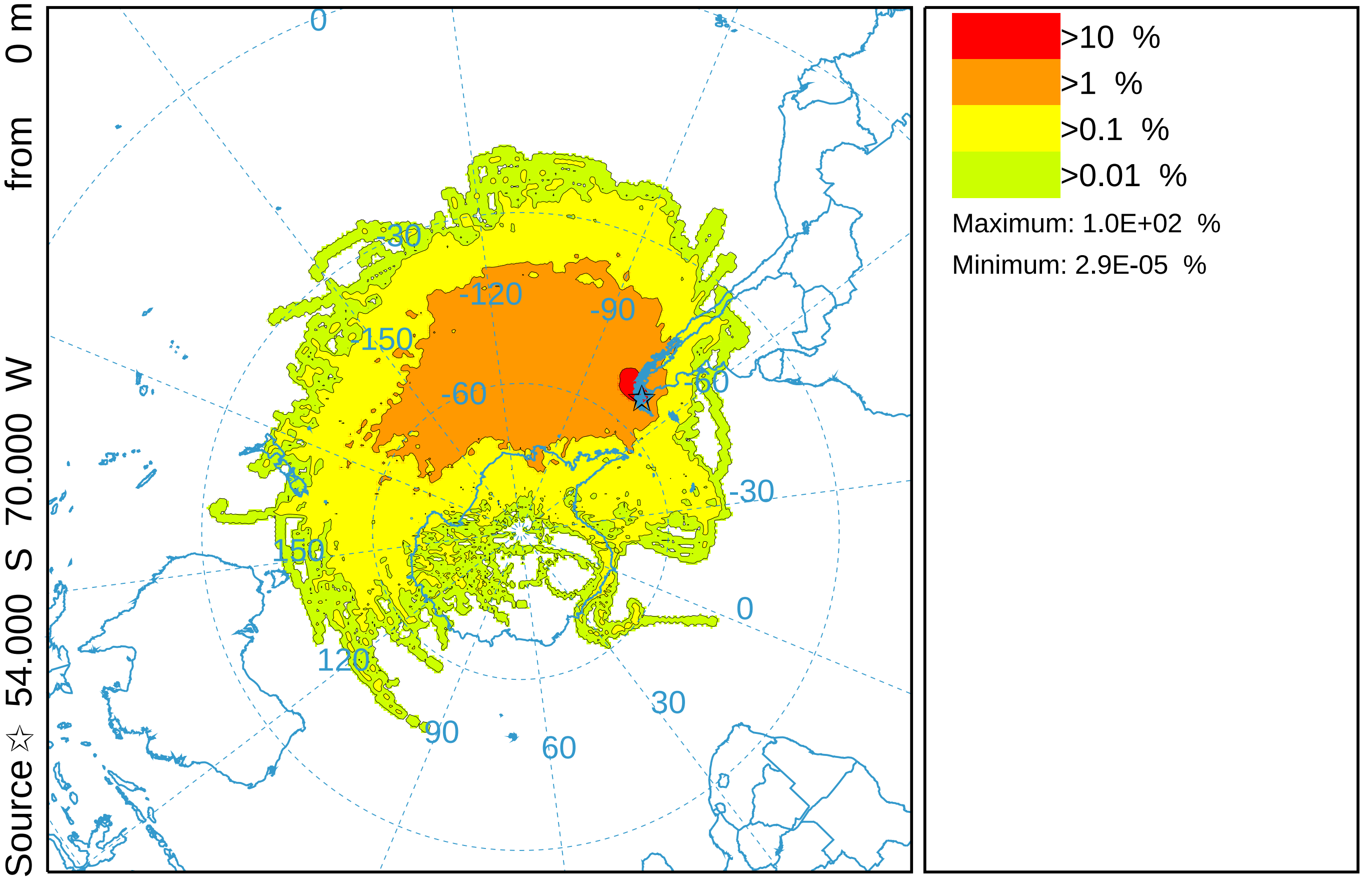

METEOROLOGICAL DATA

Trajectory Frequency Plot year 1992

Values (%) averaged between 0 m and 2000 m
Integrated from 0000 00 to 0000 00 00 (UTC)
Freq Release started at 0000 00 00 (UTC)

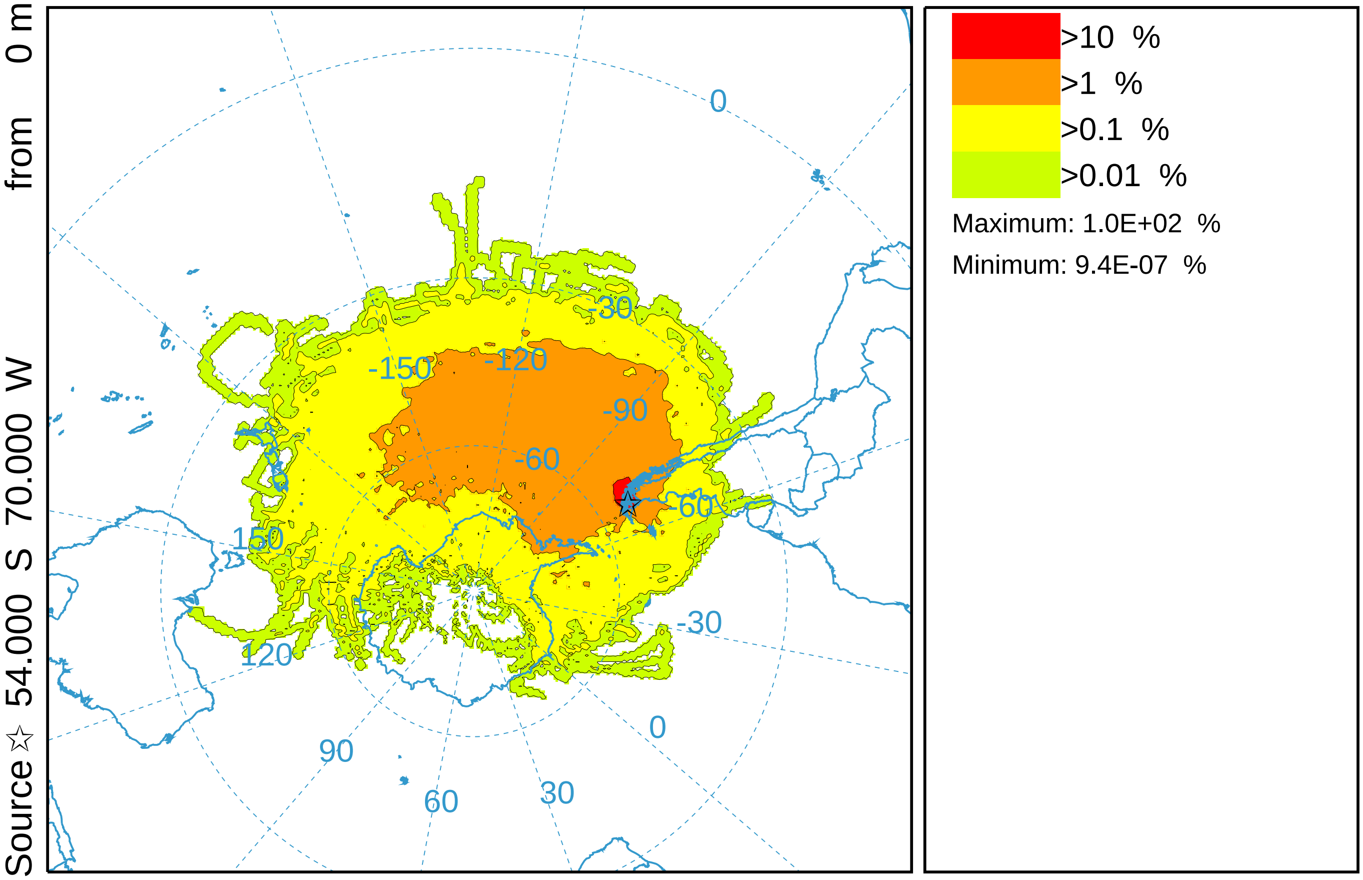

METEOROLOGICAL DATA

Trajectory Frequency Plot year 1993
Values (%) averaged between 0 m and 2000 m
Integrated from 0000 00 to 0000 00 00 (UTC)
Freq Release started at 0000 00 00 (UTC)

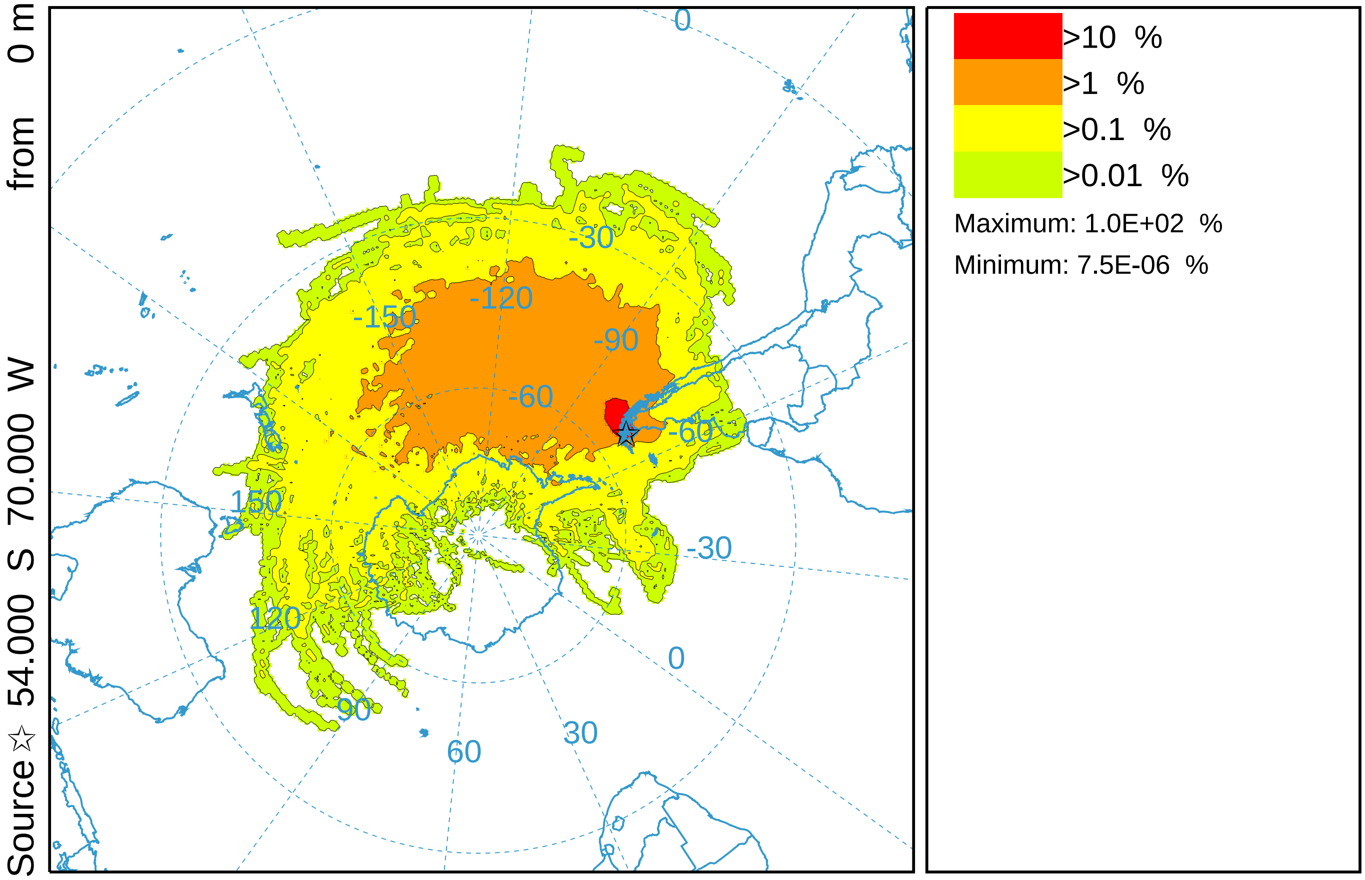

METEOROLOGICAL DATA

Trajectory Frequency Plot year 1994

Values (%) averaged between 0 m and 2000 m

Integrated from 0000 00 to 0000 00 00 (UTC)

Freq Release started at 0000 00 00 (UTC)

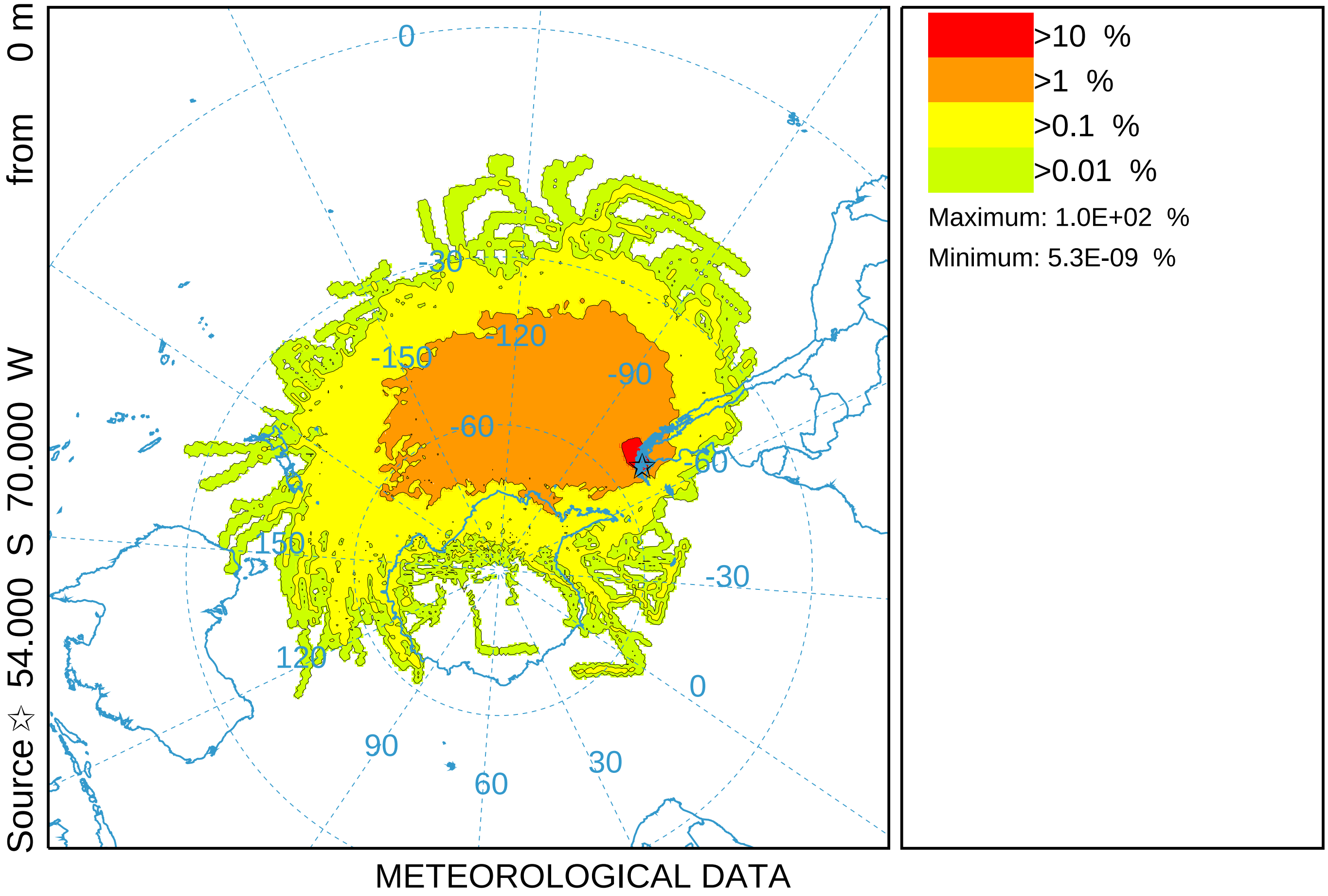

Trajectory Frequency Plot year 1995

Values (%) averaged between 0 m and 2000 m

Integrated from 0000 00 to 0000 00 00 (UTC)

Freq Release started at 0000 00 00 (UTC)

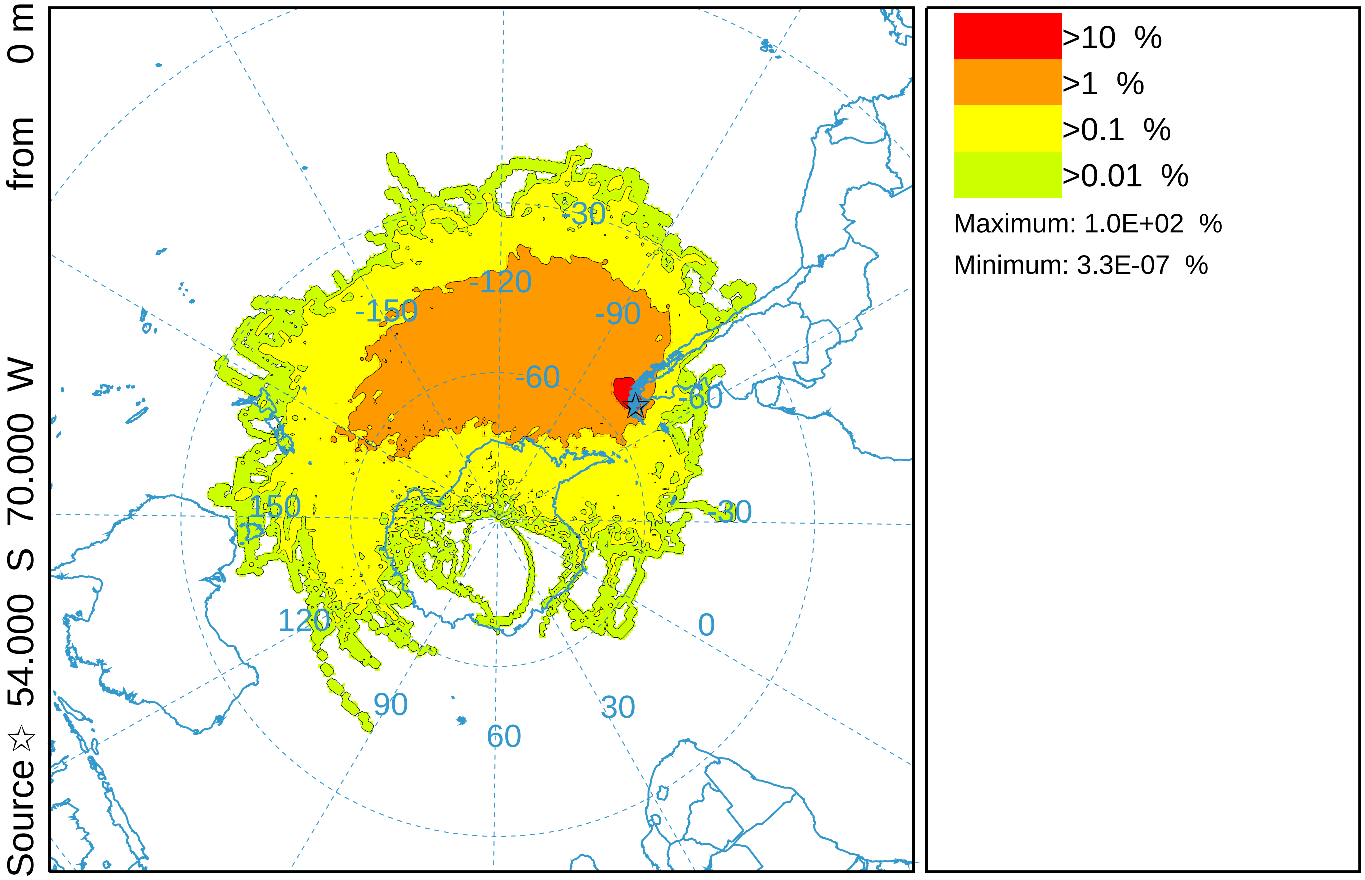

METEOROLOGICAL DATA

Trajectory Frequency Plot year 1996
Values (%) averaged between 0 m and 2000 m
Integrated from 0000 00 to 0000 00 00 (UTC)
Freq Release started at 0000 00 00 (UTC)

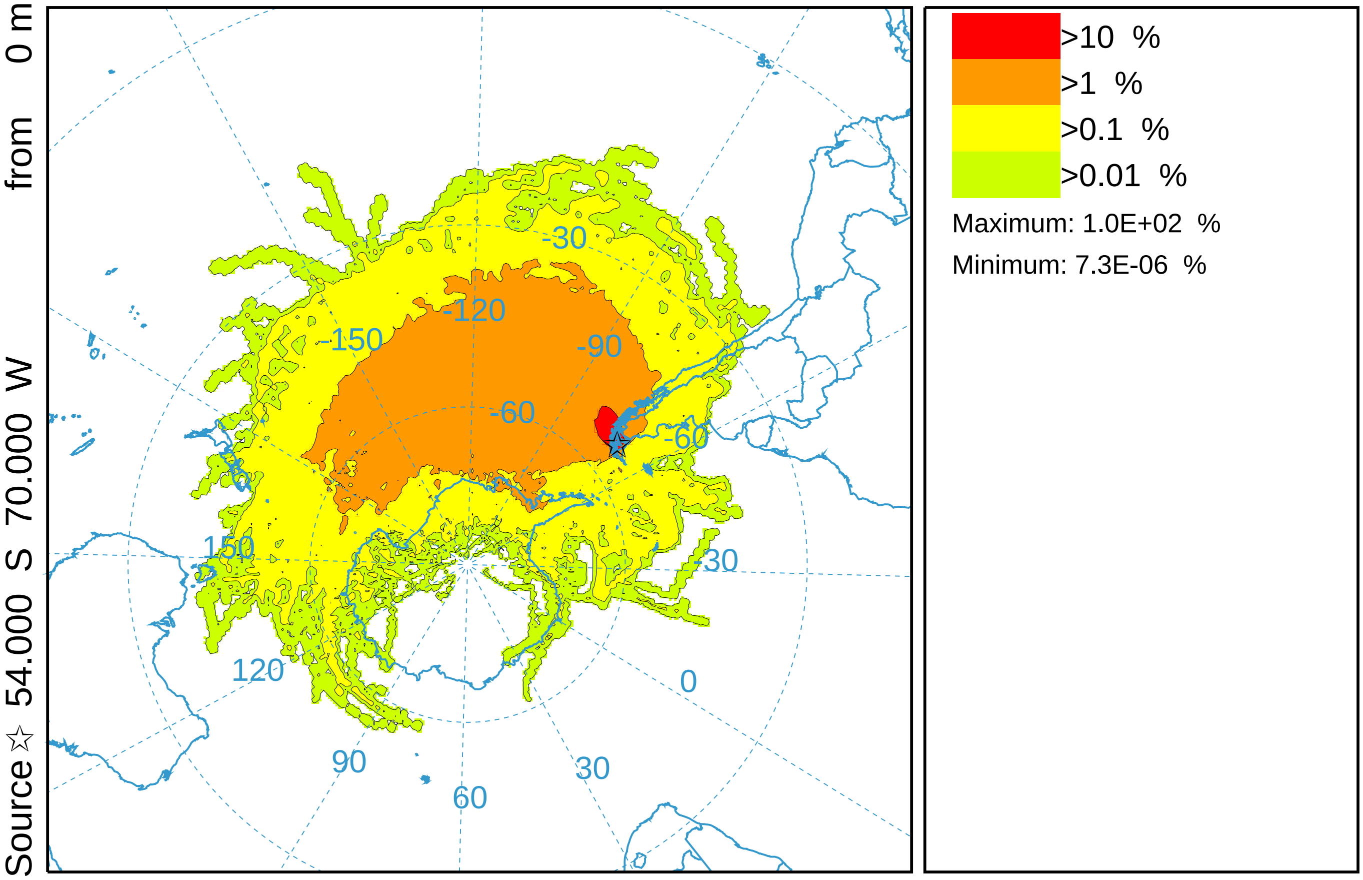

METEOROLOGICAL DATA

Trajectory Frequency Plot year 1997

Values (%) averaged between 0 m and 2000 m

Integrated from 0000 00 to 0000 00 00 (UTC)

Freq Release started at 0000 00 00 (UTC)

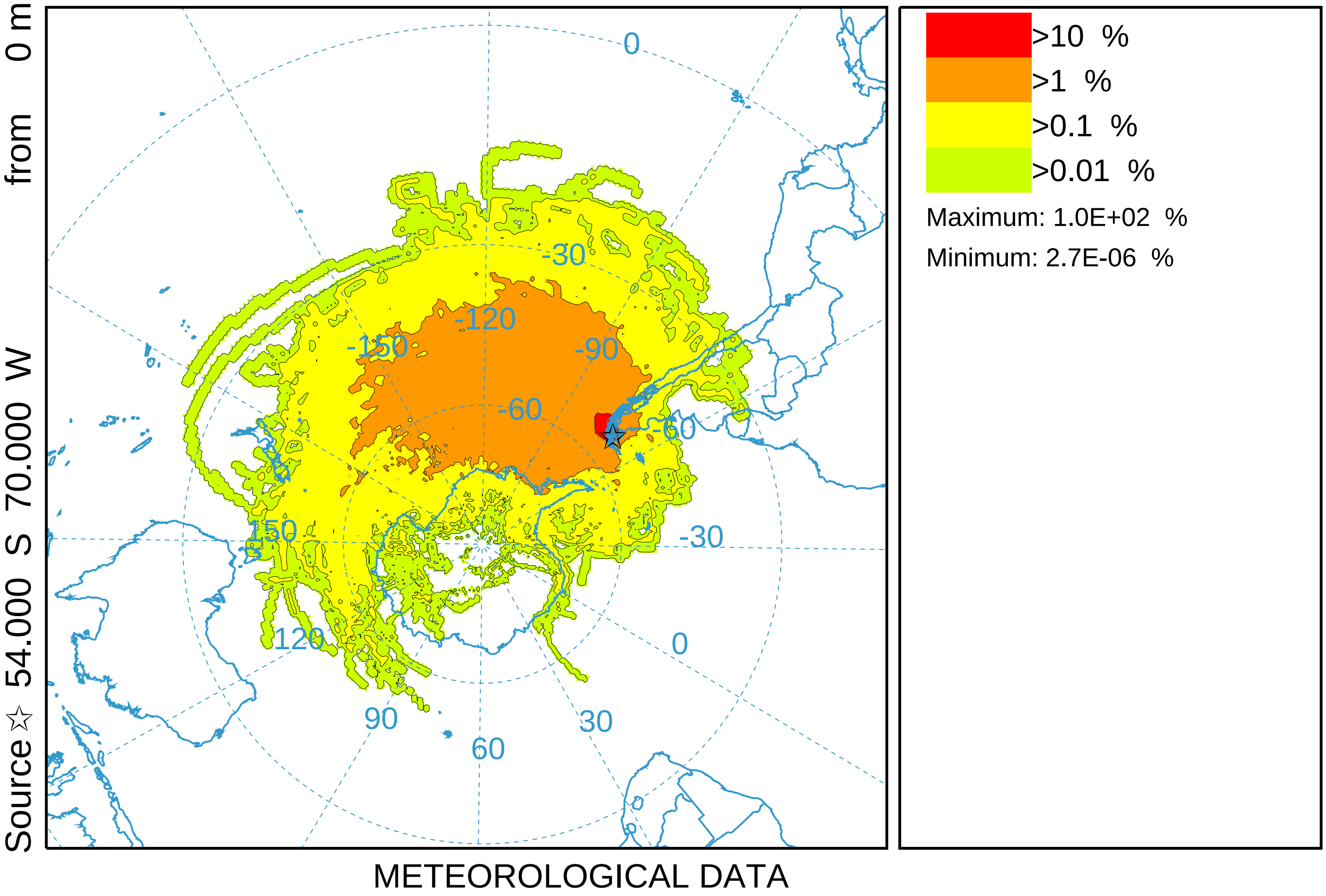

Trajectory Frequency Plot year 1998

Values (%) averaged between 0 m and 2000 m

Integrated from 0000 00 to 0000 00 00 (UTC)

Freq Release started at 0000 00 00 (UTC)

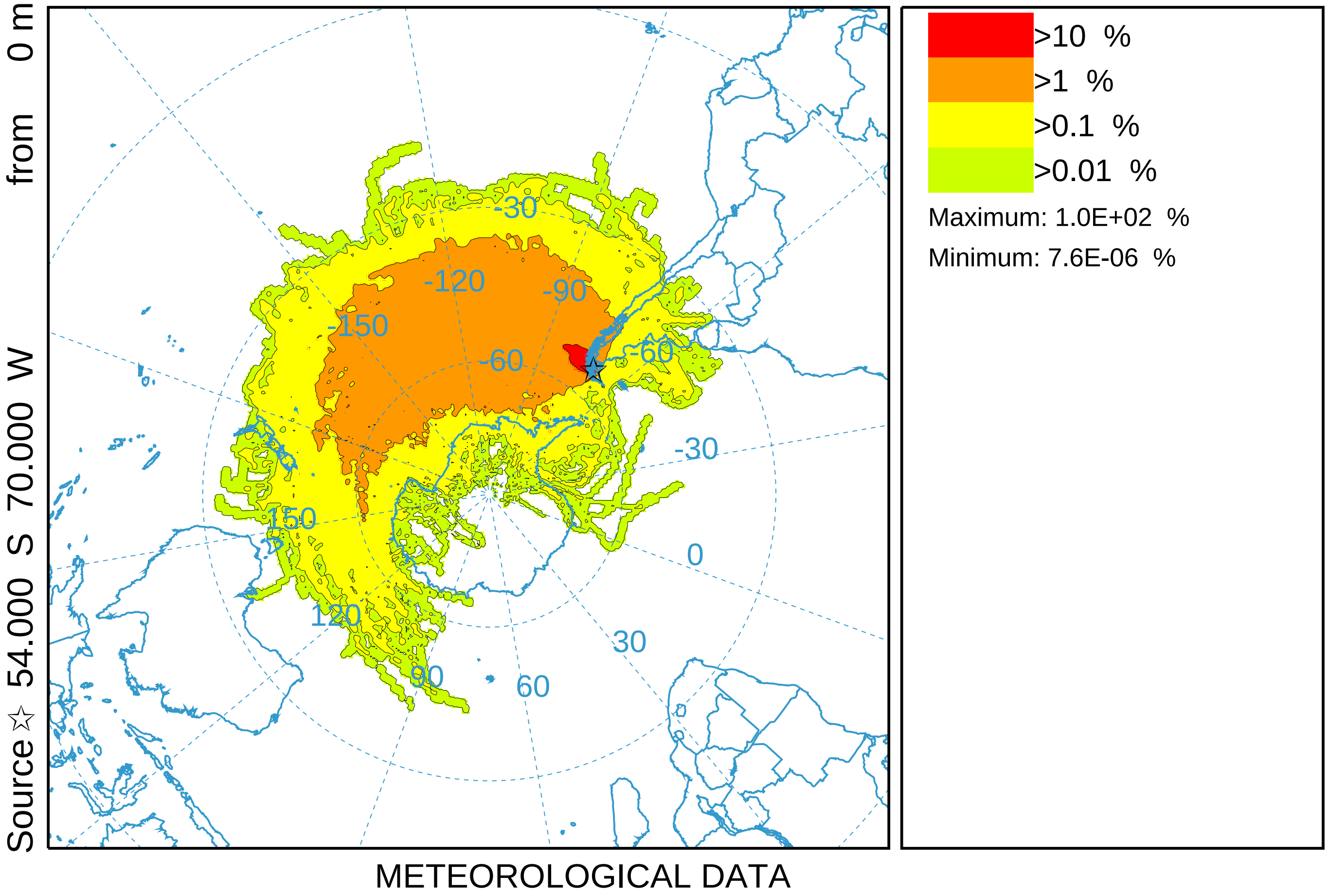

Trajectory Frequency Plot year 1999
Values (%) averaged between 0 m and 2000 m
Integrated from 0000 00 to 0000 00 00 (UTC)
Freq Release started at 0000 00 00 (UTC)

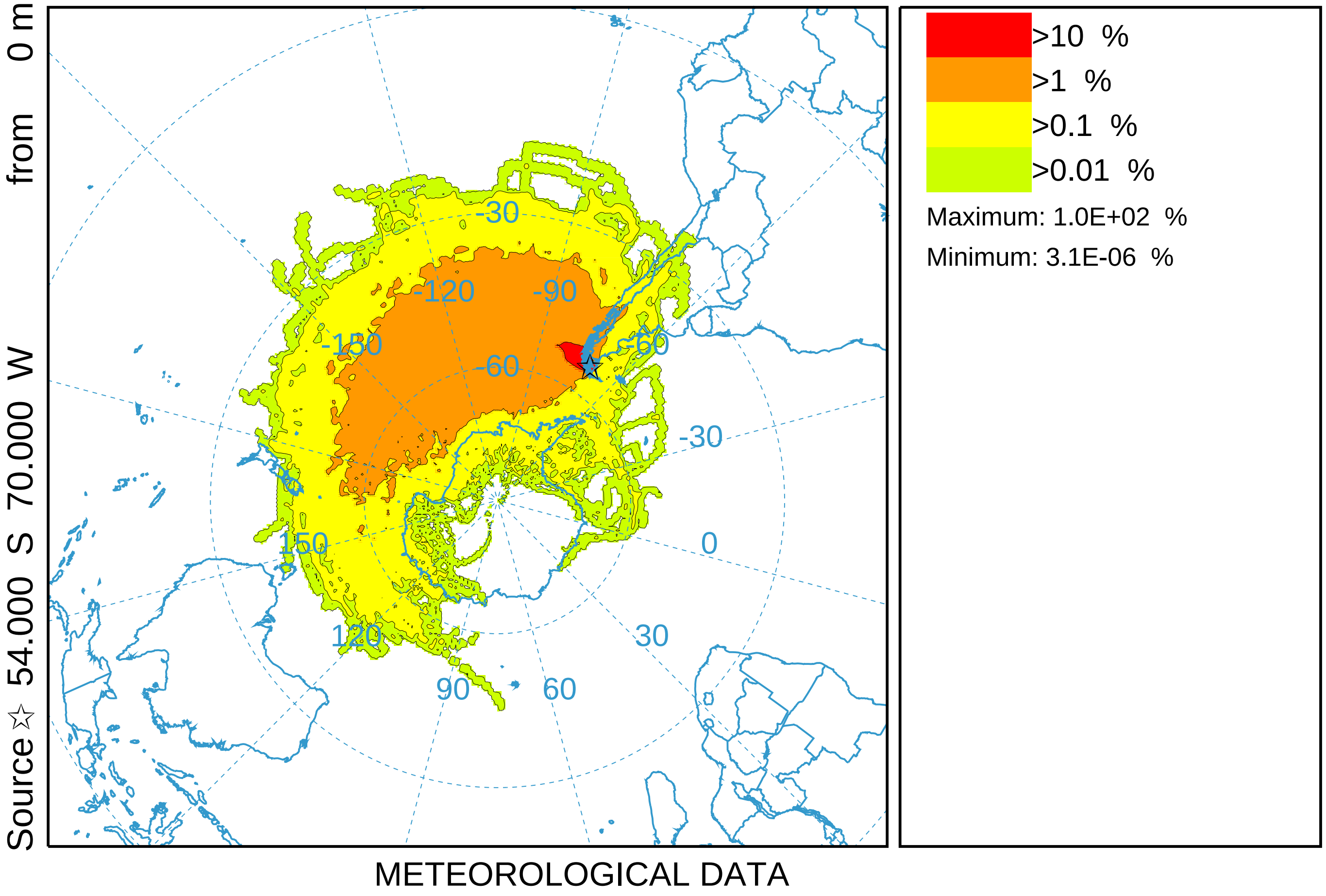

Trajectory Frequency Plot year 2000
Values (%) averaged between 0 m and 2000 m
Integrated from 0000 00 to 0000 00 00 (UTC)
Freq Release started at 0000 00 00 (UTC)

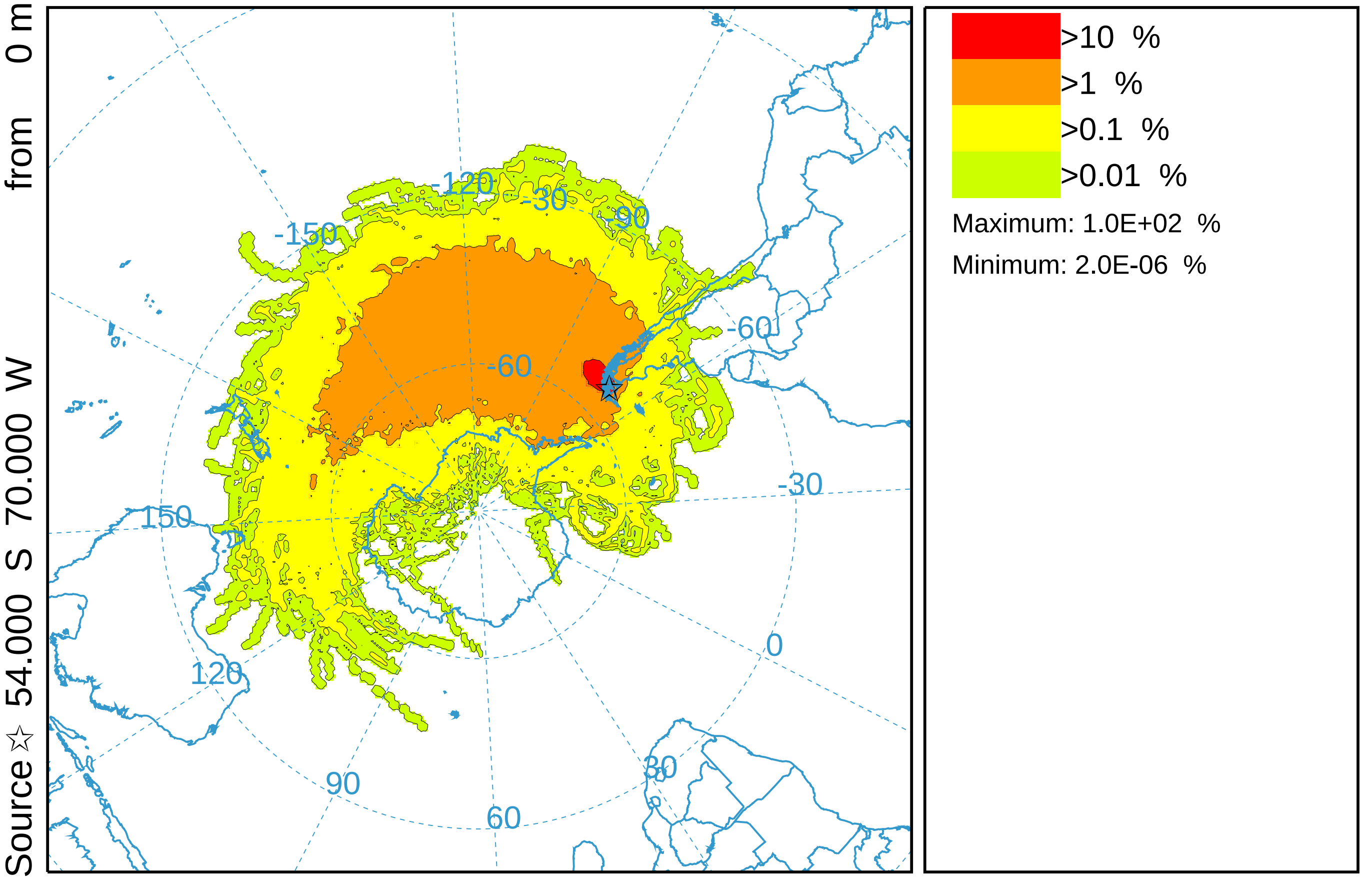

METEOROLOGICAL DATA

Trajectory Frequency Plot year 2001

Values (%) averaged between 0 m and 2000 m

Integrated from 0000 00 to 0000 00 00 (UTC)

Freq Release started at 0000 00 00 (UTC)

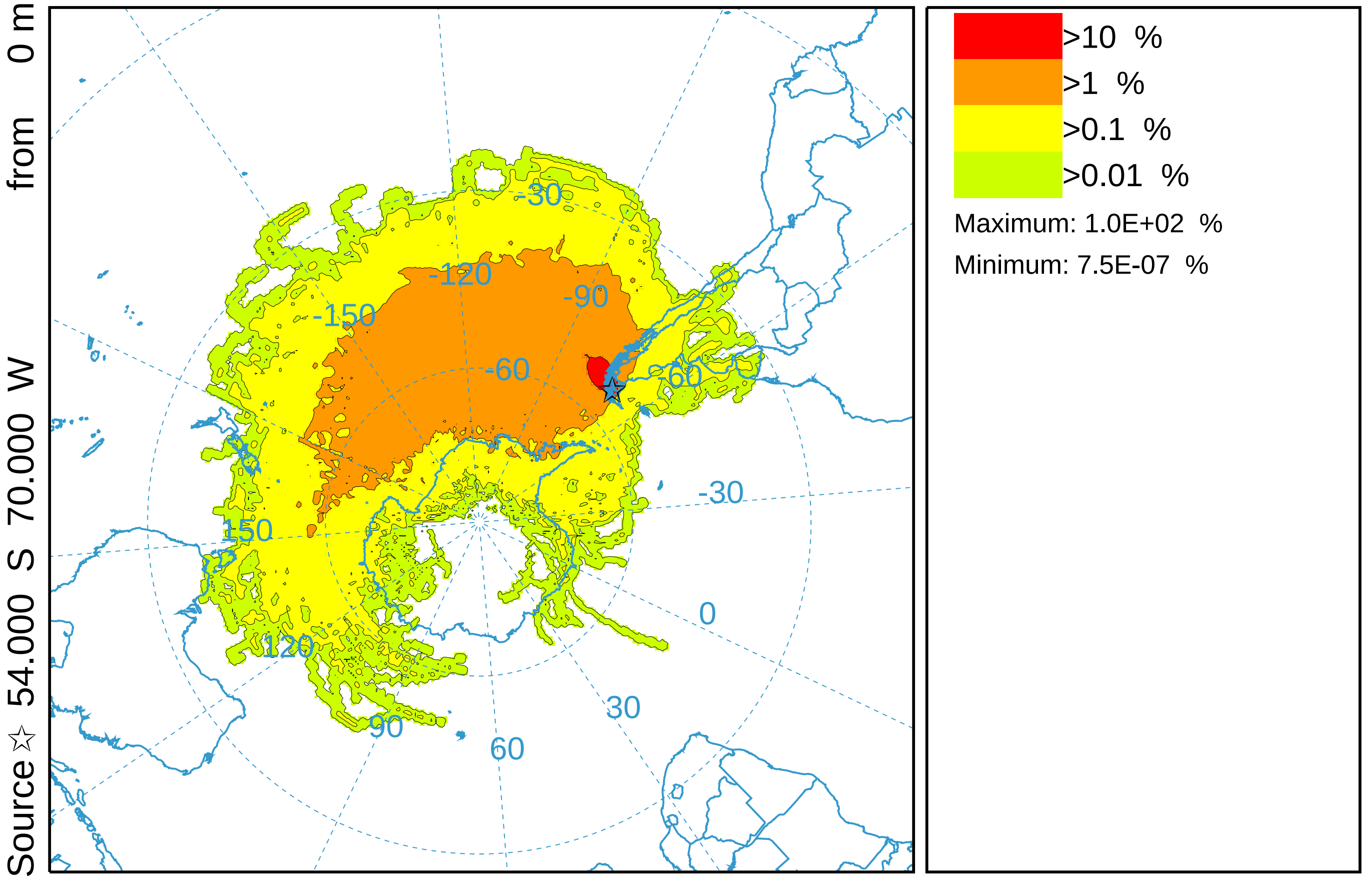

METEOROLOGICAL DATA

Trajectory Frequency Plot year 2002

Values (%) averaged between 0 m and 2000 m
Integrated from 0000 00 to 0000 00 00 (UTC)
Freq Release started at 0000 00 00 (UTC)

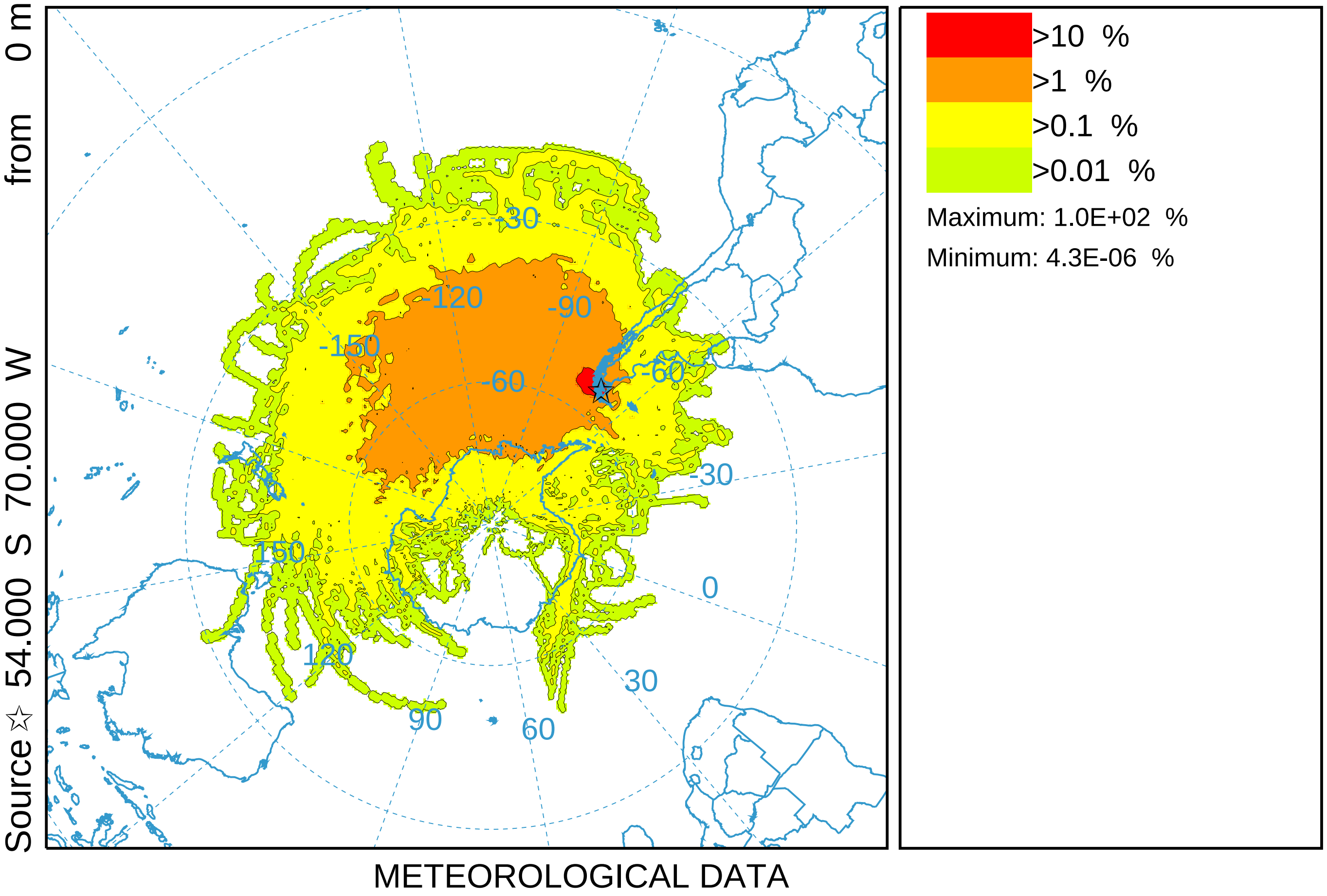

Trajectory Frequency Plot year 2003

Values (%) averaged between 0 m and 2000 m

Integrated from 0000 00 to 0000 00 00 (UTC)

Freq Release started at 0000 00 00 (UTC)

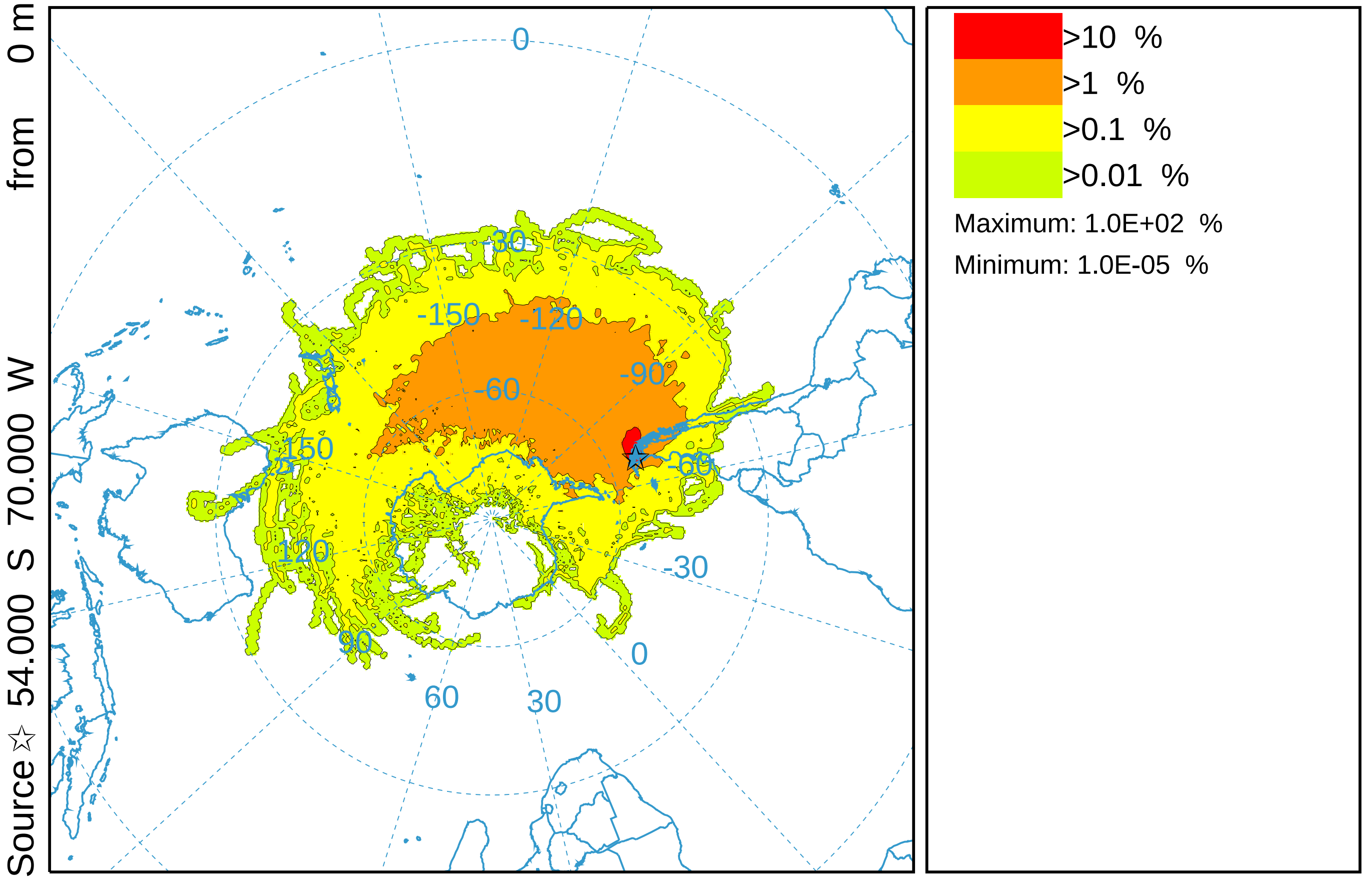

METEOROLOGICAL DATA

Trajectory Frequency Plot year 2004

Values (%) averaged between 0 m and 2000 m

Integrated from 0000 00 to 0000 00 00 (UTC)

Freq Release started at 0000 00 00 (UTC)

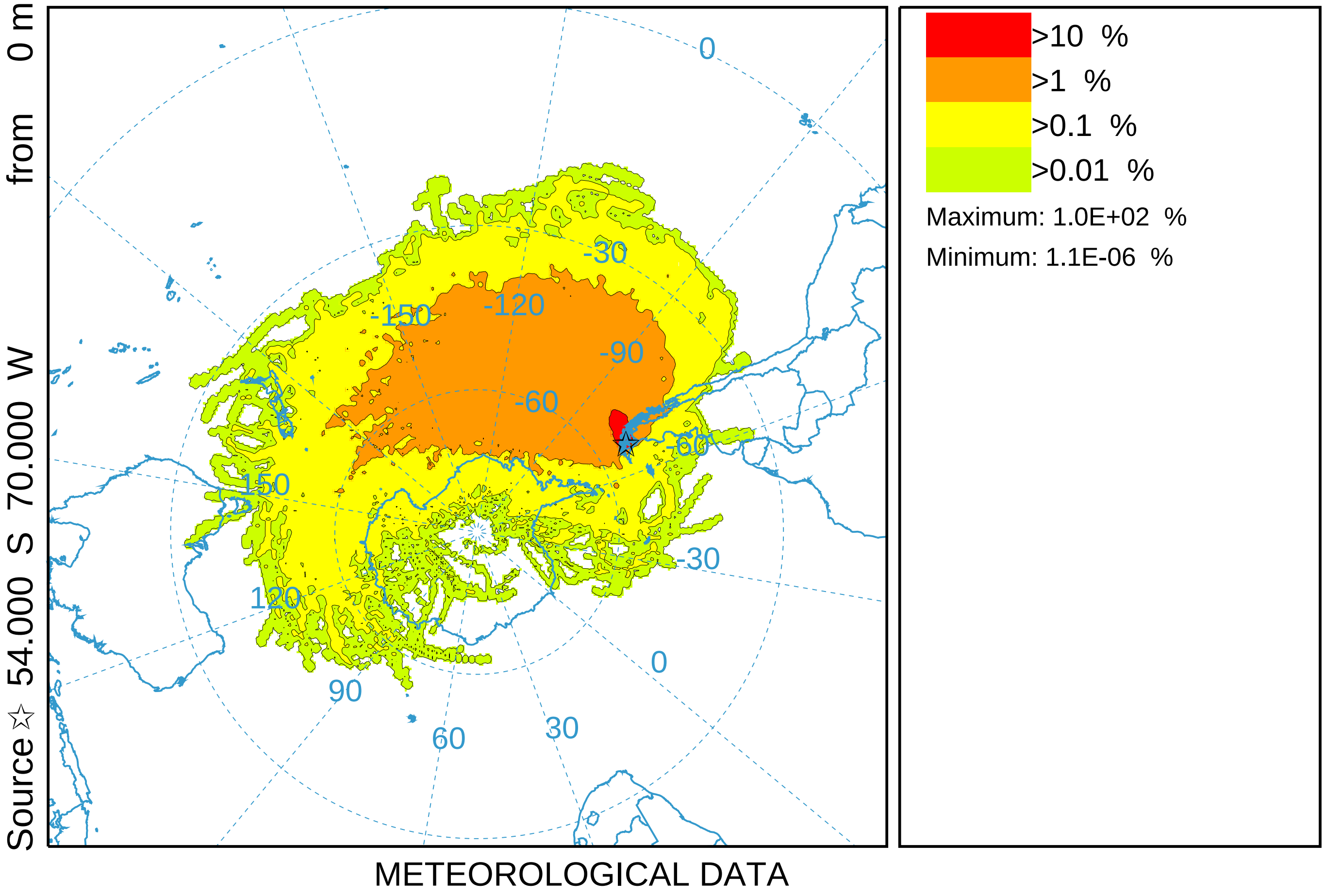

Trajectory Frequency Plot year 2005

Values (%) averaged between 0 m and 2000 m

Integrated from 0000 00 to 0000 00 00 (UTC)

Freq Release started at 0000 00 00 (UTC)

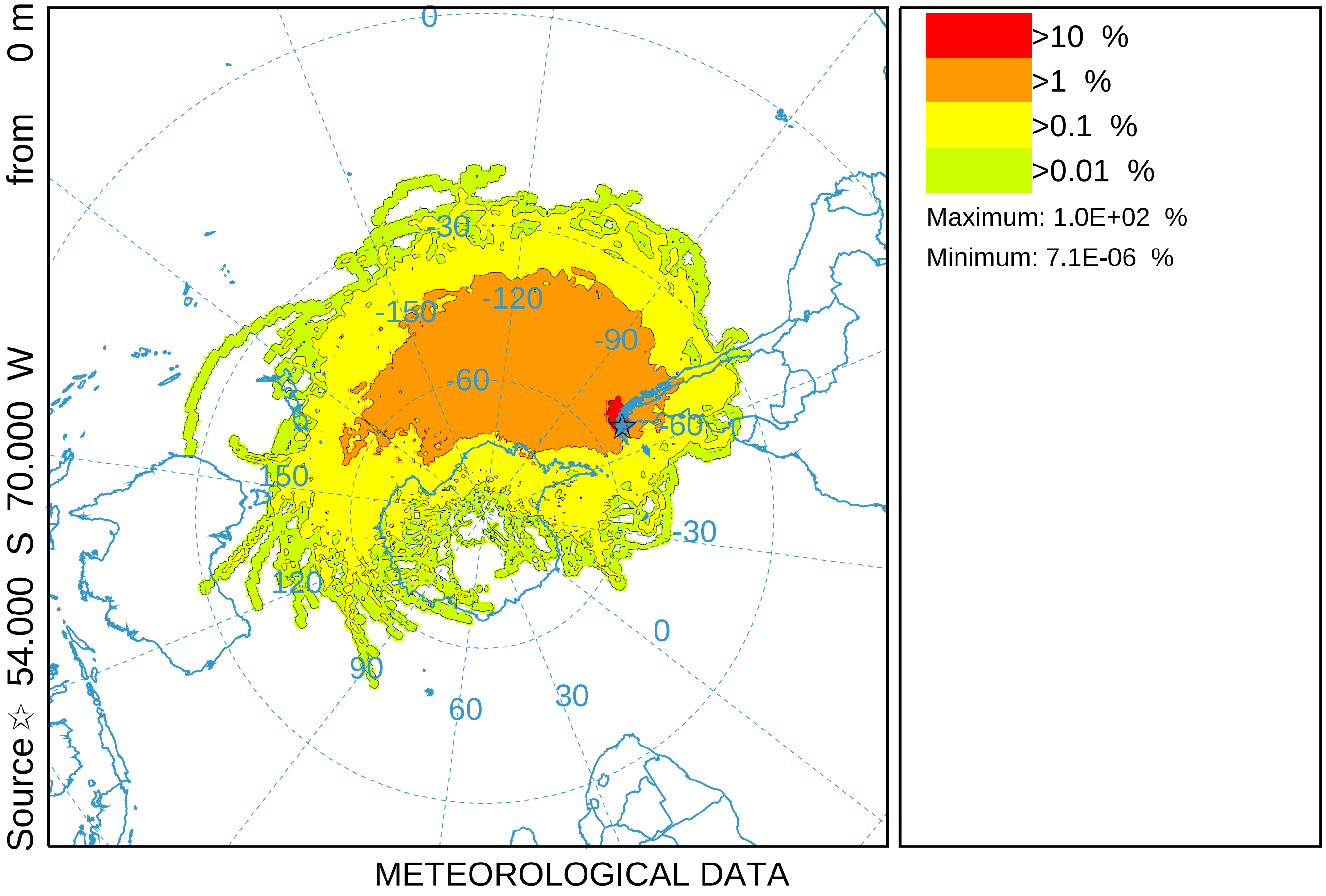

Trajectory Frequency Plot year 2006

Values (%) averaged between 0 m and 2000 m
Integrated from 0000 00 to 0000 00 00 (UTC)
Freq Release started at 0000 00 00 (UTC)

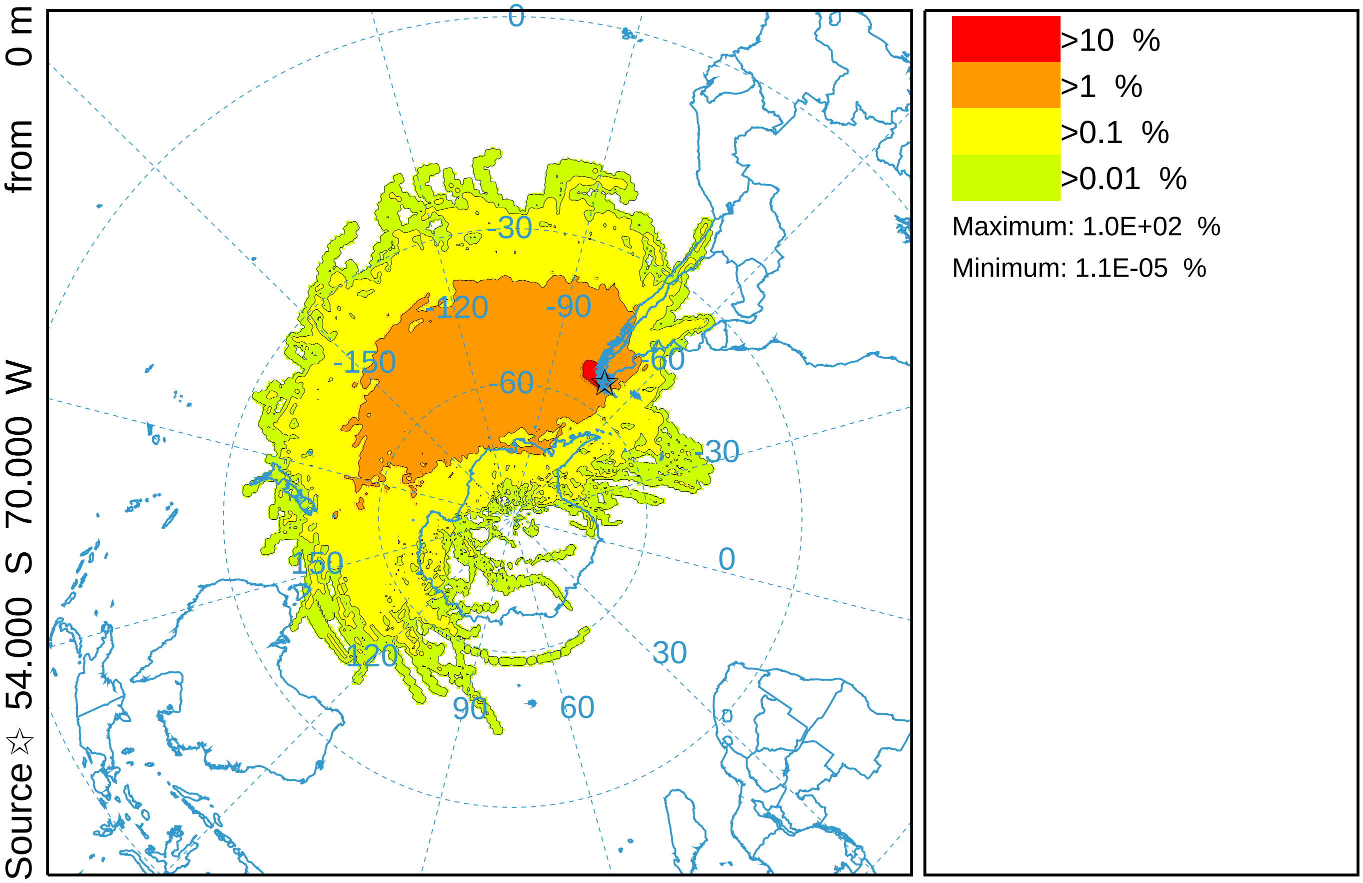

METEOROLOGICAL DATA

Trajectory Frequency Plot year 2007

Values (%) averaged between 0 m and 2000 m

Integrated from 0000 00 to 0000 00 00 (UTC)

Freq Release started at 0000 00 00 (UTC)

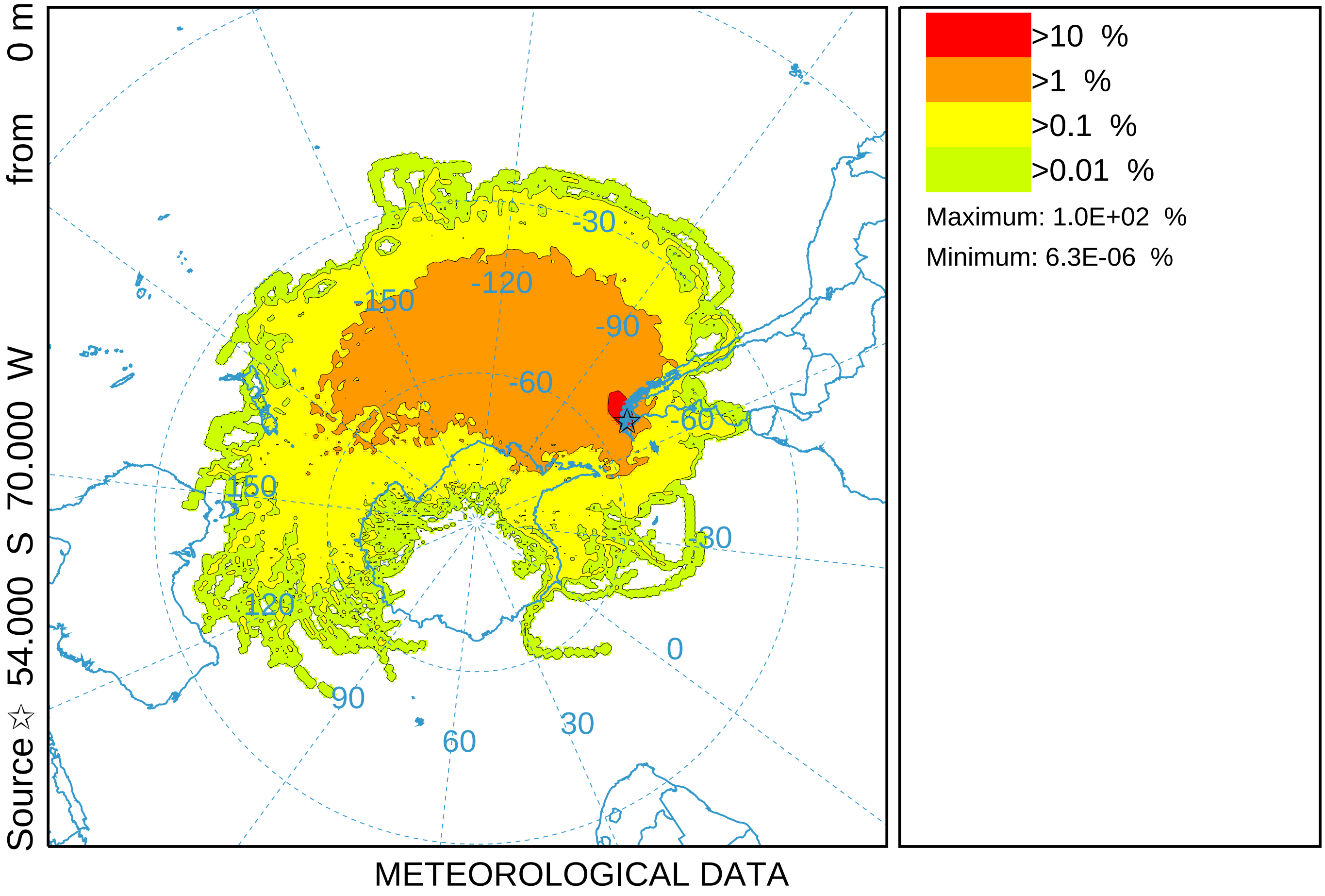

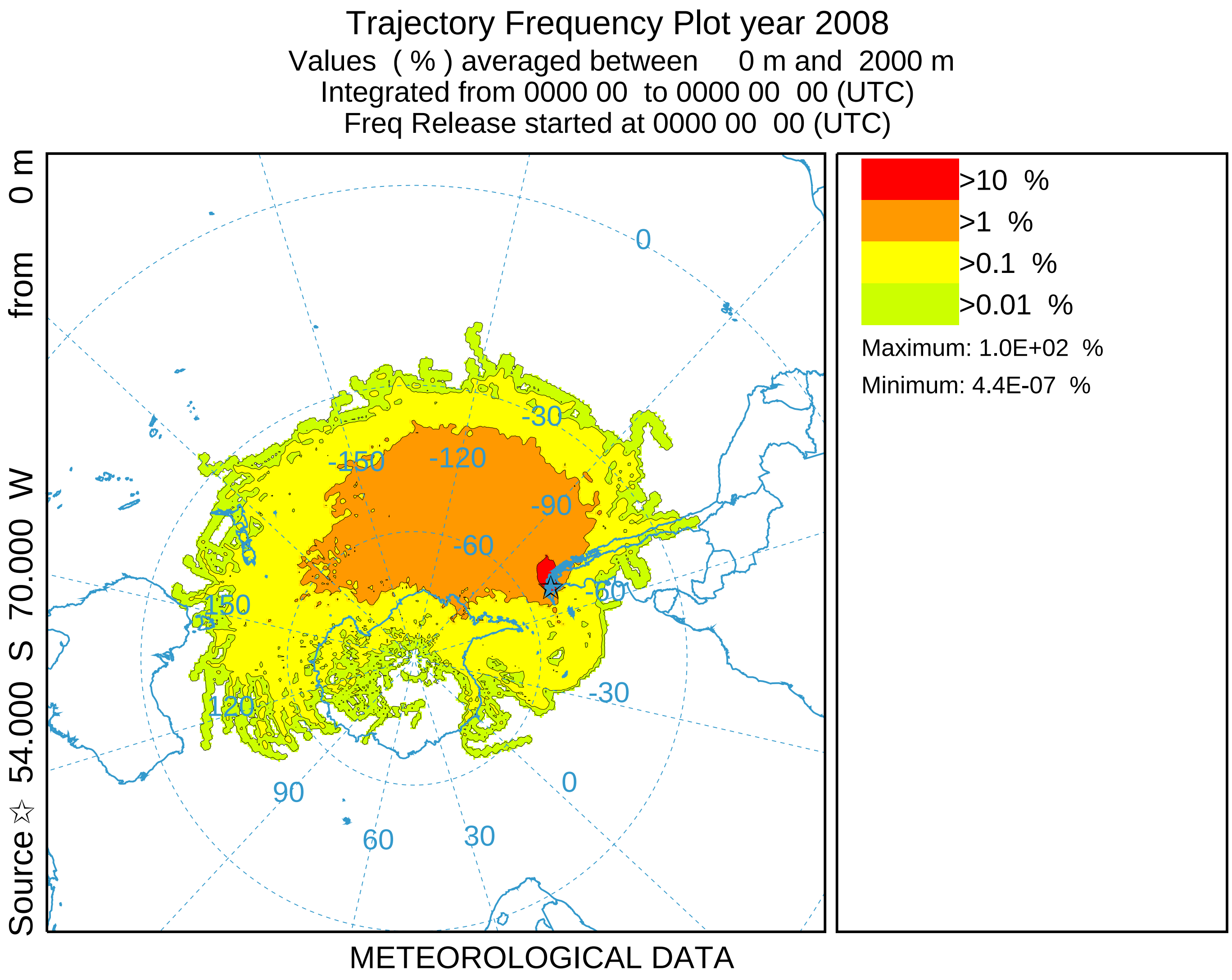
Trajectory Frequency Plot year 2008
Values (%) averaged between 0 m and 2000 m
Integrated from 0000 00 to 0000 00 00 (UTC)
Freq Release started at 0000 00 00 (UTC)
Source ☆ 54.000 S 70.000 W from 0 m
>10 %
>1 %
>0.1 %
>0.01 %
Maximum: 1.0E+02 %
Minimum: 4.4E-07 %
METEOROLOGICAL DATA

Trajectory Frequency Plot year 2009
Values (%) averaged between 0 m and 2000 m
Integrated from 0000 00 to 0000 00 00 (UTC)
Freq Release started at 0000 00 00 (UTC)

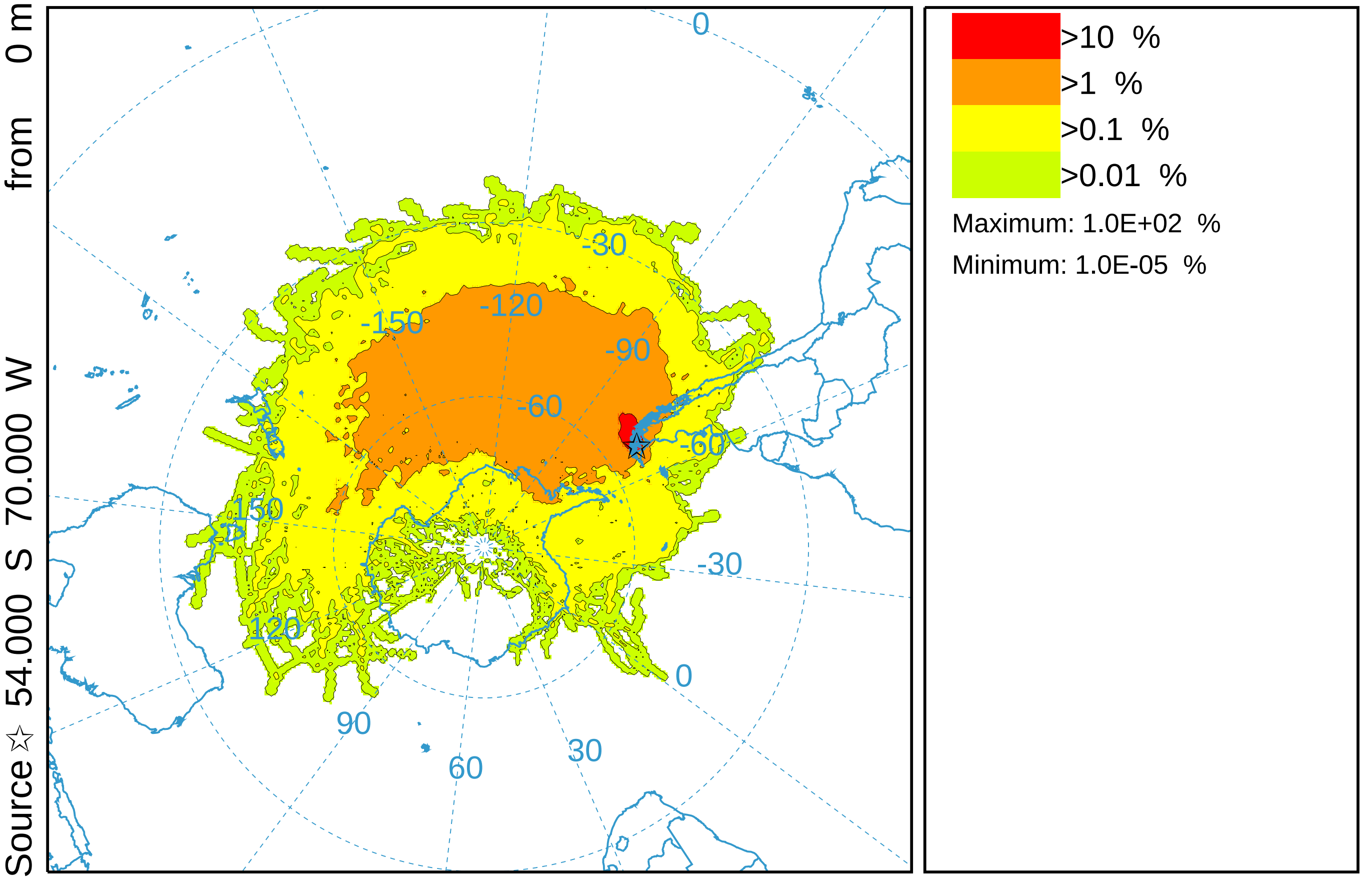

METEOROLOGICAL DATA

Trajectory Frequency Plot year 2010
Values (%) averaged between 0 m and 2000 m
Integrated from 0000 00 to 0000 00 00 (UTC)
Freq Release started at 0000 00 00 (UTC)

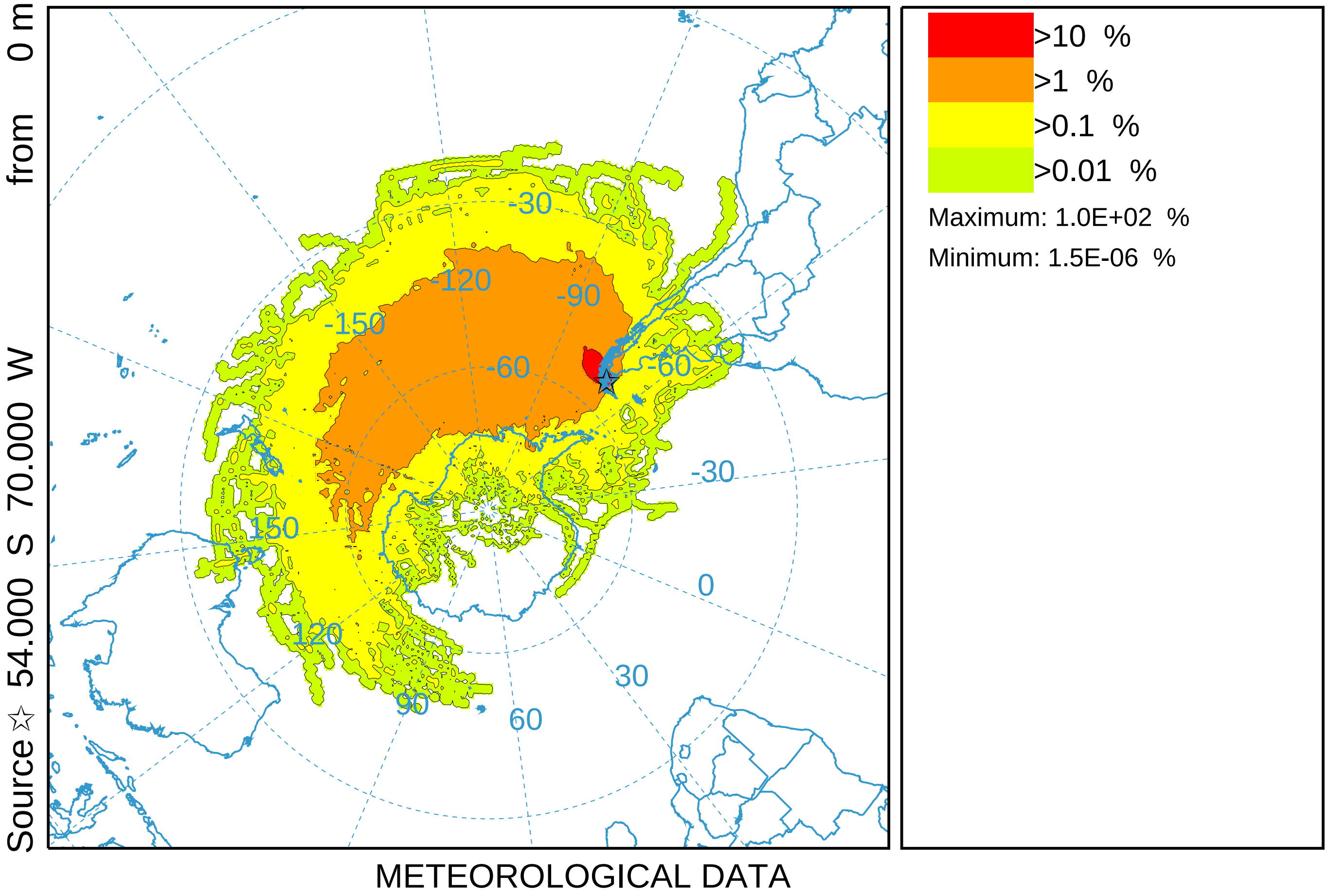

Trajectory Frequency Plot year 2011

Values (%) averaged between 0 m and 2000 m
Integrated from 0000 00 to 0000 00 00 (UTC)
Freq Release started at 0000 00 00 (UTC)

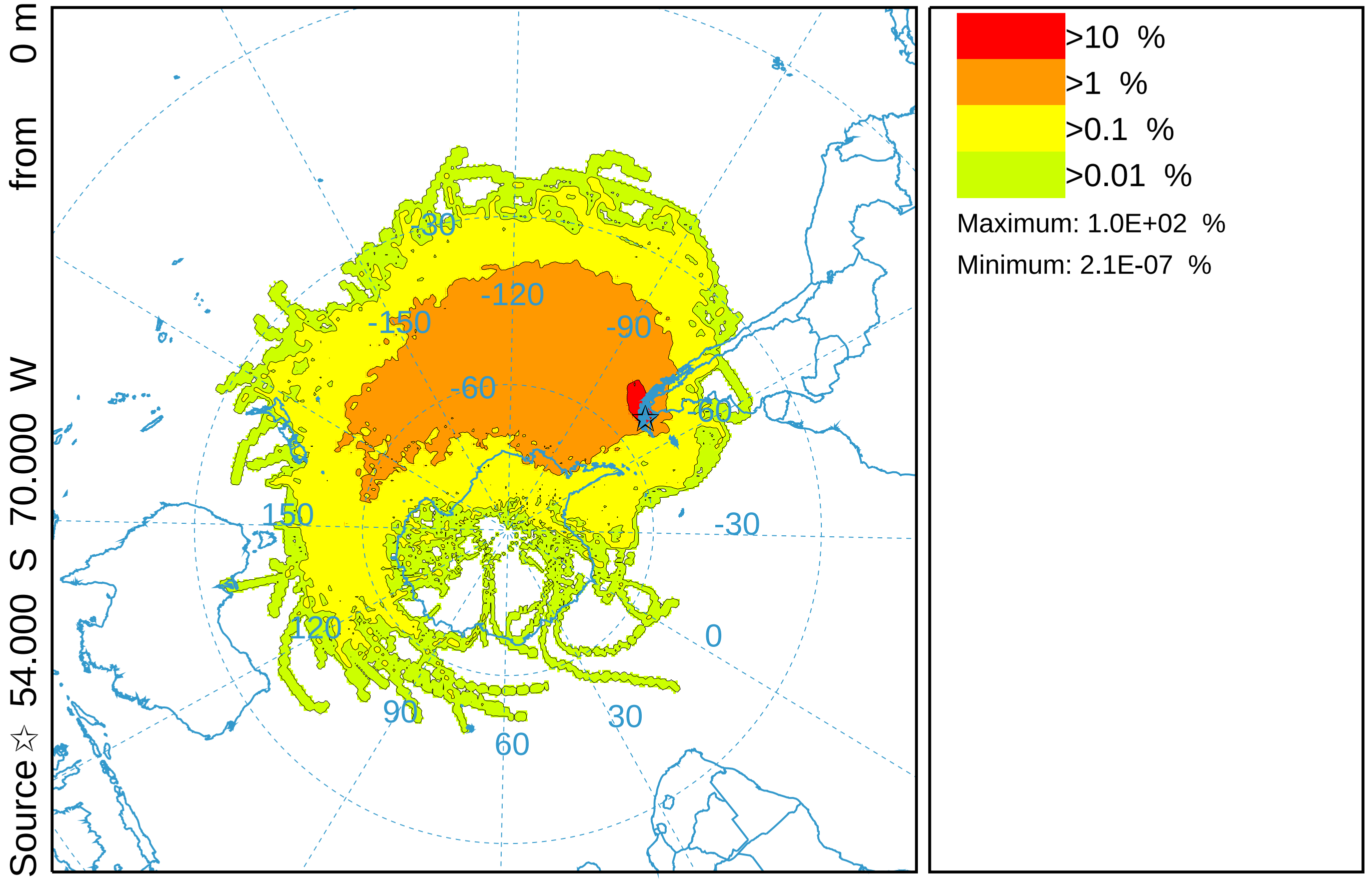

METEOROLOGICAL DATA

Trajectory Frequency Plot year 2012

Values (%) averaged between 0 m and 2000 m

Integrated from 0000 00 to 0000 00 00 (UTC)

Freq Release started at 0000 00 00 (UTC)

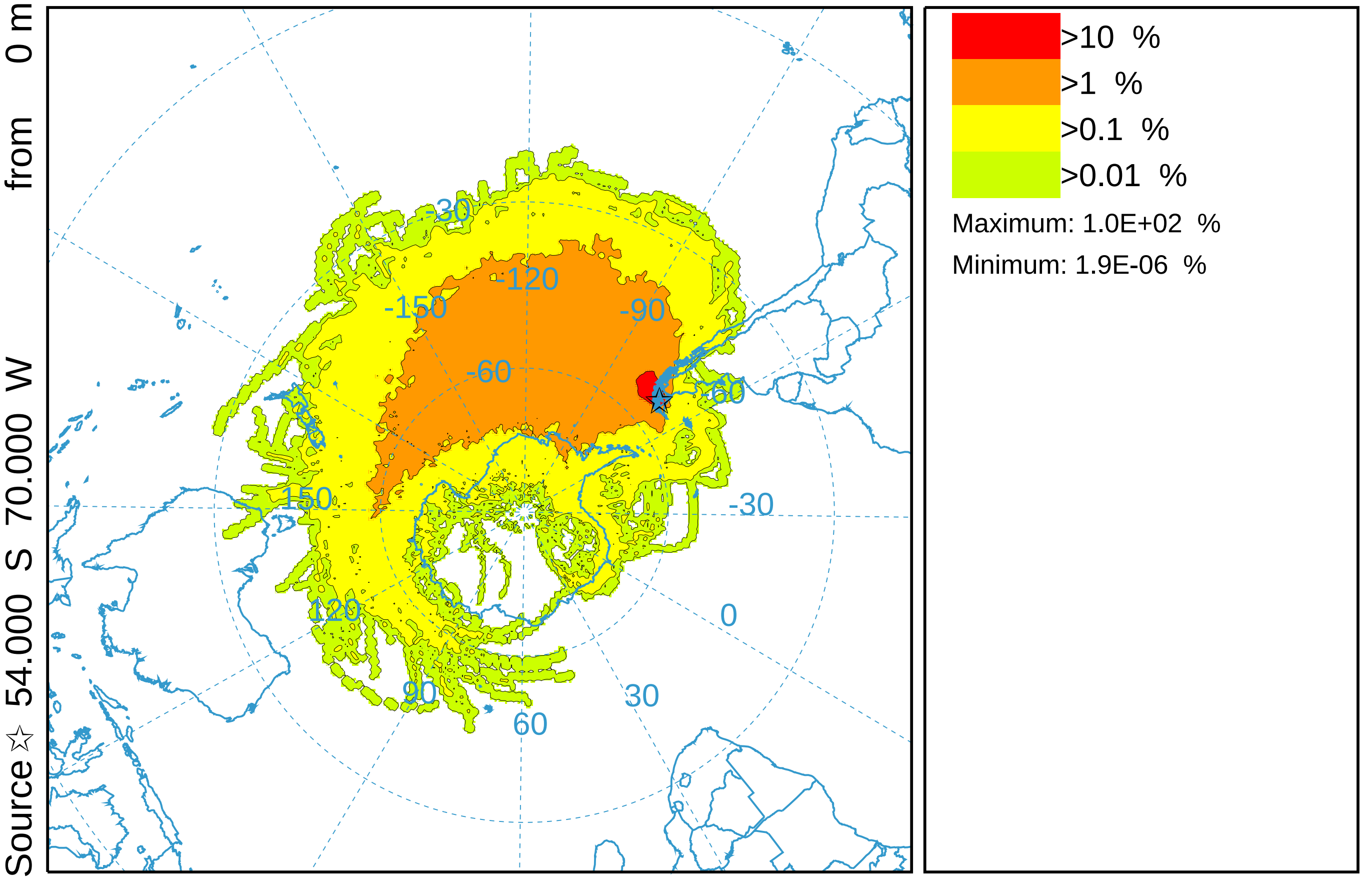

METEOROLOGICAL DATA
